# Nevirapine- Versus Lopinavir/Ritonavir-Based Initial Therapy for HIV-1 Infection among Women in Africa: A Randomized Trial

**DOI:** 10.1371/journal.pmed.1001236

**Published:** 2012-06-12

**Authors:** Shahin Lockman, Michael Hughes, Fred Sawe, Yu Zheng, James McIntyre, Tsungai Chipato, Aida Asmelash, Mohammed Rassool, Sylvester Kimaiyo, Douglas Shaffer, Mina Hosseinipour, Lerato Mohapi, Francis Ssali, Margret Chibowa, Farida Amod, Elias Halvas, Evelyn Hogg, Beverly Alston-Smith, Laura Smith, Robert Schooley, John Mellors, Judith Currier, the OCTANE (Optimal Combination Therapy After Nevirapine Exposure) ACTG A5208/OCTANE Study Team

**Affiliations:** 1 Brigham and Women's Hospital, Boston, Massachusetts, United States of America; 2 Harvard School of Public Health, Boston, Massachusetts, United States of America; 3 Botswana Harvard School of Public Health AIDS Initiative Partnership, Gaborone, Botswana; 4 Kenya Medical Research Institute/Walter Reed Project and US Military HIV Research Program, Kericho, Kenya; 5 Anova Health Institute, Johannesburg, South Africa; 6 University of Zimbabwe, Harare, Zimbabwe; 7 University of Witwatersrand, Johannesburg, South Africa; 8 Moi University Faculty of Health Sciences, Eldoret, Kenya; 9 Kamuzu Central Hospital, University of North Carolina Project, Lilongwe, Malawi; 10 Chris Hani Baragwanath Hospital, Johannesburg, South Africa; 11 Joint Clinical Research Centre, Kampala, Uganda; 12 University of Alabama at Birmingham Center for Infectious Disease Research in Zambia, Lusaka; 13 University of Kwazulu-Natal, Durban, South Africa; 14 University of Pittsburgh, Pittsburgh, Pennsylvania, United States of America; 15 Social & Scientific Systems, Silver Spring, Maryland, United States of America; 16 National Institutes of Health, Bethesda, Maryland, United States of America; 17 Frontier Science and Technology Research Foundation, Amherst, Massachusetts, United States of America; 18 University of California San Diego, San Diego, California, United States of America; 19 University of California Los Angeles, Los Angeles, California, United States of America; San Francisco General Hospital, United States of America

## Abstract

**Background:**

Nevirapine (NVP) is widely used in antiretroviral treatment (ART) of HIV-1 globally. The primary objective of the AA5208/OCTANE trial was to compare the efficacy of NVP-based versus lopinavir/ritonavir (LPV/r)-based initial ART.

**Methods and Findings:**

In seven African countries (Botswana, Kenya, Malawi, South Africa, Uganda, Zambia, and Zimbabwe), 500 antiretroviral-naïve HIV-infected women with CD4<200 cells/mm^3^ were enrolled into a two-arm randomized trial to initiate open-label ART with tenofovir (TDF)/emtricitabine (FTC) once/day plus either NVP (*n* = 249) or LPV/r (*n* = 251) twice/day, and followed for ≥48 weeks. The primary endpoint was time from randomization to death or confirmed virologic failure ([VF]) (plasma HIV RNA<1 log_10_ below baseline 12 weeks after treatment initiation, or ≥400 copies/ml at or after 24 weeks), with comparison between treatments based on hazard ratios (HRs) in intention-to-treat analysis. Equivalence of randomized treatments was defined as finding the 95% CI for HR for virological failure or death in the range 0.5 to 2.0. Baseline characteristics were (median): age = 34 years, CD4 = 121 cells/mm^3^, HIV RNA = 5.2 log_10_copies/ml. Median follow-up = 118 weeks; 29 (6%) women were lost to follow-up. 42 women (37 VFs, five deaths; 17%) in the NVP and 50 (43 VFs, seven deaths; 20%) in the LPV/r arm reached the primary endpoint (HR 0.85, 95% CI 0.56–1.29). During initial assigned treatment, 14% and 16% of women receiving NVP and LPV/r experienced grade 3/4 signs/symptoms and 26% and 22% experienced grade 3/4 laboratory abnormalities. However, 35 (14%) women discontinued NVP because of adverse events, most in the first 8 weeks, versus none for LPV/r (*p*<0.001). VF, death, or permanent treatment discontinuation occurred in 80 (32%) of NVP and 54 (22%) of LPV/r arms (HR = 1.7, 95% CI 1.2–2.4), with the difference primarily due to more treatment discontinuation in the NVP arm. 13 (45%) of 29 women tested in the NVP versus six (15%) of 40 in the LPV/r arm had any drug resistance mutation at time of VF.

**Conclusions:**

Initial ART with NVP+TDF/FTC demonstrated equivalent virologic efficacy but higher rates of treatment discontinuation and new drug resistance compared with LPV/r+TDF/FTC in antiretroviral-naïve women with CD4<200 cells/mm^3^.

**Trial registration:**

ClinicalTrials.gov NCT00089505

*Please see later in the article for the Editors' Summary*

## Abstract 2

In a randomized control trial, Shahin Lockman and colleagues compare nevirapine-based therapy with lopinavir/ritonavir-based therapy for HIV-infected women without previous exposure to antiretroviral treatment.

## Editors' Summary

**Background:**

About 34 million people (mostly living in low- or middle-income countries) are currently infected with HIV, the virus that causes AIDS. HIV destroys CD4 lymphocytes and other immune cells, leaving infected individuals susceptible to other infections. Early in the AIDS epidemic, most HIV-infected people died within 10 years of infection. Then, in 1996, antiretroviral therapy (ART)—cocktails of drugs that attack different parts of HIV—became available. For people living in affluent countries, HIV/AIDS became a chronic condition. But, because ART was expensive, for people living in developing countries, HIV/AIDS remained a fatal illness. In 2006, the international community set a target of achieving universal access to ART by 2010 and, although this target has not been reached, by the end of 2010, 6.6 million of the estimated 15 million people in need of ART in developing countries were receiving one of the ART regimens recommended by the World Health Organization (WHO) in its 2010 guidelines.

**Why Was This Study Done?:**

A widely used combination for the initial treatment of HIV-infected people (particularly women) in resource-limited settings is tenofovir and emtricitabine (both nucleotide reverse transcriptase inhibitors; reverse transcriptase is essential for HIV replication) and nevirapine (NVP, a non-nucleoside reverse transcriptase inhibitor). However, little is known about the efficacy of this NVP-based ART combination. Moreover, its efficacy and toxicity has not been compared with regimens containing lopinavir/ritonavir (LPV/r). LPV/r, which inhibits the viral protease that is essential for HIV replication, is available in resource-limited settings but is usually reserved for second-line treatment. LPV/r-based ART is more expensive than NVP-based ART but if it were more effective or better tolerated than NVP-based ART, then first-line treatment with LPV/r-based ART might be cost-effective in resource-limited settings. Conversely, evidence of the clinical equivalence of NVP-based and LPV/r-based ART would provide support for NVP-based ART as an initial therapy. In this randomized equivalence trial, the researchers compare the efficacy and toxicity of NVP-based and LVP/r-based initial therapy for HIV infection among antiretroviral-naïve African women. In a randomized trial, patients are assigned different treatments by the play of chance and followed to compare the effects of these treatments; an equivalence trial asks whether the effects of two treatments are statistically equivalent.

**What Did the Researchers Do and Find?:**

The researchers followed 500 antiretroviral-naïve HIV-infected women with a low CD4 cell count living in seven African countries, half of whom received NVP-based ART and half of whom received LPV/r-based ART, for an average of 118 weeks and recorded the time to virologic failure (the presence of virus in the blood above pre-specified levels) or death among the participants. Forty-two women in the NVP arm reached this primary endpoint (37 virologic failures and five deaths) compared to 50 women in the LPV/r arm (43 virologic failures and seven deaths), a result that indicates equivalent virologic efficacy according to preset statistical criteria. During the initial assigned treatment, similar proportions of women in both treatment arms developed serious drug-related signs and symptoms and laboratory abnormalities. However, whereas 14% of the women in the NVP arm discontinued treatment because of adverse effects, none of the women in the LPV/r arm discontinued treatment. Finally, nearly half of the women tested in the NVP arm but only 15% of the women tested in the LVP/r arm had developed any drug resistance at the time of virologic failure.

**What Do These Findings Mean?:**

These findings indicate that, among HIV-infected, treatment-naïve African women, initial NVP-based ART is as effective as LPV/r-based ART in terms of virologic failure and death although more women in the NVP arm discontinued treatment or developed new drug resistance than in the LPV/r arm. Several limitations of this study may affect the accuracy of these findings. In particular, some of the study participants may have been exposed to single-dose NVP during childbirth to prevent mother-to-child transmission of HIV; in a parallel randomized trial, the researchers found that LPV/r-based ART was superior to NVP-based ART among women with prior exposure to single-dose NVP. Moreover, the duration of the current study means the long-term effects of the two treatments cannot be compared. Nevertheless, these findings support the WHO recommendation of NVP-based ART with careful early toxicity monitoring as an initial affordable and effective HIV treatment regiment in resource-limited settings, until access to better-tolerated and more potent regimens is possible.

**Additional Information:**

Please access these Web sites via the online version of this summary at http://dx.doi.org/10.1371/journal.pmed.1001236.

- Information is available from the US National Institute of Allergy and Infectious Diseases on all aspects of HIV infection and AIDS
- NAM/aidsmap provides basic information about HIV/AIDS, and summaries of recent research findings on HIV care and treatment (in several languages)
- Information is available from Avert, an international AIDS charity on many aspects of HIV/AIDS, including detailed information on HIV treatment and care (in English and Spanish)
- WHO provides information about universal access to AIDS treatment (in English, French and Spanish); its 2010 ART guidelines can be downloaded
- More information about this trial, the OCTANE trial, is available
- MedlinePlus provides detailed information about nevirapine and lopinavir/ritinovir (in English and Spanish)
- Patient stories about living with HIV/AIDS are available through Avert; the nonprofit website Healthtalkonline also provides personal stories about living with HIV, including stories about taking anti-HIV drugs and the challenges of anti-HIV drugs

## Introduction

Most of the 33 million people living with HIV reside in resource-limited settings (RLS), and more than 5 million are receiving antiretroviral treatment (with the number treated continuing to increase). Globally, the vast majority of antiretroviral treatment (ART) is provided by general practitioners (and not HIV specialists), using a public health approach. One of the primary ART regimens recommended in 2010 by the World Health Organization (WHO) for initial treatment of HIV-infected persons is composed of tenofovir (TDF), emtricitabine (FTC), and nevirapine (NVP) [1]. This particular combination is rapidly becoming one of the most commonly used antiretroviral treatment regimens worldwide, particularly among women. However, minimal data regarding the efficacy of this combination exist. Furthermore, many practitioners have expressed concern about the potency of this particular regimen, given early data emanating from small studies and observational cohorts suggesting possible suboptimal efficacy [2],[3]. In addition, the efficacy and toxicity of initial ART containing NVP have not been compared prospectively with regimens containing lopinavir/ritonavir (LPV/r), the most commonly available boosted protease inhibitor (PI) in RLS at this time. If initial ART with a PI such as LPV/r were found to be much more effective or better tolerated than ART with the commonly used NVP-based regimens, then first-line treatment with a PI could in fact be cost-effective [4]; conversely equivalence of NVP and LPV/r would provide some reassurance regarding NVP-based ART as an initial therapy as recommended by WHO guidelines. Rigorous data regarding the efficacy (and comparative efficacy) of recommended and frequently used ART regimens is of vital importance to HIV programs worldwide.

A particular concern among women starting ART is that prior exposure to single dose NVP (sdNVP) during labor for preventing mother-to-child transmission (PMTCT) of HIV may affect the relative efficacy of these two regimens. We therefore conducted A5208/OCTANE, a study consisting of two parallel randomized trials of initial three-drug ART that included LPV/r versus NVP among women in Africa. For one trial among women with prior sdNVP exposure, we previously reported the superior efficacy of LPV/r versus NVP [5]. In this report, we present the results from women without prior sdNVP exposure.

## Methods

### Ethics

The study was approved by all overseeing local Institutional Review Boards (IRBs)/Ethics Committees, as well as IRBs at the following US institutions: Harvard School of Public Health, Walter Reed Army Institute of Research, Indiana University, University of North Carolina, Case Western Reserve University, University of Alabama, and University of California San Diego. Participants provided written informed consent.

### Study Design, Participants

OCTANE comprised two concurrent, randomized open-label ART trials (see Texts S1 and S2 for trial details). Trial 1 was conducted among 243 women who had ingested sdNVP ≥6 mo prior to enrollment (results published [5]), and was designed to test the superiority of LPV/r over NVP due to the hypothesis that prior sdNVP exposure would result in persistent NVP drug resistance. Trial 2, described in this report, was conducted among 502 women without prior sdNVP exposure. Trial 2 was designed to test the equivalence of LPV/r with NVP on the basis of the hypothesis that the efficacy of NVP- and LPV/r-based ART would be similar. To permit optimal interpretation of trial 1, interim trial 2 virologic failure (VF)/death results after partial follow-up (through 6 October 2008) were included in the published trial 1 paper [5]. This current report describes detailed and final trial 2 results, using data from a substantially longer period of follow-up (through 30 September 2009).

Participants were consenting, HIV-1-infected adult women who were not pregnant or breastfeeding, from ten African sites (three in South Africa; two in Kenya; and one each in Zimbabwe, Botswana, Zambia, Malawi, and Uganda). Participants had screening CD4+ cell count (CD4)<200 cells/mm^3^ and trial 2 participants were antiretroviral-naïve, except that up to 10 wk of prior zidovudine (ZDV) were permitted (as short-course ZDV was included in mother-to-child HIV transmission prevention regimens in some countries, and is not associated with significant development of major drug resistance mutations). Lack of prior sdNVP exposure was based on participant recall and self-report, in combination with one or more of the following when available: no prior pregnancy or pregnancy prior to the use of sdNVP in country; HIV diagnosis after pregnancy; pre-delivery/postpartum or other relevant records reviewed and no sdNVP recorded; patient confirmation of no prior sdNVP after being shown tablet. Additional eligibility criteria included estimated creatinine clearance ≥60 ml/minute; hemoglobin ≥7.0 g/dl; absolute neutrophil count ≥750 cells/mm^3^; alanine aminotransferase (ALT), aspartate aminotransferase (AST), alkaline phosphatase, and total bilirubin (each ≤2.5 times the upper limit of normal); absence of pregnancy/breastfeeding; absence of serious illness or tuberculosis treatment within the prior 30 d; and Karnofsky score ≥70. Participants were followed until 48 wk after the last enrollment.

Women were randomized to open-label LPV/r (400 mg/100 mg) one capsule (or tablet) twice daily plus TDF/FTC (300 mg/200 mg) one tablet once daily; or to NVP 200 mg twice daily plus TDF/FTC 300/200 mg once daily (after 14-d lead-in with NVP 200 mg once daily). TDF/FTC was co-formulated in Truvada. LPV/r was initially supplied as Kaletra capsules, then heat-stable Aluvia tablets. Participants could switch from NVP to second-line treatment with LPV/r or vice versa (and ZDV and didanosine were provided), in cases of VF (at the discretion of local investigators/participants) or toxicity. EFV was temporarily substituted for NVP and LPV/r during rifampin-containing tuberculosis treatment. Randomization was computer-generated using balanced block randomization (block size 4) stratified by screening CD4 count (< or ≥50 cells/mm^3^). Randomized assignment was provided electronically from a remote, central Data Management Center in the US back to each clinical site. While the study was being conducted, access to the block size and the sequence of treatment assignments was restricted to Data Center staff who set up the randomization.

### Data Collection, Follow-up, and Laboratory Analyses

Study visits (with safety laboratory/clinical assessment) occurred at 2, 4, 8, 12, 16, and 24 wk after treatment initiation and every 12 wk thereafter. HIV-1 RNA (Roche Amplicor Monitor V1.5) and CD4 were assessed at entry, then every 12 wk. Lipids (fasting or non-fasting) were evaluated at entry, 24, and 48 wk, then every 48 wk.

A random subgroup of 126 pre-therapy samples were tested for HIV-1 drug resistance using the ViroSeq HIV-1 Genotyping System (V2.0), with data analyzed using ViroSeq Genotyping Software V2.7 (both Celera Diagnostics). In addition, pre-therapy and time-of-failure samples were tested from all patients experiencing virologic failure.

### Protocol-Specified Toxicity Management

ART was generally held in the event of potentially treatment-related grade ≥3 toxicity. However, more conservative toxicity management was followed for suspected NVP or TDF toxicity. Specifically, the presence of any of the following in combination with any rash (and no other clear explanation) required permanent discontinuation of NVP: systemic, allergic, or mucosal symptoms; or elevated ALT, AST, creatinine, or eosinophils. NVP was also permanently discontinued in participants experiencing grade ≥2 elevations in ALT or AST (or with worsening of LFTs by ≥1 grade in combination with hepatitis signs/symptoms). TDF was permanently discontinued if confirmed estimated creatinine clearance decreased to <50 ml/min, in absence of alternate cause.

Women becoming pregnant after enrollment were continued on study treatment, with ZDV substituted for TDF during pregnancy unless a contraindication to ZDV existed.

### Statistical Analysis

The primary endpoint was time to VF or death. VF was defined as plasma HIV-1 RNA<1 log_10_ copies/ml below baseline 12 wk after treatment initiation, or HIV-1 RNA ≥400 copies/ml at ≥24 wk after initiation; in both cases with confirmation by a second measurement meeting the respective criterion.

The primary analysis was intention to treat, including all follow-up irrespective of changes in ART (but excluded women failing to start study treatment, per protocol). Trial 2 was designed to evaluate the equivalence of LPV/r- and NVP-based therapies: equivalence was to be established if the two-sided 95% CI for the hazard ratio (HR) for VF/death was entirely within the range 0.5–2.0.

The Kaplan-Meier method was used to describe the cumulative proportion of participants experiencing VF or death by time. Cox proportional hazards models, stratified by screening CD4 (<50 versus ≥50 cells/mm^3^), were used to estimate the HR (95% CIs) of reaching an endpoint comparing arms.

In secondary analysis, we also evaluated equivalence at each of week 48, 96, and 144 with respect to the absolute difference between randomized treatments in the Kaplan-Meier estimate of the percentage of subjects reaching an endpoint by these times, on the basis of the common definition that the 95% CI for the absolute difference was entirely within the range −10% to +10%.

Adjusted analyses were undertaken by including each of the following variables in turn into the proportional hazards model, which also included randomized treatment: existence or not of written documentation of no prior sdNVP exposure, prior ZDV use, site, and baseline age, CD4 count, HIV-1 RNA, and WHO stage. Subgroup analyses were undertaken by also including the treatment interaction with each of these variables in the model. These variables were pre-specified in the analysis plan prior to the first interim analysis.

Discontinuation of initial assigned regimen was defined as permanent discontinuation of either LPV/r or NVP. In secondary as-treated analysis, VF was considered to have occurred on the randomized regimen only if the initial failing HIV-1 RNA measurement was obtained on or before the day of last dose of the initial regimen. Analyses of adverse events (AEs) were restricted to the period during which participants received initial NVP or LPV/r. Linear regression was used to compare changes in CD4 count and lipids, adjusted for baseline count/level.

The original planned sample size for trial 2 was 400. During closed reviews by an independent Data and Safety Monitoring Board (DSMB), it was noted that the proportion of women experiencing a primary endpoint might be lower than anticipated (14% versus anticipated 20% with median follow-up of 72 wk). The sample size was therefore increased to 500, which, with an associated increase in expected median follow-up to 108 wk, was expected to provide about 77% probability of completing the study showing equivalence (95% CI for the primary endpoint HR entirely within the range 0.5–2.0) assuming no true difference between treatments.

Review by the DSMB occurred every 6–12 mo. The protocol stated that early termination of the trial for efficacy would generally only be considered if equivalence was established to a very high level of evidence, specifically a 99.9% CI unadjusted for interim analyses within the narrow range of 0.75–1.33, or if a difference between randomized arms was established with substantial evidence, specifically a 99.9% CI excluding a HR of one; because trial 2 was not stopped early, standard 95% CIs can therefore be used at the end of the study [6]. *p*-Values are two-sided, for the test of no difference between randomized treatments.

## Results

**Figure 1:**
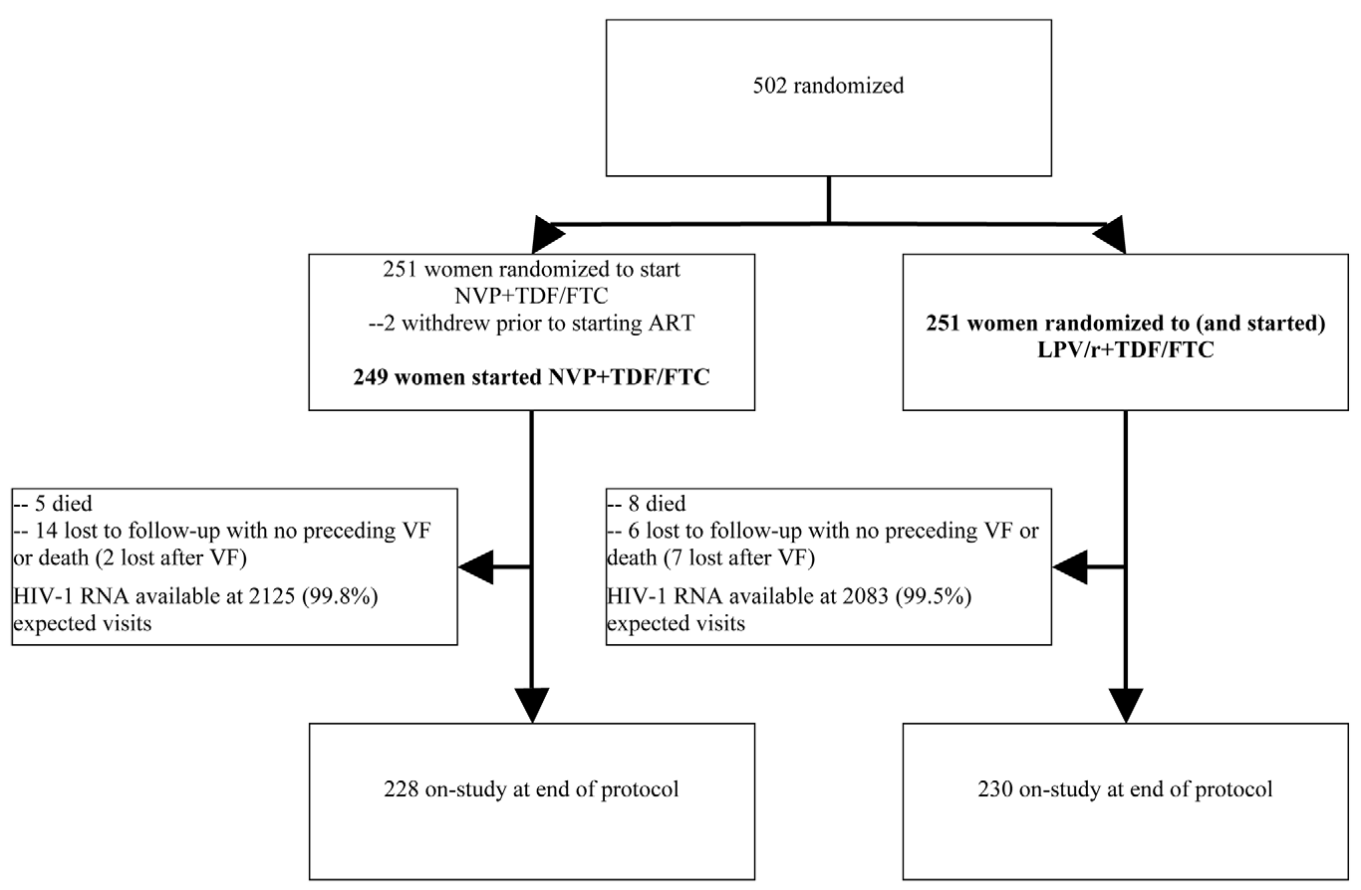
Consort diagram. Per protocol, participants were not mandated to switch regimens in instances of VF (this decision was left to the discretion of study staff and participants). More women experienced VF in the LPV/r arm (43 or 17%) compared with the NVP arm (37 or 15%) in ITT analysis; however, a higher proportion of women experiencing VF in the NVP arm were switched to LPV/r compared with vice versa.

A total of 502 women (target 500) enrolled and were randomized to NVP+TDF/FTC versus LPV/r+TDF/FTC from November 2005–July 2008 (37–81 participants/site) (Figure 1). Two women (in the NVP arm) never started study treatment and were excluded from all analyses. Therefore, results from 500 women (249 randomized to receive NVP, 251 to LPV/r) were included. One participant had previously taken sdNVP but was mistakenly enrolled (in the LPV/r arm); her data are included.

Median age, CD4 count, and HIV-1 RNA at baseline were 34 y, 121 cells/mm^3^, and 5.2 log_10_ copies/ml, respectively (Table 1), and were similar in both arms. Evidence (in addition to self-report) for lack of prior exposure to sdNVP was available for 469 (94%) women (most commonly, no pregnancy or occurrence of pregnancy prior to local use of sdNVP [*n* = 272] or HIV diagnosis after pregnancy [*n* = 168]). Seven (1%) women reported prior ZDV for preventing mother-to-child transmission. Of the random sample of 126 pre-therapy samples tested for HIV-1 drug resistance (60 from the NVP and 66 from the LPV/r arms), baseline NVP resistance (K103N) was detected in only one (0.8%) sample (Table 1). No nucleoside analog reverse-transcriptase inhibitor (NRTI) or major protease-resistance mutations were detected at baseline, although minor protease-resistance mutations were present in 100% of samples. Subtype C virus was found in 85 (71%).

**Table 1:** Selected characteristics of OCTANE trial 2 participants at baseline.

Characteristics	Statistics Categories	NVP (*n* = 249)	LPV/r (*n* = 251)	Total (*n* = 500)
Age at randomization (y)	Median (P10, P90)	35 (26, 44)	34 (25, 45)	34 (26, 45)
Race/ethnicity^a^	Black	249 (100%)	251 (100%)	500 (100%)
Baseline CD4 count (cells/mm^3^)	Median (P10, P90)	121 (40, 208)	121 (32, 201)	121 (38, 204)
	<50cells/mm^3^	32 (13%)	36 (14%)	68 (14%)
	≥200 cells/mm^3^ b	31 (12%)	27 (11%)	58 (12%)
HIV-1 RNA (log_10_ copies/ml)	Median (P10, P90)	5.16 (4.16, ≥5.88)	5.15 (4.28, 5.82)	5.15 (4.21, 5.86)
HIV-1 RNA (copies/ml)	≥750,000	26 (10%)	22 (9%)	48 (10%)
WHO HIV stage	Clinical stage I	97 (39%)	93 (37%)	190 (38%)
	Clinical stage II	72 (29%)	67 (27%)	139 (28%)
	Clinical stage III	73 (29%)	80 (32%)	153 (31%)
	Clinical stage IV	7 (3%)	11 (4%)	18 (4%)
Hepatitis B surface antigen	Positive	18 (7%)	17 (7%)	35 (7%)
	Not tested at entry	0	2	2
Pre-entry ZDV (≤10 wk)		4 (2%)	3 (1%)	7 (1%)
Participant's self-report of prior ingestion of sdNVP	Yes	0	1 (0.4%)	1 (0.2%)
Supporting evidence for lack of prior sdNVP exposure	Yes	232 (93%)	237 (94%)	469 (94%)
Sequence results		60	66	126
HIV subtype	A1	9 (16%)	12 (19%)	21 (18%)
	A2	0 (0%)	1 (1%)	1 (1%)
	C	42 (75%)	43 (68%)	85 (71%)
	D	4 (7%)	4 (6%)	8 (7%)
	G	1 (2%)	0 (0%)	1 (1%)
	Complex recombination	0 (0%)	3 (5%)	3 (3%)
	Tested/no result	4	3	7
Nevirapine resistance		1 (2%)^c^	0	1 (0.8%)

Percentages use number of participants with a result as the denominator.

a Race was collected as it could potentially be related to treatment response and was reported by the site investigators.

b All women had screening CD4<200 cells/mm^3^, but at study entry, 58 women had CD4≥200 cells/mm^3^.

c K103N.

20 women (4%; 14 assigned to NVP and six to LPV/r) were lost to follow-up without first experiencing a primary endpoint (*p* = 0.08). Nine additional women were lost to follow-up after experiencing VF (two in the NVP and seven in the LPV/r arms), leading to overall loss to follow-up of 6%. The median duration of follow-up on initial assigned treatment was 109 wk (95 wk in the NVP and 119 wk in the LPV/r arms), and the overall median duration of follow-up in the study was 118 wk. At each scheduled evaluation, completed adherence questionnaires were available for ≥78% of participants. Adherence to initial study treatment was lowest at week 24, when 84% of women reported not missing any medications during the past month (88% versus 81% for the NVP and LPV/r arms). Adherence was 87%–92% at all other weeks.

### Primary Endpoint: VF or Death, Intent-to-Treat Analysis

**Figure 2:**
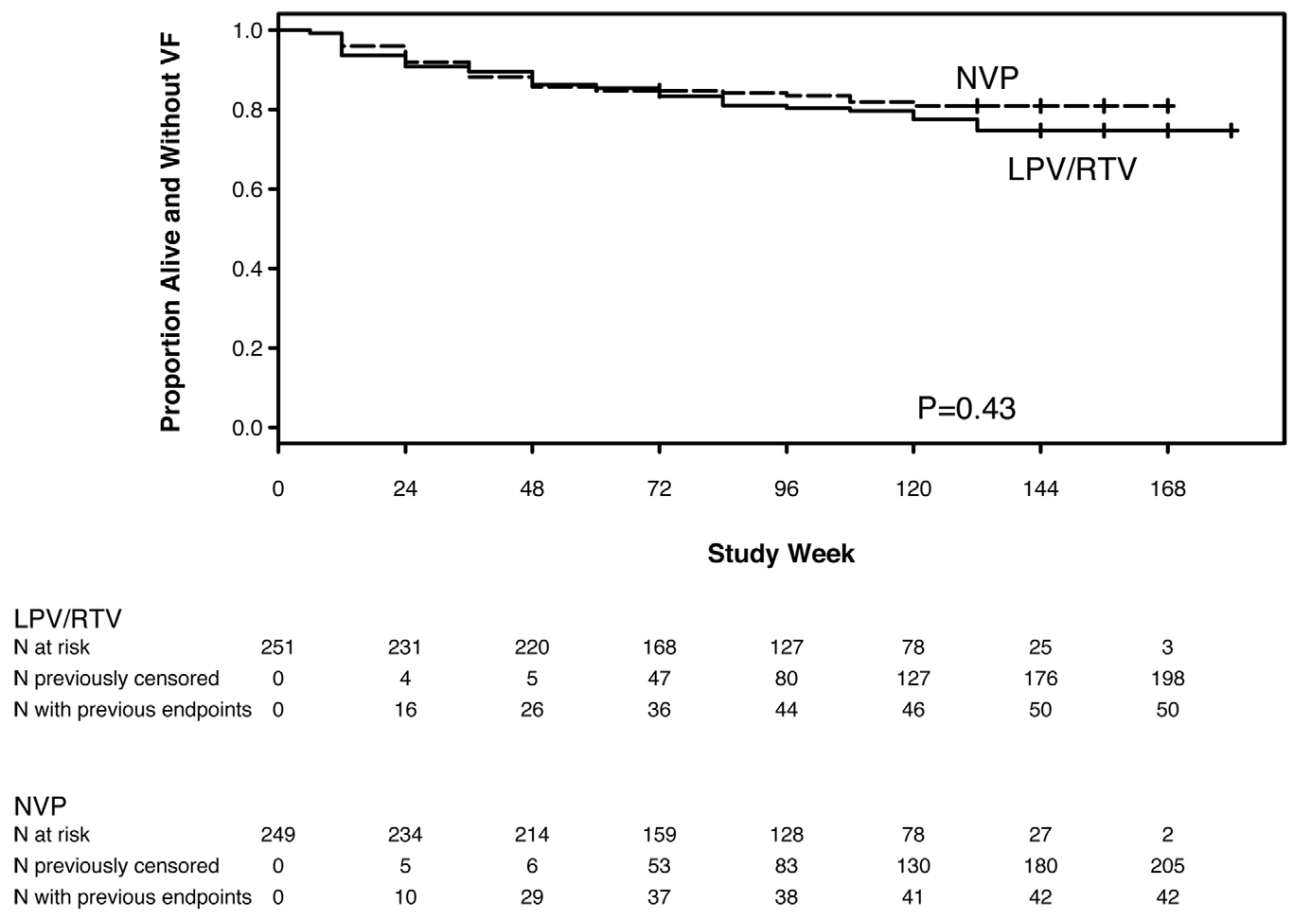
Kaplan-Meier plot of time to primary endpoint (VF or death) by randomized treatment arm.

HIV-1 RNA results were available from 99.6% of 4,223 expected time points. 92 (18%) women either experienced VF or died without prior VF, including 42 (17%) assigned to NVP and 50 (20%) to LPV/r (HR 0.85, 95% CI 0.56–1.29, *p* = 0.43) (Figure 2). This group included 37 (15%) and 43 (17%) women in the NVP and LPV/r arms, respectively, who experienced VF; and five (2%) and seven (3%) in the NVP and LPV/r arms, respectively, who died without prior VF (one additional woman died following confirmed VF). There was no significant evidence that the HR for the primary endpoint changed with increasing follow-up time (*p* = 0.58).

Based on the 95% CI for the HR (0.56–1.29), the NVP- and LPV/r-based regimens showed equivalent efficacy for the endpoint of VF or death according to the pre-specified criterion. The estimated percentage of women experiencing VF/death by week 48 was 14.3% with NVP and 13.7% with LPV/r (absolute difference = 0.6%). The associated 95% CI (−5.5% to 6.8%) was entirely within the range −10% to +10%. The corresponding results in the NVP and LPV/r arms, respectively, were 16.5% and 19.6% (difference: −3.1%; 95% CI −10.2% to 4.0%) at week 96, and 19.1% and 25.3% (difference: −6.2%; 95% CI −15.0% to 2.7%) at week 144.

The primary cause of death in the five women in the NVP arm was tuberculosis, pneumonia, acute renal failure, pulmonary embolus, and unknown (the last patient had headache and prior renal failure and pleural effusion). The death from acute renal failure (but no other deaths in the NVP arm) was deemed probably related to study treatment. Among the eight women in the LPV/r arm who died, three deaths (due to acute renal failure, severe gastroenteritis, and hepatic encephalopathy) were considered possibly related to study treatment. The cause of death in the other five women in the LPV/r arm was gastroenteritis, progressive HIV disease, central nervous system lymphoma, bacterial septicemia, and unknown in one woman.

19 (8%) of the women in the NVP arm experienced disease progression (to a higher WHO stage) or died, compared with 26 (10%) in the LPV/r arm (*p* = 0.30).

### Adjusted and Subgroup Analyses

The adjusted HRs for the primary endpoint comparing NVP- to LPV/r-based treatment in the whole study population varied between 0.83–0.86 (versus an unadjusted HR = 0.85) and the associated 95% CIs all remained within the range 0.50–2.00. Baseline HIV-1 RNA was the only tested variable that was predictive of reaching a primary endpoint, after controlling for randomized regimen (for each log_10_ copies/ml increase: HR 1.56, 95% CI 1.07–2.28).

In subgroup analyses, the only significant finding (interaction *p*<0.05) was for CD4. Specifically, among women with CD4≤100 cells/mm^3^ (the median for participants reaching a primary endpoint), 26 (27%) of 96 assigned to NVP versus 19 (21%) of 90 assigned to LPV/r experienced a primary endpoint (HR 1.36, 95% CI 0.74–2.51). In contrast, for women with baseline CD4>100 cell/mm^3^, the numbers were 16 (11%) of 153 and 31 (19%) of 161, respectively (HR 0.55, 95% CI 0.30–1.01) (*p* = 0.034 for interaction).

### VF or Death, As-Treated Analysis

In the as-treated analysis, 30 (12%) women in the NVP arm and 48 (19%) in the LPV/r arm experienced a primary endpoint (HR 0.71, 95% CI 0.45–1.13). The proportions of women in the NVP versus LPV/r arms experiencing a primary endpoint in the as-treated analysis were 12.2% versus 13.4% at 48 wk (difference: −1.2%; 95% CI −7.3% to 4.9%), 13.6% versus 18.8% at week 96 (difference: −5.2%; 95% CI −12.2 to 1.9%), and 15.5% versus 24.5% at week 144 (difference: −9.1%; 95% CI −17.8% to −0.4%).

### Discontinuation of NVP or LPV/r for Any Reason

**Figure 3:**
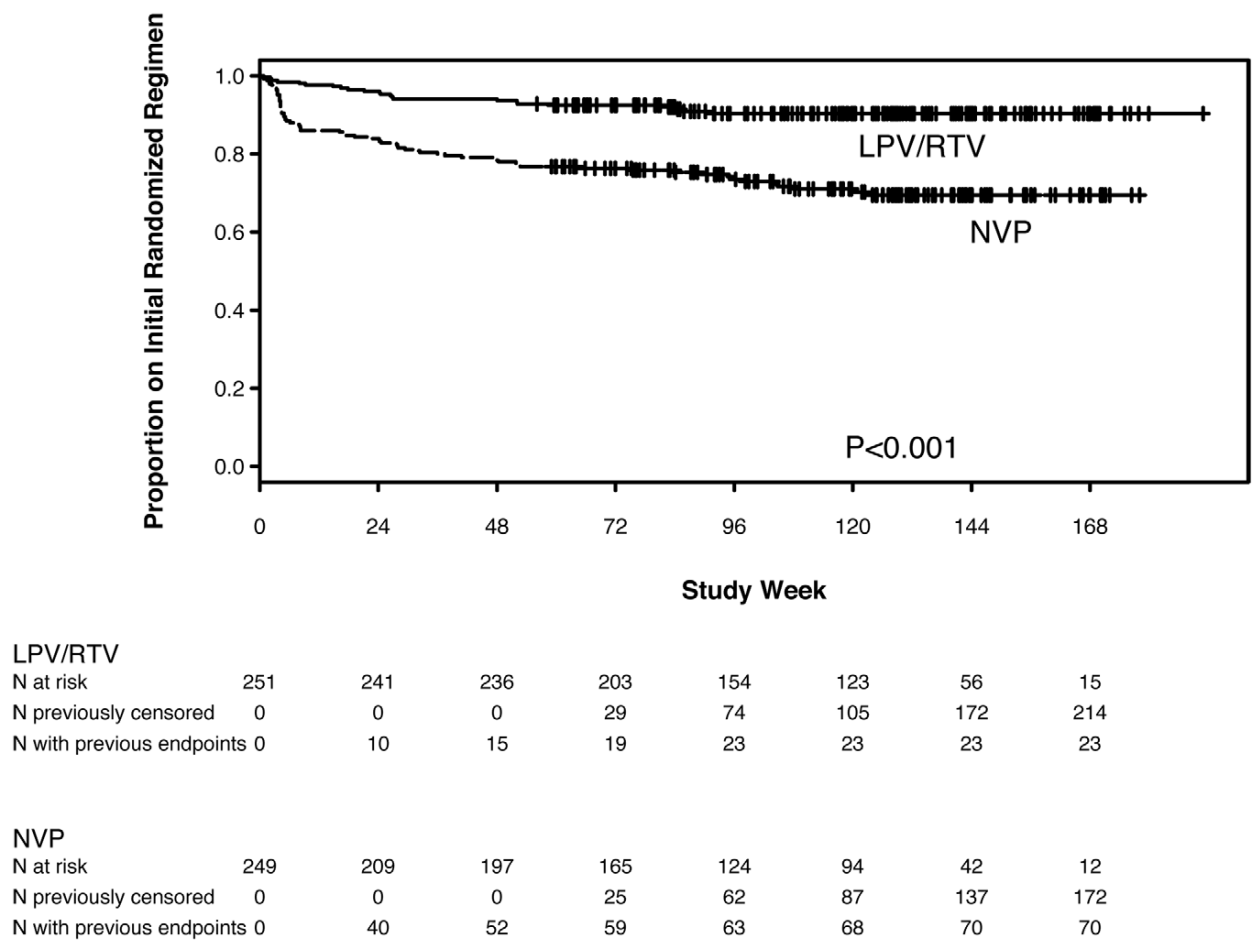
Kaplan-Meier plot of time to early discontinuation of initial randomized treatment for any reason (toxicity, intolerance, VF, death), by randomized treatment arm.

93 women discontinued their initial randomized regimen—significantly more women in the NVP (70, 28%) compared with the LPV/r (23, 9%) arm (HR 3.45, 95% CI 2.15–5.52, *p*<0.001) (Figure 3).

The reasons for discontinuation from NVP-based treatment included death (*n* = 5), AEs (*n* = 35), VF (*n* = 15), and other reasons (*n* = 15). Among the 35 women discontinuing NVP due to AEs, 20 did so following hepatic events, three following the development of both a hepatic event and a rash, and 12 following rashes (77% of these 35 women discontinued NVP during the first 8 wk on treatment). For all 35 women, the site investigators considered the association of the AE to be potentially related to study treatment.

The reasons for discontinuation from LPV/r-based treatment included death (*n* = 8), VF (*n* = 4), and other reasons (*n* = 11); no women discontinued LPV/r due to an AE.

Therefore, more women discontinued NVP (compared with LPV/r) in first line, because of either VF or to an AE, despite similar numbers of participants experiencing primary endpoints or grade 3 or higher AEs in both arms (described below). Only four of the 43 women experiencing VF in the LPV/r arm discontinued LPV/r for VF, compared with 15 of 37 women experiencing VF in the NVP arm. The decision of whether or not to switch a regimen when protocol-defined VF occurred was left to the site investigator and participant, who usually elected to intensify adherence counseling and support and continue the initial regimen, with site clinicians being less likely to switch LPV/r compared with NVP, following HIV-1 RNA>400 cp/ml occurring on treatment.

More women also discontinued NVP (compared with LPV/r) because of a clinical or laboratory abnormality. As described below, more women in the NVP arm experienced rash and/or liver test abnormality, and the protocol-mandated threshold for permanently discontinuing a study drug after the occurrence of these events was lower in the NVP arm compared with the LPV/r arm.

We also conducted a pre-specified analysis on the basis of a treatment failure composite endpoint, defined as time to the first of protocol-defined VF, death, or permanent discontinuation of initial randomized regimen. In this analysis, 80 (32%) of the 249 women randomized to NVP reached the composite endpoint compared with 54 (22%) of the 251 women randomized to LPV/r (HR 1.68, 95% CI 1.18–2.40). The occurrence of significantly more composite treatment failure endpoints among women in the NVP arm was entirely due to a higher rate of treatment discontinuation in the NVP arm (and not due to higher rates of VF or death).

### Changes in CD4 Cell Count

At least 98% of expected CD4 cell counts were obtained at each scheduled measurement time. In intent-to-treat analyses, mean change in CD4 was 183 cells/mm^3^ in each arm at week 48 (*p* = 0.99), 245 for NVP versus 279 cells/mm^3^ for LPV/r at week 96 (*p* = 0.045) and 303 for NVP versus 345 cells/mm^3^ for LPV/r at week 144 (*p* = 0.15).

### Toxicity and Diagnoses Occurring during Initial Assigned Treatment

75 (15%) of the 500 women experienced ≥grade 3 signs or symptoms through the date of the last dose of initial study treatment (Table 2): 34 (14%) in the NVP and 41 (16%) in the LPV/r arms (seven and eight women, respectively, experienced grade 4 signs/symptoms). 64 (26%) women in the NVP versus 54 (22%) in the LPV/r arm experienced ≥grade 3 laboratory abnormalities. More women in the LPV/r arm had ≥grade 3 elevation in creatinine (eight versus two), while more women in the NVP arm had ≥grade 3 elevation in liver function tests (18 versus nine). At 24, 48, and 96 wk after entry, participants in the LPV/r arm experienced significantly greater increases in total cholesterol and in low-density lipoprotein (LDL) (but smaller increases in high-density lipoprotein [HDL]) than participants in the NVP arm (while triglycerides increased in women in the LPV/r arm but decreased in women in the NVP arm) (Table 2).

**Table 2:** Signs/symptoms, laboratory abnormalities, diagnoses, and lipid results among participants on their first randomized treatment regimens.

Clinical/Laboratory Outcome	NVP Arm (*n* = 249)	LPV/r Arm (*n* = 251)	Total (*n* = 500)	Unadjusted *p*-Value
**Grade 3 or higher sign or symptom:**	**34**	**41**	**75**	
General body (pain, fatigue, weight loss, fever)	14	27	41	
Gastrointestinal (diarrhea, nausea/vomiting)	5	5	10	
Respiratory	4	6	10	
Cardiovascular	3	3	6	
Liver/hepatic	0	1	1	
Skin	9	2	11	
Neurological	7	6	13	
**Grade 3 or higher laboratory abnormality:**	**64**	**54**	**118**	
Creatinine^a^	2	8	10	
Chemistry (any abnormality)	15	18	33	
Sodium	8	14	22	
Potassium	2	1	3	
Alkaline phosphatase	3	1	4	
Liver/hepatic (any abnormality)^a^	18	9	27	
SGOT	15	6	21	
SGPT	16	7	23	
Total bilirubin	2	3	5	
Hematology (any abnormality)	37	30	67	
Platelets	1	1	2	
Hemoglobin	6	11	17	
Absolute neutrophil count	31	21	52	
**Number of participants with new diagnoses**	**150**	**164**	**314**	
**Number of participants with pregnancy on-study**	**21**	**26**	**47**	
**Change in lipids from baseline to 48 wk (mg/dl; standard error)**				
Total cholesterol	19 (±2.3)	31 (±2.2)		0.0008
HDL cholesterol	17 (±1.3)	11 (±0.9)		<0.0001
LDL cholesterol	6 (±1.8)	16 (±1.9)		0.0005
Triglycerides	−19 (±6.1)	24 (±4.3)		<0.0001

Subcolumns may not add up to total number of signs/symptoms or laboratory abnormalities, as participants can experience more than one event.

a In the NVP and LPV/r arms, respectively, three and 12 participants experienced grade 2 or higher creatinine elevation, and 34 and 19 of participants experienced grade 2 or higher liver test abnormalities.

HDL, high-density lipoprotein; LDL, low-density lipoprotein; SGOT, serum glutamic oxaloacetic transaminase; SGPT, serum glutamic pyruvic transaminase.

25 women switched from NVP or LPV/r to EFV during rifampin-containing tuberculosis treatment, including 14 assigned NVP and 11 assigned LPV/r. 47 women became pregnant during the study, including 21 assigned NVP and 26 assigned LPV/r (Table 2).

### Drug Resistance

HIV-1 drug resistance testing was attempted on samples from pre-therapy and failure time points among the 80 participants experiencing VF, with genotypes available for 69 baseline-failure pairs (Table 3). Genotypes from time of VF revealed that 13 (45%) of 29 women in NVP arm and six (15%) of 40 women in the LPV/r arm had any resistance mutation (excluding minor PI mutations). 13 (45%) women in the NVP versus three (8%) women in the LPV/r arm had ≥1 non-NRTI (NNRTI)-associated mutation at VF, and nine (31%) versus five (13%) (respectively) had NRTI-associated mutations (most often K65R and M184V). New (compared to pre-therapy) minor protease mutations were detected in five participants (two in the NVP and three in the LPV/r arms); no major PI mutations were found.

**Table 3:** Summary of drug resistance mutations at time of virologic failure.

Variable	*n* (%) NVP Arm, *n* = 37^a^	*n* (%) LPV/r Arm, *n* = 43
**Samples with VF events**	37	43
Genotype available at VF	29 (78)	40 (93)
No sample available for testing	1 (3)	0 (0)
No sequence available (unable to amplify)	7 (19)	3 (7)
**Samples with any mutation**	13 (45)	6 (15)
**Samples with NRTI-associated mutations**	9 (31)	5 (13)
K65R	8 (28)	2 (5)
K70W	1 (3)	0 (0)
M184V	9 (31)	3 (8)
Thymidine-associated mutations	2 (7)	1 (3)
**Samples with NVP or EFV mutations**	13 (45)	3 (8)
K103N	4 (14)	1 (3)
V106A	2 (7)	0 (0)
V106M	4 (14)	1 (3)
V108I	4 (14)	1 (3)
Y181C	8 (28)	0 (0)
Y181I	1 (3)	0 (0)
Y188C	1 (3)	0 (0)
G190A	2 (7)	2 (5)
**Samples with any new protease mutations**	2 (5)	3
Major protease mutations	0 (0)	0 (0)
Minor protease mutations^b^	2 (5)	3 (7)
**Samples with >1 NRTI mutation**	9	1
**Samples with >1 NNRTI mutation**	9	2
**Samples with ≥1 NRTI+≥1 NNRTI mutation**	9	2

a Percent mutation per treatment arm calculated as the number of patients with mutation divided by number of patients with genotype results available.

b New minor protease-resistance mutations detected for NVP arm were L10I/V and L63P and for LPV/r arm were K20R and K20M (data not shown).

## Discussion

Among African treatment-naïve women with CD4 count <200 cells/mm^3^, initial ART with NVP+TDF/FTC showed equivalent efficacy compared with LPV/r+TDF/FTC in intention-to-treat analysis of the primary endpoint of VF or death (83% versus 80% of women were alive and had not experienced VF after a median follow-up of more than 2 y; HR 0.85, 95% CI 0.56–1.29, which was within the pre-specified range of equivalence from 0.5 to 2.0). Equivalence was not established in the as-treated analysis, with lower rates of VF/death in the NVP arm compared with the LPV/r arm (HR 0.71, 95% CI 0.45–1.13). However, significantly more women discontinued NVP than LPV/r (28% versus 9%, *p*<0.001) including 14% versus none due to AEs. Consequently, analysis of overall regimen failure (VF, death, or discontinuation due to AEs) revealed lower rates of these combined endpoints in the LPV/r arm. The proportions of women experiencing ≥grade 3 signs, symptoms, or laboratory abnormalities did not differ significantly between arms, but total cholesterol and triglyceride increases were significantly larger in the LPV/r arm. Therefore, the higher rate of permanent discontinuation of NVP was primarily driven by protocol-mandated thresholds for permanent discontinuation of NVP compared with LPV/r, rather than higher rates of severe or life-threatening toxicities in the NVP arm. Overall, these data support the WHO recommendation of NVP/TDF/FTC as an initial affordable and effective HIV treatment regimen in RLS, and provide reassurance regarding the efficacy of this regimen. However, these results also underscore the importance of early toxicity monitoring with NVP-based regimens. The treatment failure (due to VF or treatment discontinuation) observed in both arms also highlights the importance of access to effective second treatment options, as well as consideration of other effective, better-tolerated first-line regimens (including among women of reproductive potential).

The overall and relative efficacy and toxicity of NVP-based versus PI-based ART are relevant in RLS. Globally, NVP is the most frequently used antiretroviral in combination with two NRTIs, due primarily to its low cost, lack of teratogenicity, and heat stability; and regimens including TDF+NVP (with either 3TC or FTC) are recommended by the WHO and increasingly used [1]. However, prior small studies of first-line ART composed of NVP+TDF/FTC demonstrated rather high rates of early VF on this regimen, raising concern about its use [2],[3]. If LPV/r were found to be significantly more (or more durably) potent or less toxic than NVP in first-line treatment, then it could potentially represent a cost-effective choice for initial regimen in RLS (as reported in a previous cost-effectiveness analysis comparing these regimens among women with prior sdNVP exposure [4]), although this has yet to be modeled in ARV-naive patients. To our knowledge, this is the first direct comparison of the efficacy of NVP- and LPV/r-based ART, and one of only a small number of studies to compare the efficacy of ART using NVP versus any boosted PI [7],[8]. This is also one of the largest randomized treatment trials to be conducted in HIV-infected women, who may experience different toxicities or response compared with men, but who are often underrepresented in trials. In one previous study (“ARTEN” [8]), 569 treatment-naive patients were randomized to initiate NVP or atazanavir/ritonavir, each in combination with Truvada. Similar proportions achieved virologic suppression with both treatments, although more patients stopped treatment due to AEs in the NVP (14%) than the atazanavir (4%) arm [8]. These findings are qualitatively similar to the OCTANE Trial 2 results, but are in contrast to those of an observational European study, in which patients were more likely to stop first-line NVP because of treatment failure but less likely to do so because of toxicity or patient/provider choice, compared with EFV or LPV/r [7].

Genotype analysis in our study of samples at the time of failure revealed NNRTI- or NRTI-resistance mutations in nearly one-half of women in the NVP arm. This frequency of resistance is lower than that reported in other studies of failure of initial therapy [9]–[11], and may be related to more rapid regimen switch (in the context of careful HIV-1 RNA monitoring) or to premature discontinuation of therapy for suspected toxicity. In the LPV/r arm, major PI resistance mutations were not detected at failure (similar to findings from other studies of LPV/r [9],[12]), and NRTI-associated mutations were less frequent (13%) than in the NVP arm (31%). The occurrence of the K65R mutation in 28% of participants failing NVP+TDF/FTC is notable. These resistance findings have implications for selection of first-line and subsequent ART regimens to optimize long-term clinical outcomes and reduce spread of drug resistance.

Strengths of our study included its randomized nature and very high visit and data completeness. Limitations include the possibility that some participants may have had previous sdNVP exposure. However, the accuracy of our sdNVP exposure ascertainment is supported by a low rate (0.8%) of baseline NVP resistance, and by results from trial 1 (which demonstrated higher rates of VF/death with NVP versus LPV/r treatment among women with prior sdNVP exposure [5]). Subgroup analyses need to be interpreted with caution given the number of subgroup analyses considered; the one difference (between women with higher versus lower CD4 counts) identified would not be significant with adjustment for multiple comparisons and so could plausibly be a chance finding. We could not compare long-term morbidity and mortality between regimens because of the study duration. This was an open-label trial, and the lack of blinding constitutes another potential limitation. Finally, patient outcomes in a closely monitored clinical trial setting, including ramifications of toxicity, may be better than those expected in a routine treatment setting in RLS.

We conclude that in antiretroviral-naïve women with CD4 count <200 cells/mm^3^, initial ART with LPV/r/TDF/FTC and with NVP+TDF/FTC is equivalent in achieving and maintaining virologic suppression and preventing mortality, but that treatment cessation due to toxicity concerns, and drug resistance at the time of VF, are higher with NVP-based ART. Our findings suggest that NVP (with careful early toxicity monitoring) remains an acceptable choice for first-line ART in RLS, until better tolerated and potentially more efficacious regimens become accessible.

## Supporting Information

Text S1 Trial protocol.
(DOC)

Text S2 CONSORT checklist.
(DOC)

## Abbreviations

ART: antiretroviral treatment
FTC: emtricitabine
HR: hazard ratio
LPV/r: lopinavir/ritonavir
NNRTI: non-nucleoside analog reverse transcriptase inhibitor
NRTI: nucleoside analog reverse transcriptase inhibitor
NVP: nevirapine
PI: protease inhibitor
RLS: resource-limited settings
sdNVP: single dose nevirapine
TDF: tenofovir
VF: virologic failure
WHO: World Health Organization
ZDV: zidovudine
