# Supplementary material for: Nevirapine- Versus Lopinavir/Ritonavir-Based Initial Therapy for HIV-1 Infection among Women in Africa: A Randomized Trial
Source: PLoS Med. 2012 Jun 12;9(6):e1001236. doi: 10.1371/journal.pmed.1001236 (PMC3373629; doi:10.1371/journal.pmed.1001236)
Supplement: Text S1 — Trial protocol. (DOC) [file pmed.1001236.s001.doc]

**A5208/OCTANE**

**Optimal Combination Therapy After Nevirapine Exposure**

**A Collaborative, Multicenter Trial Between the AIDS Clinical Trials Group (ACTG) and the Division of AIDS (DAIDS)-Sponsored Networks**

**Sponsored by:**

**The National Institute of Allergy**

**and Infectious Diseases**

**Pharmaceutical Support Provided by:**

**Abbott Laboratories**

**Boehringer Ingelheim Pharmaceuticals**

**Bristol-Myers Squibb**

**Gilead Sciences**

**GlaxoSmithKline**

**Merck & Co.**

**The DAIDS Therapeutic Research Program Jeffrey Nadler, M.D.**

**Optimization of Antiretroviral Therapy**

**Committee: Roy Gulick, M.D., Chair**

**Protocol Chair: Shahin Lockman, M.D., M.Sc.**

**Protocol Co-Chair: Frederick Sawe, M.D.**

**Protocol Vice Chairs: Judith Currier, M.D., M.Sc.**

**James McIntyre, M.D.**

DAIDS Clinical Representative: Beverly Alston-Smith, M.D.

**Clinical Trials Specialist: Evelyn Hogg, B.A.**

**FINAL Version 4.0**

**April 13, 2009**

CONTENTS

Page

SITES PARTICIPATING IN THE STUDY [5](#__RefHeading___Toc226540903)

PROTOCOL TEAM ROSTER [6](#__RefHeading___Toc226540904)

STUDY MANAGEMENT [14](#__RefHeading___Toc226540905)

GLOSSARY [17](#__RefHeading___Toc226540906)

SCHEMA [19](#__RefHeading___Toc226540907)

1.0 HYPOTHESIS AND STUDY OBJECTIVES [22](#__RefHeading___Toc226540908)

1.1 Hypothesis [22](#__RefHeading___Toc226540909)

1.2 Primary Objectives [22](#__RefHeading___Toc226540910)

1.3 Secondary Objectives [23](#__RefHeading___Toc226540911)

2.0 INTRODUCTION [24](#__RefHeading___Toc226540912)

2.1 Background [24](#__RefHeading___Toc226540913)

2.2 Antiretrovirals [30](#__RefHeading___Toc226540914)

2.3 Rationale [52](#__RefHeading___Toc226540915)

3.0 STUDY DESIGN [59](#__RefHeading___Toc226540916)

4.0 SELECTION AND ENROLLMENT OF PARTICIPANTS [63](#__RefHeading___Toc226540917)

4.1 Inclusion Criteria for Step 1 [63](#__RefHeading___Toc226540918)

4.2 Exclusion Criteria for Step 1 [65](#__RefHeading___Toc226540919)

4.3 Inclusion Criteria for Step 2 [67](#__RefHeading___Toc226540920)

4.4 Exclusion Criterion for Step 2 [67](#__RefHeading___Toc226540921)

**4.5 Inclusion Criteria for Step 3** [67](#__RefHeading___Toc226540922)

**4.6 Exclusion Criteria for Step 3** [67](#__RefHeading___Toc226540923)

**4.7** Study Enrollment Procedures [67](#__RefHeading___Toc226540924)

**4.8** Coenrollment Guidelines [68](#__RefHeading___Toc226540925)

5.0 STUDY TREATMENT [68](#__RefHeading___Toc226540926)

5.1 Regimens, Substitutions, Administration, and Duration [69](#__RefHeading___Toc226540927)

5.2 Study Product Formulation and Preparation [76](#__RefHeading___Toc226540928)

5.3 Study Product Supply, Distribution, and Pharmacy [77](#__RefHeading___Toc226540929)

5.4 Concomitant Medications **(Steps 1 and 2)** [77](#__RefHeading___Toc226540930)

5.5 Adherence Assessment [84](#__RefHeading___Toc226540931)

**5.6 Adherence Interview (Step 3)** [84](#__RefHeading___Toc226540932)

6.0 CLINICAL AND LABORATORY EVALUATIONS [85](#__RefHeading___Toc226540933)

6.1 Schedule of Events—Trial 1 and Trial 2**, Steps 1 and 2** [85](#__RefHeading___Toc226540934)

**6.1 Schedule of Events - Step 3** [86](#__RefHeading___Toc226540935)

6.2 Timing of Evaluations [87](#__RefHeading___Toc226540936)

6.3 Special Instructions and Definitions of Evaluations [91](#__RefHeading___Toc226540937)

7.0 TOXICITY MANAGEMENT [102](#__RefHeading___Toc226540938)

7.1 Dosage Modification Instructions [102](#__RefHeading___Toc226540939)

7.2 Guidelines for Most Grade 1 or 2 Toxicities [103](#__RefHeading___Toc226540940)

7.3 Guidelines for Most Grade 3 Toxicities [103](#__RefHeading___Toc226540941)

7.4 Guidelines for Most Grade 4 Toxicities [103](#__RefHeading___Toc226540942)

7.5 Management of Laboratory Abnormalities and Clinical Syndromes [104](#__RefHeading___Toc226540943)

7.6 Management of Immune Reconstitution Inflammatory Syndromes [117](#__RefHeading___Toc226540944)

8.0 CRITERIA FOR STUDY OR TREATMENT DISCONTINUATION [118](#__RefHeading___Toc226540945)

8.1 Criteria for Treatment Discontinuation **(Steps 1 and 2 only)** [118](#__RefHeading___Toc226540946)

8.2 Criteria for Study Discontinuation **(Steps 1, 2, and 3)** [118](#__RefHeading___Toc226540947)

9.0 STATISTICAL CONSIDERATIONS [118](#__RefHeading___Toc226540948)

9.1 General Design Issues **(Steps 1 and 2)** [118](#__RefHeading___Toc226540949)

9.2 Endpoints [120](#__RefHeading___Toc226540950)

9.3 Randomization and Stratification **(Steps 1 and 2)** [122](#__RefHeading___Toc226540951)

9.4 Sample Size and Accrual **(Steps 1 and 2)** [122](#__RefHeading___Toc226540952)

9.5 Monitoring **(Steps 1 and 2)** [128](#__RefHeading___Toc226540953)

9.6 Analyses **(Steps 1 and 2)** [131](#__RefHeading___Toc226540954)

**9.7** **Statistical Considerations for Step 3** [131](#__RefHeading___Toc226540955)

10.0 DATA COLLECTION AND MONITORING AND ADVERSE EXPERIENCE REPORTING [133](#__RefHeading___Toc226540956)

10.1 Records to Be Kept [133](#__RefHeading___Toc226540957)

10.2 Role of Data Management [133](#__RefHeading___Toc226540958)

10.3 Clinical Site Monitoring and Record Availability [133](#__RefHeading___Toc226540959)

10.4 Expedited Serious Adverse Event (EAE) Reporting [134](#__RefHeading___Toc226540960)

11.0 HUMAN PARTICIPANTS [135](#__RefHeading___Toc226540961)

11.1 Institutional Review Board (IRB) Review and Informed Consent [135](#__RefHeading___Toc226540962)

11.2 Participant Confidentiality [135](#__RefHeading___Toc226540963)

11.3 Study Discontinuation [135](#__RefHeading___Toc226540964)

12.0 PUBLICATION OF RESEARCH FINDINGS [136](#__RefHeading___Toc226540965)

13.0 BIOHAZARD CONTAINMENT [136](#__RefHeading___Toc226540966)

14.0 REFERENCES [137](#__RefHeading___Toc226540967)

APPENDIX I: SAMPLE INFORMED CONSENT **(VERSION 3.0)**

APPENDIX II: PREGNANCY SAMPLE INFORMED CONSENT (VERSION 3.0)

**APPENDIX III:** **ADDENDUM TO SAMPLE INFORMED CONSENT (FOR STEP 3, VERSION 4.0)**

###### SITES PARTICIPATING IN THE STUDY

Participation in A5208/OCTANE will be limited to sites outside the U.S. that will be selected by the DAIDS. The names of the selected sites will be posted on the A5208/OCTANE protocol-specific Web page.

###### PROTOCOL TEAM ROSTER

Protocol Chair

Shahin Lockman, M.D., M.Sc.

Brigham and Women's Hospital and Department of Immunology and Infectious Diseases

Infectious Diseases Division

Harvard School of Public Health

FXB 401, 655 Huntington Avenue

Boston, MA 02115

Phone: (617) 432-4767

FAX: (617) 739-8348

Email: [slockman@hsph.harvard.edu](mailto:slockman@hsph.harvard.edu)

**Protocol Co-Chair**

Frederick Sawe, M.D.

The Walter Reed Project/WRAIR

Hospital Road

P.O. Box 1357

Kericho, 20200

KENYA

Mobile phone: 254-722-725-794

E-mail: [fsawe@wrp-kch.org](mailto:fsawe@wrp-kch.org)

Vice Chairs

Judith Silverstein Currier, M.D., M.Sc.

University of California, Los Angeles

Center for AIDS Research and Education

10833 Le Conte Avenue

Room-BH-412, CHS

Los Angeles, CA 90095-1793

Phone: (310) 206-6414

FAX: (310) 206-3311

E-mail: [jscurrier@mednet.ucla.edu](../jscurrier@mednet.ucla.edu)

Vice Chairs (Cont’d)

James Alasdair McIntyre, M.D.

Department of Obstetrics & Gynecology

University of the Witwatersrand

Chris Hani Baragwanath Hospital

P.O. Bertsham

Johannesburg 2103

SOUTH AFRICA

Phone: 271 198 99703

FAX: 271 198 99762

E-mail: [mcintyre@pixie.co.za](mailto:mcintyre@pixie.co.za)

DAIDS Clinical Representative

Beverly L. Alston-Smith, M.D.

CCRB, TRP, DAIDS, NIAID, NIH

Room 5109

6700-B Rockledge Drive MSC 7624

Bethesda, MD 20892-7624

Phone: (301) 435-3773

FAX: (301) 402-3171

E-mail:[**balston@niaid.nih.gov**](../balston@niaid.nih.gov)

Clinical Trials Specialist

Evelyn Hogg, B.A.

Social & Scientific Systems, Inc.

ACTG Operations Center

8757 Georgia Avenue, 12^th^ Floor

Silver Spring, MD 20910-3714

Phone: (301) 628-3337

FAX: (301) 628-3302

E-mail: [ehogg@s-3.com](mailto:ehogg@s-3.com)

Statisticians

Michael Hughes, Ph.D.

Statistical & Data Analysis Center

Harvard School of Public Health

Building 2, Room 439A

655 Huntington Avenue

Boston, MA 02115-6017

Phone: (617) 432-3161

FAX: (617) 432-2832

E-mail: [mhughes@sdac.harvard.edu](mailto:mhughes@sdac.harvard.edu)

Statisticians (Cont’d)

Yu (Evelyn) Zheng, M.S.

Statistical & Data Analysis Center

Harvard School of Public Health

FXB 512, 655 Huntington Avenue

Boston, MA 02115

Phone: (617) 432-1161

FAX: (617) 432-3163

E-mail: [ezheng@sdac.harvard.edu](mailto:ezheng@sdac.harvard.edu)

Data Manager**s**

**Apsara Nair, M.S.**

**Frontier Science & Technology Research Foundation**

**4033 Maple Road**

**Amherst, NY 14226**

**Phone: (716) 834-0900x7293**

**FAX: (716) 834-8432**

**E-mail:** [**nair@fstrf.org**](mailto:nair@fstrf.org)

**Ann Walawander, M.A.**

**Frontier Science & Technology Research Foundation**

**4033 Maple Road**

**Amherst, NY 14226-1056**

**Phone: (716) 834-0900x7290**

**FAX: (716) 834-8432**

**E-mail:** [**walawander.ann@fstrf.org**](mailto:walawander.ann@fstrf.org)

DAIDS Pharmacist

**Lynette Purdue, Pharm.D.
NIAID/DAIDS/NIH
Pharmaceutical Affairs Branch
6700B Rockledge Drive, Room 4107
Bethesda, MD 20892-7620
Phone: (301) 435-3744
FAX: (301) 402-1506
E-mail:** [**lpurdue@niaid.nih.gov**](mailto:lpurdue@niaid.nih.gov)

Virologists

Susan H. Eshleman, M.D., PH.D.

Department of Pathology

Johns Hopkins Medical Institution

Ross Building 646

720 Rutland Avenue

Baltimore, MD 21205

Phone: (410) 614-4734

FAX: (410) 614-3548

E-mail: [seshlem@jhmi.edu](../seshlem@jhmi.edu)

John W. Mellors, M.D.

University of Pittsburgh Medical Center

Graduate School of Public Health

Scaife Hall, Suite 818

3550 Terrace Street

Pittsburgh, PA 15261

Phone: (412) 383-7963

FAX: (412) 383-7982

E-mail: [mellors@msx.dept-med.pitt.edu](../mellors@msx.dept-med.pitt.edu)

Pharmacologists

Francesca Aweeka, Pharm.D.

Drug Research Unit

Department of Clinical Pharmacy

University of California, San Francisco

UCSF Box 0622

Third Ave & Parnassus

San Francisco, CA 94143-0622

Phone: (415) 476-0339

FAX: (415) 476-0307

E-mail: [faweeka@sfghsom.ucsf.edu](../faweeka@sfghsom.ucsf.edu)

Charles Chiedza Maponga, Pharm.D.

University of Zimbabwe, College of Health Sciences

DaTIS-UZ Medical School

Mazoe Street (P.O. Box A178)

Harare

ZIMBABWE

Phone: 263 479 1520

FAX: 263 479 0233

E-mail: [cmaponga@medsch.uz.ac.zw](../cmaponga@medsch.uz.ac.zw)

**Site** Investigators

Farida Amod, M.B., CHB, FCPath (micro), FCP

University of Natal

Nelson R. Mandela School of Medicine

Department of Medicine

Private Bag 7

Congella, Durban 4013

SOUTH AFRICA

Phone: 273 126 04637

Mobile Phone: 082 449 5933

FAX: 273 126 04411

E-mail: [amodf1@nu.ac.za](mailto:amodf1@nu.ac.za)

**Aida Tesfamaraim Asmelash, M.D., M.P.H.**

**Molepolole Prevention/Treatment Trials CRS**

**BHP**

**North Ring Road**

**Plot Number 1836**

**P/Bag 320**

**Gaborone 320**

**BOTSWANA**

**Phone: 26 73 97 5776**

**E-mail:** [**aida.asmelash@gmail.com**](mailto:aida.asmelash@gmail.com)

Thomas B. Campbell, M.D.

Associate Professor of Medicine

University of Colorado Health Sciences Center

Infectious Diseases

Box B-168

4200 East Ninth Avenue

Denver, CO 80111

Phone: (303) 315-8311

FAX: (303) 315-8681

E-mail: [**thomas.campbell@ucdenver.edu**](mailto:thomas.campbell@ucdenver.edu)

**Site** Investigators (Cont’d)

Monica Carten, M.D.

University of Colorado Health Sciences Center

Colorado Adult AIDS Clinical Trials Unit

4200 E. Ninth Avenue

Denver, CO 80262

Phone: (303) 315-1540

FAX: (303) 315-1469

E-mail: [monica.carten@uchsc.edu](mailto:monica.carten@uchsc.edu)

Tsungai Chipato, M.D.

UZ-UCSF Collaborative Research Programme

15 Phillips Avenue

Belgravia

Harare, CA

ZIMBABWE

Phone: 263 430 8840

FAX: 263 470 4897

E-mail: [tchipato@zol.co.zw](mailto:tchipato@zol.co.zw)

Francesca Conradie, M.B., B.Ch.

University of Witwatersrand

Clinical HIV Research Unit

Postnet Suite 176

Private Bag X2600

Houghton

Johannesburg, Gauteng 2041

SOUTH AFRICA

Phone: 27 11 2768814

FAX: 27 11 4822130

E-mail: [fconradie@witshealth.co.za](mailto:fconradie@witshealth.co.za)

**Site** Investigators (Cont’d)

**Elizabeth Dangaiso, B.Sc.N.**

**University of Zimbabwe, Harare**

**UZCRC Corner Josiah Tongogara Ave/ Mazowe St Harare**

**P.O. Box A1578**

**Avondale, Harare 263**

**ZIMBABWE**

**Phone: (263) 47-01717**

**FAX: (263) 47-01986**

**E-mail:** [**edangaiso@uzcrc.co.zw**](mailto:edangaiso@uzcrc.co.zw)

Betty Dong, Pharm.D.

University of California, San Francisco

521 Parnassus Avenue, C-152

San Francisco, CA 94149-0622

Phone: (415) 476-1972

FAX: (415) 476-6632

E-mail: [bjdong@itsa.ucsf.edu](mailto:bjdong@itsa.ucsf.edu)

Scott M. Hammer, M.D.

Columbia University College of Physicians and Surgeons

622 West 168th Street

PH-8W, Suite 876

New York, NY 10032

Phone: (212) 305-7185

FAX: (212) 305-7290

E-mail: [smh48@columbia.edu](mailto:smh48@columbia.edu)

Jane Hitti, M.D., M.P.H.

University of Washington Medical Center

Perinatal Medicine

Department of Obstetrics/Gynecology

Box 356460

Seattle, WA 98195

Phone: (206) 543-9867

FAX: (206) 616-9479

E-mail: [jhitti@u.washington.edu](mailto:jhitti@u.washington.edu)

**Site** Investigators (Cont’d)

**Mina Christine Hosseinipour, M.D.**

**University of North Carolina Project**

**Kamuza Central Hospital**

**Tidziwe Centre**

**100 Mzimba Road**

**Private Bag A-104**

**Lilongwe**

**MALAWI**

**Office: 011 265 1 758 938**

**FAX: 011 265 175 5954**

**E-mail:** [**minach@med.unc.edu**](mailto:minach@med.unc.edu)

Cissy Kityo Mutuluuza, M.B., CHB, M.Sc Joint Clinical Research Centre

Ring Road, Butikiro House

P.O. Box 10005

Kampala

UGANDA

Phone: 256 41 270622/270283, x126

Mobile phone: 256 075 769 168

FAX: (256) 413-42632

E-mail: [ckityo@jcrc.co.ug](mailto:ckityo@jcrc.co.ug)

U.S Contact Information:

Phone: (410) 234-6630

E-mail: [ckityo@yahoo.com](mailto:ckityo@yahoo.com)

Daniel Kuritzkes, M.D.

Brigham and Women's Hospital

Harvard Medical School

65 Landsdowne Street

Room 449

Cambridge, MA 02139

Phone: (617) 768-8371

FAX: (617) 768-8738

E-mail: [dkuritzkes@partners.org](mailto:dkuritzkes@partners.org)

**Site** Investigators (Cont’d)

Mary Marovich, M.D., DTM&H

Walter Reed Army Institute of Research-U.S. Military HIV Research Program

13 Taft Ct., Suite 200

Rockville, MD 20850

Phone: (301) 251-8337

FAX: (301) 762-4177

E-mail: [mmarovich@hivresearch.org](mailto:mmarovich@hivresearch.org)

Lerato Mohapi, M.D.

Prenatal HIV Research Unit

Chris Hani Baragwaneth Hospital

P.O. Bertsham

Johannesburg 2013

SOUTH AFRICA

Phone: 27 11 989 9835

Mobile phone: 27 082 332 7170

FAX: 27 11 989 9762

E-mail: [mohapil@hivsa.com](mailto:mohapil@hivsa.com)

Peter Ndimbirwe Mugyenyi, M.D.

Joint Clinical Research Center

Butikiro House

Plot 893 Ring Road

P.O. Box 10005

Kampala 256

UGANDA

Phone: 256 41 342521

FAX: 256 41 342632

Pager (Numeric): 256 75 770622

E-mail: [pmugyenyi@jcrc.co.ug](mailto:pmugyenyi@jcrc.co.ug)

Michael Switow Saag, M.D.

University of Alabama at Birmingham

AIDS Outpatient (1917) Clinic

908 South 20th Street, CCB #142

Birmingham, AL 35294-2050

Phone: (205) 934-7349

FAX: (205) 975-6120

E-mail: [msaag@uab.edu](mailto:msaag@uab.edu)

**Site** Investigators (Cont’d)

Robert Salata, M.D.

Division of Infectious Diseases

Department of Medicine

Case Western Reserve University

University Hospitals of Cleveland

11100 Euclid Avenue

Cleveland, OH 44106-5083

Phone: (216) 368-4761

FAX: (216) 844-1632

E-mail: [ras7@po.cwru.edu](mailto:ras7@po.cwru.edu)

Ian M. Sanne, M.D., FCP (medicine), DTM&H

Wits Health Consortium

Helen Joseph Hospital

Perth Road Westdene

Johannesburg 2193

SOUTH AFRICA

Phone: 27 11 276 8801

FAX: 27 11 482 3120

E-mail: [isanne@witshealth.co.za](mailto:isanne@witshealth.co.za)

Robert T. Schooley, M.D.

University of California, San Diego

Stein Research Building

Room 401

Mail Code 0665

9500 Gilman Drive

La Jolla, CA 92023-0665

Phone: (858) 822-0216

FAX: (858) 822-5362

E-mail: [rschooley@ucsd.edu](mailto:rschooley@ucsd.edu)

Douglas Shaffer, M.D., M.H.S.

The Walter Reed Project/WRAIR

Hospital Road

P.O. Box 1357

Kericho, 20200

KENYA

Mobile phone: 254-734-527-545

E-mail: [dshaffer@wrp-kch.org](mailto:dshaffer@wrp-kch.org)

**Site** Investigators (Cont’d)

Abraham, Siika, MMed

Department of Internal Medicine

MOI University Faculty of Health Sciences

1st Floor, MTRH Bldg, Nandi Road

P. O. Box 4604 300100

Eldoret

KENYA

Phone: 011-254-53-822156

Cell phone: 011 254 722 280785

E-mail: [amsiika@africaonline.co.ke](mailto:amsiika@africaonline.co.ke)

Elizabeth Stringer, M.D.

University of Alabama at Birmingham

Center for Infectious Disease Research in Zambia (CIDRZ)

Plot 5977 Benakale Road

P.O. Box 34681

Northmead, Lusaka

ZAMBIA

Phone: 260-1-293-661, x133

Cell phone: 26-096-860-602

FAX: 260-1-293-766, x140

E-mail: [eli@uab.edu](mailto:eli@uab.edu)

Heather Watts, M.D.

Pediatric, Adolescent, and Maternal AIDS Branch/CRMC

National Institute of Child Health & Human Development

6100 Executive Boulevard, Room 4B11G

Bethesda, MD 20892

Phone: (301) 496-7339

FAX: (301) 496-8678

E-mail: [hw59i@nih.gov](mailto:hw59i@nih.gov)

**Site** Investigators (Cont’d)

Kara Wools-Kaloustian, M.D.

Indiana University School of Medicine

Division of Infectious Diseases

Wishard Memorial Hospital Room OPW-430

1001 West 10th Street

Indianapolis, IN 46202

Phone: (317) 630-6119

E-mail: [kwools@iupui.edu](mailto:kwools@iupui.edu)

KENYA Contact Information:

MOI University

Health Sciences

P.O. Box 4606

Eldoret

KENYA

Phone: 254-734-893871 (from US)

0734-893871 (within Kenya)

E-mail: [iuresearch@africaonline.co.ke](mailto:iuresearch@africaonline.co.ke)

Field Representative**s**

**Cheryl J. Marcus, R.N., B.S.N.**

**UNC AIDS CRS**

**130 Mason Farm Road**

**Suite 2100, Bioinformatics Building**

**Chapel Hill, NC 27514**

**Phone: (919) 843-8761**

**FAX: (919) 966-8928**

**E-mail:** [**cjm@med.unc.edu**](mailto:cjm@med.unc.edu)

**Beverly Putnam, R.N., A.N.P.**

**University of Colorado Health Science Center**

**Academic Office 1, MS 8205**

**12631 East 17th Avenue**

**P.O. Box 6511**

**Aurora, CO 80045**

**Phone: (303) 724-0762**

**FAX:(303) 724-0802**

**E-mail:** [**beverly.putnam@uchsc.edu**](mailto:beverly.putnam@uchsc.edu)

Laboratory Technologist

Robin DiFrancesco, B.S., M.T., (ASCP), M.B.A.

Laboratory for Antiviral Research

Department of Pharmacy Practice

SUNY at Buffalo

237 Cooke Hall

Buffalo, NY 14260

Phone: (716) 645-3635, x233

FAX: (716) 645-2001

E-mail: [rda@acsu.buffalo.edu](mailto:rda@acsu.buffalo.edu)

**CSS** Representative

**Peter Ndhleni Ziba**

**c/o Mr. Kennedy Phiri**

**P.O. Box 49 Unza**

**Lusaka**

**ZAMBIA**

**Phone: 011 260 977 876 391**

**E-mail:** [**petenziba@yahoo.com**](mailto:petenziba@yahoo.com)

**Consultant**

William C. Holmes, M.D., M.S.C.E.

University of Pennsylvania School of Medicine

Center for Clinical Epidemiology & Biostatistics

733 Blockley Hall

423 Guardian Drive

Philadelphia, PA 19104-6021

Phone: (215) 898-8188

FAX: (215) 573-2346

E-mail: [holmeswc@mail.med.upenn.edu](mailto:holmeswc@mail.med.upenn.edu)

Industry Representatives

**Kristy Grimm, Pharm.D.**

**Bristol-Myers Squibb, Virology**

**777 Scudders Mill Road**

**Mailstop P11-14**

**Plainsboro, NJ 08536**

**Phone: (609) 897-3544**

**FAX: (609) 897-6068**

**E-mail:** [**kristy.grimm@bms.com**](mailto:kristy.grimm@bms.com)

Industry Representatives (Cont’d)

**Lauren E. Petrella**

**Boehringer Ingelheim Pharmaceuticals, Inc.**

**900 Ridgebury Road**

**P.O. Box 368**

**Ridgefield, CT 06877**

**Phone: (203) 778-7320**

**E-mail:** [**lauren.petrella@boehringer-ingelheim.com**](mailto:lauren.petrella@boehringer-ingelheim.com)

Navdeep **Thoofer**, Ph.D.

GlaxoSmithKline PLC

Infectious Diseases, MDC

891-995 Greenford Ave.,

Greenford

Middlesex UB6 0HE

UNITED KINGDOM

Phone: 440 208-9964513

FAX: 440 208-9664088

E-mail: [**Navdeep.K.Thoofer@gsk.com**](mailto:Navdeep.K.Thoofer@gsk.com)

Sibtain Rahim, M.D., C.M.
Abbott Laboratories
465 West 23^rd^ Street, Apt. 6E
New York, NY 10011

Phone/FAX: (212) 245-5362
Mobile Phone: (847) 224-1195

E-mail: [sibtain.rahim@abbott.com](mailto:sibtain.rahim@abbott.com)

James F. Rooney, M.D.

Clinical Affairs

Gilead Sciences

333 Lakeside Drive

Foster City, CA 94404

Phone: (650) 522-5708

FAX: (650) 522-5854

E-mail: [jim_rooney@gilead.com](mailto:jim_rooney@gilead.com)

Industry Representatives (Cont’d)

Audrey L. Shaw, Ph.D.

Gilead Sciences Inc.

Clinical Research/Medical Affairs

4 University Place

4611 University Drive

Durham, NC 27707

Phone: (919) 294-7611

FAX: (919) 294-7236

E-mail: [audrey.shaw@gilead.com](mailto:audrey.shaw@gilead.com)

Laboratory Data Coordinator

**Jimi Tutko, B.S.**

**Frontier Science & Technology Research Foundation**

**4033 Maple Road**

**Amherst, NY 14226**

**Phone: (716) 834-0900x7382**

**FAX: (716) 833-0655**

**E-mail:** [**tutko@fstrf.org**](mailto:tutko@fstrf.org)

###### STUDY MANAGEMENT

Contacting the Team

All messages regarding **this study** should be sent to the A5208/OCTANE Clinical Management Committee via e-mail to [actg.cmca5208@fstrf.org](mailto:actg.cmca5208@fstrf.org).

The appropriate team member will respond via e-mail with a "cc" to the team. A response should generally be received within 24 hours (Monday-Friday).

Protocol E-mail Group

Sites registering to this study must contact the Computer Support Group at the Data Management Center **(DMC)** via e-mail ([aactg.support@fstrf.org](../ProtShell.doc)) to have the relevant personnel at the site added to the actg.protA5208 e-mail group as soon as possible. Inclusion in the protocol e-mail group will ensure that sites receive important information about the study during its implementation and conduct.

Clinical Management

For questions concerning clinical medical management, including entry criteria, toxicity management, concomitant medications, co-enrollment, virologic failure, and/or regimen switches, contact the clinical management committee (CMC).

- Send an e-mail message to [actg.cmca5208@fstrf.org](mailto:actg.cmca5208@fstrf.org)
- Include the study number (A5208), patient identification number (PID), and a brief relevant history.

Virologic or Pharmacologic Tests

For questions specifically related to virologic or pharmacologic laboratory tests, contact the protocol virologists or pharmacologists, respectively.

- Send an e-mail message to [actg.**cmc**A5208@fstrf.org](mailto:actg.cmcA5208@fstrf.org) (ATTN: Susan Eshleman and John Mellors [Virologists], or Francesca Aweeka and Chiedza Maponga [Pharmacologists]).

Data Management

For non-clinical questions about inclusion/exclusion criteria, the case report forms (CRFs), the CRF schedule of events, randomization/registration, transfers, delinquencies, and other data management issues, contact the data manager.

- **For transfers, reference the Patient Transfer from Site to Site SOP 119, and contact Ann Walawander and Apsara Nair directly (**[**walawander.ann@fstrf.org**](mailto:walawander.ann@fstrf.org) **and** [**nair@fstrf.org**](mailto:nair@fstrf.org)**).**
- **For other questions, s**end an e-mail message to [actg.**cmc**A5208@fstrf.org](mailto:actg.cmcA5208@fstrf.org) (ATTN: **Ann Walawander and Apsara Nair**).
- Include the study number (A5208), PID, and a detailed question.

Randomization Questions

For randomization questions or problems, or SID lists, contact the Statistical and Data Analysis Center (SDAC)/Data Management Center (DMC) programmers.

- Call the SDAC/DMC Randomization Desk at (716) 898-7301 or
- E-mail [aactg.support@fstrf.org](../ProtShell.doc).

Computer Questions

For computer or screen problems, contact the SDAC/DMC programmers.

- Call the SDAC/DMC programmers at (716) 834-0900, x7302 or
- E-mail [aactg.support@fstrf.org](../ProtShell.doc).

Protocol Questions

For protocol document questions, contact the Clinical Trials Specialist.

- Send an e-mail message to [actg.**cmc**A5208@fstrf.org](mailto:actg.cmcA5208@fstrf.org) (ATTN: Evelyn Hogg).

Copies of Protocol

To request copies of the protocol:

- Hard copies: Send an e-mail message to [ADULT.OPS@fstrf.org](mailto:ADULT.OPS@fstrf.org) (ATTN: Diane Delgado).
- Electronic copies can be downloaded from the Members area of the ACTG Web site (http**s**://**www.actgnetwork.org**).

Protocol Registration

A preliminary review of the registration packet for completeness will be performed at the ACTG Operations Center. Sites must send their registration packet via e-mail to [ICTUprotocol@s-3.com](mailto:ICTUprotocol@s-3.com) or call (301) 628-3474, with any questions about this preliminary review. The original protocol registration packet will be submitted to the DAIDS Regulatory Compliance Center (RCC) for approval within 24 hours of receipt at the ACTG Operations Center, unless omissions are noted during the review.

Registration Approval Questions

For follow-up questions about protocol registration approval:

- Send an e-mail message to [**mailto:Protocol@tech-res.com**](mailto:Protocol@tech-res.com)
- Call (301) 897-1707

Study Drug Questions

For questions or problems regarding study drug, dose, supplies, records, or returns, contact Lynette Purdue, Protocol Pharmacist:

- Phone: (301) 435-3744 or
- E-mail: [**lpurdue@niaid.nih.gov**](mailto:lpurdue@niaid.nih.gov)

Ordering Study Drug

To order study provided drugs:

- Call the Clinical Research Products Management Center at (301) 294-0741.

Study Drug Information

To request a copy of a package insert or an investigator brochure for any study drugs contact the RCC Safety Information Center:

- [**RIC@tech-res.com**](mailto:RIC@tech-res.com).

Expedited Adverse Events (EAEs)

For questions about EAE reporting, contact the DAIDS through the RCC.

- Send an e-mail message to [RCCSafetyOffice@tech-res.com](../../../../C:/Documents%20and%20Settings/projects/Aactg/PROTDEV/Protocol-Shells-&-Consent-Shells/Protocol-Shell/SafetyOffice@tech-res.com) or
- Call 1 (800) 537-9979 or (301) 897-1709 or
- Fax: 1-(301) 897-1710

Phone Calls to Protocol Team Members

Any phone calls must be documented by e-mail to [actg.teamA5208@fstrf.org](mailto:actg.teamAXXXX@fstrg.org) . This will be the site’s responsibility.

Web Pages

Additional information concerning study management of ACTG studies can be found on the ACTG WEB page ([**https://www.actgnetwork.org**](https://www.actgnetwork.org/)). Information specific to this study can be found on the protocol-specific Web page (PSWP) **located on the ACTG website.**

###### GLOSSARY

ACTG AIDS Clinical Trials Group

AE adverse events

ART antiretroviral therapy or treatment

ARV antiretroviral drug

ATV atazanavir

AUC area under the curve

CK creatine kinase

**CMC clinical management committee**

CNS central nervous system

CRF case report form

**CSS Community Scientific Subcommittee**

d4T stavudine

DAIDS Division of AIDS

ddI didanosine

ddI EC didanosine with enteric coating

**DSMB Data and Safety Monitoring Board**

EAE Expedited adverse event (reporting)

EC ethics committee

EFV efavirenz

FDA Food and Drug Administration

FTC emtricitabine

**GI gastrointestinal**

HIVNET HIV Network

HPTN HIV Prevention Trials Network

IRB institutional review board

ITT intent to treat

3TC lamivudine

LPV lopinavir

MTCT mother to child transmission

NNRTI nonnucleoside reverse transcriptase inhibitor

NRTI nucleoside reverse transcriptase inhibitor

NVP nevirapine

OCTANE Optimal Combined Therapy After Nevirapine Exposure

PBMC peripheral blood mononuclear cells

PCR polymerase chain reaction

PI protease inhibitor

PTT partial prothrombin time

QOL quality of life

RCT randomized clinical trial

**RLS resource-limited setting**

RT reverse transcriptase

RTV ritonavir

RU resource utilization

SD single dose

SGS single genome sequencing

**SoC standard of care**

TB tuberculosis

TDF tenofovir disoproxil fumarate

ZDV zidovudine

###### SCHEMA

A5208/OCTANE

Optimal Combination Therapy After Nevirapine Exposure

DESIGN A5208/OCTANE is a phase III study comprising two randomized clinical trials (RCT) to be conducted concurrently. Both trials will compare the virologic response to non-nucleoside reverse transcriptase inhibitor (NNRTI)-based (Arm 1A) versus protease inhibitor (PI)-based (Arm 1B) antiretroviral treatment (ART) in HIV-infected treatment-naïve women.

Trial 1 will evaluate the superiority of PI-based ART over NNRTI-based ART in women with prior single dose (SD) nevirapine (NVP) prophylaxis for mother-to-child-transmission (MTCT) of HIV. Trial 2 will evaluate the equivalence of PI- and NNRTI-based ART in women with no prior NVP exposure.

There will be **3** steps in this study. At entry, all participants will be registered to Step 1.

DURATION **The total duration of the study will be up to 132 weeks following randomization of the final participant.**

SAMPLE SIZE **740** evaluable participants total; participants who do not initiate treatment within 1 week after randomization will be replaced.

Trial 1: 240 participants with prior receipt of SD NVP MTCT prophylaxis randomized (1:1) to either Arm 1A or Arm 1B.

Trial 2: **500** participants with no prior NVP exposure randomized (1:1) to either Arm 1A or Arm1B.

POPULATION HIV-infected, treatment-naïve women, at least 13 years of age, or minimum age of local IRB consent, with CD4+ count <200 cells/mm^3^.

STRATIFICATION Within each trial, participants will be stratified using their screening CD4+ cell count as follows: <50 or ≥50 cells/mm^3^.

REGIMEN STEP 1: Initial Regimen

At study entry, participants will enter Step 1 and be randomized (as described above) to one of the following treatment arms:

Arm 1A

nevirapine (NVP) 200 mg po QD in the AM for 14 days, then NVP 200 mg po BID PLUS

emtricitabine (FTC) 200 mg po QD PLUS

tenofovir (TDF) 300 mg po QD

OR

Arm 1B

lopinavir/ritonavir (LPV/RTV) 400/100 mg po BID PLUS

FTC 200 mg po QD PLUS

TDF 300 mg po QD

NOTE : FTC and TDF may be replaced in either arm with the fixed dose combination drug Truvada, as follows:

FTC/TDF 200 mg/300 mg co-formulated tablet po QD

Study drugs will be provided for all participants through 48 weeks after the final participant is randomized.

STEP 2: Second Regimen

Participants on Step 1 who discontinue NVP or LPV/RTV (with or without simultaneously discontinuing one or more NRTIs), either because of virologic failure, toxicity, or intolerability, may enter Step 2 only if they switch to LPV/RTV or NVP, respectively. Substitution of one or two NRTIs for reasons of toxicity or intolerance (without change in NVP or LPV/RTV) will not lead to a step change.

NOTE: For participants whose baseline plasma HIV-1 RNA level is ≤750,000 copies/mL, virologic failure is defined as a plasma HIV-1 RNA level that is <1 log_10_ below baseline 12 weeks after treatment is initiated OR as a plasma HIV-1 RNA level that is ≥400 copies/mL at or after 24 weeks of treatment.

For participants whose baseline HIV-1 RNA level is >750,000 copies/mL, virologic failure is defined as a plasma level that is >75,000 copies/mL 12 weeks after treatment is initiated, OR as a plasma HIV-1 RNA level that is ≥400 copies/mL at or after 24 weeks of treatment.

A plasma HIV-1 RNA level that is <400 copies/mL 12 weeks after treatment is initiated is NOT considered an indication of virologic failure, regardless of the participant’s baseline plasma HIV-1 RNA level.

In all cases, the initial indication of virologic failure should be confirmed (ideally within 2 to 4 weeks and no later than the next attended visit). The baseline value will be the value obtained at the study entry visit. If this value is not available, then the baseline value will be the screening value.

Participants randomized to Arm 1A treatment will switch to:

Arm 2A

LPV/RTV 400/100 mg po BID PLUS

2 or more NRTIs

Participants randomized to Arm 1B treatment will switch to:

Arm 2B

NVP 200 mg po QD for 14 days, then NVP 200 mg po BID PLUS

2 or more NRTIs

Choice of NRTIs is at the discretion of the site investigator and may include any potentially viable combination of the study-provided drugs used in Step 1, and/or additional NRTIs provided by A5208/OCTANE, and/or drugs not available through A5208/OCTANE.

**STEP 3: Transition to Local Care**

**Participants will be registered to Step 3 at the time of their final Step 1 or 2 visit. Participants who enter Step 3 will be followed for an additional 72 weeks while receiving their HIV care and treatment through local treatment programs. Where possible, participants should continue to receive the same ART they had been taking in A5208/OCTANE, including the NRTI backbone. Participants will be seen at the A5208/OCTANE study clinic 12 weeks and 72 weeks after registration to Step 3.**

# 1.0 HYPOTHESIS AND STUDY OBJECTIVES

## 1.1 Hypothesis

Prior SD NVP prophylaxis compromises the subsequent response to NNRTI-based ART. This will be addressed by evaluating the following three sub-hypotheses:

i. Among women with prior NVP prophylaxis but no prior ART, a PI-based regimen is more effective in suppressing HIV-1 RNA than an NNRTI-based regimen.

ii. Among women with no prior NVP exposure and no prior ART, the effect of NNRTI- and PI-based regimens in suppressing HIV-1 RNA will be similar.

iii. The difference in the effect of PI- versus NNRTI-based regimens is associated with whether or not a woman previously received NVP prophylaxis.

## 1.2 Primary Objectives

1.2.1 In Trial 1 (participants with prior NVP prophylaxis), to compare the time to virologic failure or death between participants initiating ART with a regimen including NVP versus a regimen including LPV/RTV.

1.2.2 In Trial 2 (participants with no prior NVP exposure), to compare the time to virologic failure or death between participants initiating ART with a regimen including NVP versus a regimen including LPV/RTV.

1.2.3 To evaluate the difference in the effect of NNRTI-based and PI-based ART on the time to virologic failure or death in participants with prior NVP prophylaxis (Trial 1) versus no prior NVP exposure (Trial 2).

NOTE: For participants whose baseline plasma HIV-1 RNA level is ≤750,000 copies/mL, virologic failure is defined as a plasma HIV-1 RNA level that is <1 log_10_ below baseline 12 weeks after treatment is initiated OR as a plasma HIV-1 RNA level that is ≥400 copies/mL at or after 24 weeks of treatment.

For participants whose baseline HIV-1 RNA level is >750,000 copies/mL, virologic failure is defined as a plasma level that is >75,000 copies/mL 12 weeks after treatment is initiated, OR as a plasma HIV-1 RNA level that is ≥400 copies/mL at or after 24 weeks of treatment.

A plasma HIV-1 RNA level that is <400 copies/mL 12 weeks after treatment is initiated is NOT considered an indication of virologic failure, regardless of the participant’s baseline plasma HIV-1 RNA level.

In all cases, the initial indication of virologic failure should be confirmed (ideally within 2 to **4 weeks and no later than the next** attended **visit**). The baseline value will be the value obtained at the study entry visit. If this value is not available, then the baseline value will be the screening value.

## 1.3 Secondary Objectives

1.3.1 To compare the proportion of participants in each treatment arm with drug-resistant virus at entry and at virologic failure, as determined by bulk sequencing.

1.3.2 To determine the tolerability and safety of the study drug regimens.

- - 1. To evaluate the effect of prior NVP exposure on virologic failure through the assessment of genetic relatedness of HIV minority variants present at baseline with HIV variants selected at virologic failure in participants receiving NNRTI and/or PI-based regimens.
    2. To evaluate virologic response to a second regimen after NVP or LPV/RTV is changed due to virologic failure as defined above.
    3. To evaluate factors (e.g. HIV-1 subtype, disease status) in diverse geographical locations that are associated with risk of virologic failure and antiretroviral drug resistance.
    4. To compare changes in CD4+ cell counts and HIV-related disease progression and mortality in each treatment arm.
    5. To determine if NVP drug exposure (parent drug) as estimated by pharmacokinetic measurements correlates with development of NVP-associated rash and/or hepatitis.
    6. To evaluate adherence to study drug regimens by participant self-report and pill count.
    7. To compare resource utilization and quality of life in participants randomized to Arm 1A versus Arm 1B.
    8. **To evaluate short-term toxicity and virologic, immunologic, and clinical outcomes after participants are transitioned to local non-A5208-provided HIV care and treatment programs at the completion of either Step 1 or 2.**
    9. **To evaluate longer-term toxicity and virologic, immunologic, and clinical outcomes after participants are transitioned to local non-A5208-provided care and treatment programs at the completion of either Step 1 or 2.**

# 2.0 INTRODUCTION

## 2.1 Background

Since the initial demonstration that significant reduction in the rates of mother to child transmission (MTCT) of HIV-1 can be achieved with antiretroviral drug (ARV) interventions in the perinatal period [Sperling, 1996], there have been dramatic reductions in the rates of neonatally acquired HIV-1 infection in locations where perinatal transmission prevention programs have been instituted. A variety of ARV regimens and therapeutic strategies have been studied in different geographical settings, and virtually all studies have demonstrated reductions of similar or greater magnitudes as ACTG 076 [Shaffer N, 1999; PETRA Study Team, 2002]. One of the more dramatic results merged from the HIVNET 012 study demonstrating that a single dose of nevirapine (NVP) delivered to the mother at the time of delivery was capable of reducing transmission by as much as 47% [Guay, 1999; Jackson, 2003]. This strategy takes advantage of the potency, rapid absorption, high transplacental passage, and the prolonged pharmacokinetics of NVP and the relative ease of administering a single dose of the drug in the immediate pre-delivery period.

A potential downside of using SD NVP for prevention of HIV-1 MTCT is the selection of nonnucleoside reverse transcriptase inhibitor (NNRTI)-resistant virus in the plasma of many women after NVP administration [Eshleman, Becker-Pergola, 2001; Beckerman, 2003, Kantor et al. 2003]. In HIVNET 012, NNRTI-resistant virus variants were detected in 25% of women 6-8 weeks after SD NVP using the ViroSeq genotyping assay [Eshleman, Guay, 2003]. In HIVNET 023, NNRTI-resistant virus variants were detected in 75% of HIV-1-infected women 2 weeks after receiving SD NVP using the TRUGENE genotyping assay but in only 34% of women 8 weeks after SD NVP, again using TRUGENE genotyping assay [Kantor et al., 2003]. NVP resistance was also analyzed in the SAINT trial, in which women received two doses of NVP for prevention of MTCT. NVP resistance was detected with the TRUGENE assay in 67% of women 4-6 weeks after two doses of NVP [Sullivan, 2002]. This initially suggested that the administration of two doses of NVP, rather than a single dose, might enhance selection of NVP resistant variants. Further analysis, however, suggests that the higher rate of resistance reported in the SAINT trial may instead reflect the earlier time of collection of samples for resistance testing (4-6 weeks postpartum in SAINT vs. 6-8 weeks in HIVNET 012, and 8 weeks postpartum in HIVNET 023).

In HIVNET 012, NNRTI resistance mutations faded from detection by 12-24 months in all 11 women who had follow-up samples available [Eshleman, Mracna, 2001], and in HIVNET 023, those mutations faded from detection by 24 weeks in 7 out of 8 women who had follow-up samples available [Kantor et al., 2003]. The finding of detectable NVP resistance in 8 out of 36 (22%) women 12 months after two doses of NVP in the SAINT trial [Sullivan, 2002] suggests that the administration of a second dose of NVP may affect the rate at which NVP-resistant virus variants fade from detection in this setting. If this were true, it might affect the rate of NVP resistance and/or its persistence in selected women who received two doses of NVP prior to a delivery due to false labor.

The population sequencing-based HIV-1 genotyping assays used in the studies cited above are designed to detect only the major HIV-1 population. Other research assays (see below) are designed to detect drug-resistant virus variants present at lower levels. In HIVNET 012, analysis of individual HIV-1 variants by cloning revealed diverse NNRTI resistance mutations in women 7 days after SD NVP, many of which were not detected by routine population sequencing assays [Eshleman, Jones, 2003]. Therefore, studies using population sequencing approaches may tend to underestimate the number of women who have selection of NNRTI-resistant virus variants after SD NVP. Furthermore, while resistant virus detected in plasma gradually fades from detection using population sequencing assays, minority variants may still persist in plasma. The replication capacity (fitness) of HIV-1 with the K103N mutation is similar to that of “wild type” HIV-1 [Collins, 2003; Imamichi, 2001; Gerondelis, 1999], favoring persistence of those variants over time [Pao, 2004]. NNRTI-resistant proviral HIV-1 DNA may also be archived in cellular reservoirs.

A number of techniques have been developed that enable quantitative analysis of the prevalence of low frequency viral variants in a viral quasispecies. These include single genome sequencing (SGS) methods [Kearney, 2003], resistance testing using the Ty-1 HIV reverse transcriptase (RT) retrotransposon system [Nissley, 2003], and allele-specific RT- polymerase chain reaction (PCR). The SGS methods and the Ty-1 HIV RT system have recently been applied to the study of minor variants in patients participating in ACTG 398 [Mellors, 2004]. In this study, patients who might have been previously treated with NNRTIs were randomized to several salvage therapy regimens and treatment responses were monitored [Hammer, 2002]. This study demonstrated that standard bulk sequencing techniques were not able to detect low frequency NNRTI-resistance mutations. In contrast, both the SGS methods and the Ty-1 HIV RT system could detect low frequencies of NNRTI-resistant viruses in a substantial fraction of patients previously treated with NNRTI-based regimens [Mellors, 2004]. Furthermore, analysis of genetic relatedness of viruses isolated at the time of virologic failure in patients receiving efavirenz (EFV)-based regimens demonstrated that many (but not all) of the viruses emerging at treatment failure were genetically very closely related to minor viral variants detected prior to initiation of therapy [Mellors, 2004]. This result suggests that the minor quasispecies of NNRTI-resistant viruses played an important role in treatment response. In another study, a sensitive assay based on real-time PCR was able to detect variants with the K103N NNRTI resistance mutations in patients failing NVP-containing regimens. Those mutations were not detected by population sequencing assays. The presence of low levels of K103N variants following NVP treatment may explain why patients previously treated with NVP often fail subsequent treatment with EFV [Lecossier, 2003].

HIV-1 subtype may be an important parameter in A5208/OCTANE. A variety of group M subtypes are expected to be prevalent at different international sites, which could affect the rate of NVP resistance following SD NVP. In HIVNET 012, the rate of NVP resistance detected at 6-8 weeks postpartum was higher for women with subtype D than subtype A virus (35.7% vs. 19%, respectively, p=0.0035) [Eshleman, Becker-Pergola, 2001; Eshleman, Guay, 2003]. This difference did not appear to reflect more advanced disease among women with subtype D vs. subtype A [Eshleman, Guay, 2003]. Combined data from Ugandan women in HIVNET 012 and Zimbabwean women in HIVNET 023 suggest that the rate of NVP resistance for women with subtype C may be similar to that for women with subtype D [Kantor, Zijenah, 2002]. However, it is difficult to compare the rates of resistance in these two trials, since different assays were used for resistance testing. Even if the transient selection of NVP resistance after SD NVP does not influence subsequent treatment with NNRTIs, the natural susceptibility of HIV-1 to ARVs may be influenced by subtype [Descamps, Apetrei, 1998; Apetrei, Descamps, 1998]. Polymorphisms associated with drug resistance are frequently detected in ARV-naïve individuals with non-subtype B infection [Pieniazek, 2000]. Such subtype-based differences may influence the rate at which a specific mutation emerges, and the type of amino acid selected at a given position under drug pressure [Turner, 2004]. Differences in sequences of non-subtype B viruses may also lead to emergence of novel subtype-specific drug resistance mutations at positions not associated with drug resistance in subtype B [Gomes, 2003]. Few studies have actually evaluated the virologic response to antiretroviral treatment (ART) of HIV-1-infected individuals with different subtypes, and those that have are generally rather small and have included patients treated with varying regimens [Frater, 2001]. Some of these issues will be addressed in trials such as A5175 and HPTN 052. A5208/OCTANE provides an additional opportunity to learn more about the use of ART and the emergence of resistance in areas with different circulating subtypes, and would be among the first to examine these issues exclusively in women of childbearing age.

The implications of perinatal selection of NNRTI-resistant viruses by SD NVP prophylaxis for subsequent treatment responses to ART in the mother and for NVP prophylaxis in subsequent pregnancies are unclear. It is clear that prior selection of NNRTI-resistant virus in the setting of therapeutic use compromises the subsequent response to NNRTI-based regimens [Hammer, 2002; Antinori, 2002]. This is demonstrable regardless of the presence of circulating NNRTI-resistant virus when bulk sequencing techniques are used. [Mellors, 2004; Hammer, 2002]. It is not yet known whether persistent minority variants or archived NNRTI-resistant virus selected by SD NVP will also compromise the subsequent response to therapeutic use of NNRTI-based regimens. Since NVP is a molecule that induces its own hepatic metabolism, the administration of a single dose to a patient with a non-induced liver result in substantial plasma NVP levels for 2-4 weeks. In pregnant women during labor, the median half-life of NVP is 61.3 hours [Mirochnick, 1998; Musoke, 1999]. Thus, viral selection following SD NVP regimens for MTCT prophylaxis may be quite similar to that following several days or weeks of NVP monotherapy. During the early testing of NVP, drug resistant mutations could be easily detected 7-10 days after initiation of therapy [Richman, 1994]. It was also demonstrated that ongoing NVP selection resulted in the rapid dominance of resistant viruses [Havlir, 1996].

The only information available on the impact of SD NVP upon subsequent response to NVP-containing ART is early data from a MTCT prevention trial in Thailand. In this trial, all pregnant participants received short-course zidovudine (ZDV) from 28 weeks gestation to delivery, and mother-infant pairs were randomized into 3 groups: SD NVP to the mother and infant, NVP-NVP; SD NVP to the mother and placebo to the infant, NVP-placebo; and placebo to the mother and the infant, placebo-placebo. Only a small number of women received placebo before the placebo-placebo arm was terminated, due to higher MTCT rates in this arm than in the NVP-NVP arm [Lallemant, 2004]. Women with CD4+ cell count <250 cells/mm^3^ were subsequently offered NVP-based ART. After six months on ART, 76% of the 142 SD NVP-exposed women and 85% of the 27 non-exposed women had a viral load <400 copies/mL (p=0.30) [Jourdain, CROI 2004] and 92 of 188 (49%) and 28 of 41 (68%), respectively, had a viral load <50 copies/mL (p=0.03) [Jourdain, NEJM 2004]. Among the NVP-exposed women who started ART more than 6 months after delivery, 87% had a viral load <400 copies/mL 6 months after starting ART, compared with 85% of NVP non-exposed women, the vast majority of whom also started ART more than 6 months postpartum [Jourdain, CROI 2004]. CD4 cell increase and clinical outcomes did not differ between the NVP and placebo recipients [Jourdain et al, NEJM 2004]. While these data suggest that response to NVP-containing ART as measured by suppression of HIV-1 RNA <50 copies/mL (but not to <400 copies/mL) may be compromised by prior SD NVP exposure, the small numbers in the NVP non-exposed group temper conclusions in this regard. Furthermore, it is possible that the placebo recipients represented a group of patients with slower disease progression (and therefore potentially better innate response to ART) than the NVP recipients, given that the placebo recipients started ART so much later than the NVP recipients relative to delivery (but with similar pre-treatment CD4 and viral load). It remains uncertain whether women are more likely to fail therapy if they start ART shortly after exposure to SD NVP versus many months after this exposure. This Thai study of response to NVP-containing ART among SD NVP-exposed and non-exposed women also does not test the efficacy of an alternative regimen among SD NVP-exposed women.

There is an accelerating opportunity to prevent MTCT of HIV-1 with resources provided by a wide variety of agencies and foundations. The cost, simplicity, safety, and efficacy of SD NVP for prevention of HIV-1 MTCT make it an attractive choice in resource-limited settings, and this regimen is being implemented around the world. At the same time, access to combination ART in these settings is increasing significantly, and it is very likely that initial treatment regimens for adults and children in resource-limited settings will be NNRTI-based (and NNRTI-based first-line treatment regimens have already been chosen by national treatment programs in countries such as Botswana, South Africa, and Thailand). Therefore, there is a critical need for information about the implications of widespread MTCT NVP prophylaxis on subsequent responsiveness to therapy with NNRTI-based regimens. This information is being sought by several investigative groups and public health agencies. Most of these efforts are based on observational studies that will provide useful information but will not necessarily provide a rigorous context in which the data can be evaluated. Since virologic response rates to combination treatment range from 60% to 85% in treatment-naïve patient populations, depending on the regimen chosen and the patient population studied, observational data will be limited in terms of interpretability and will not shed light on potentially more efficacious alternative approaches. A rigorous answer will require randomized clinical trials that compare treatment responses to NNRTI-based versus non-NNRTI-based regimens in populations with and without prior NVP exposure. This study (A5208/OCTANE) seeks to compare the treatment responses to NNRTI-based regimens in women who have or have not received SD NVP prophylaxis and to compare these responses to treatment responses to PI-based regimens in the same patient populations. It is anticipated that this study will determine whether treatment responses to NNRTI-based regimens are compromised in women who have received prior SD NVP prophylaxis and will also provide insight into whether a PI-based regimen would fare better in this setting.

**In October 2008, following an unplanned review of efficacy data (after a regularly scheduled meeting in September 2008 at which accrual and safety data were reviewed), the Data and Safety Monitoring Board (DSMB) stated that “Data indicate that the LPV/RTV treatment arm is significantly superior to the NVP treatment arm in Trial 1 (participants with prior NVP prophylaxis).” The DSMB further recommended that results from Trial 1 be unblinded [released] and treatment and safety data be made public as soon as possible but that Trial 2 results remain blinded.**

**Key results from Trial 1 based on data collected through 6 October 2008 [Lockman, CROI 2009]**

- **243 women were randomized in Trial 1 (123 to NVP arm, 120 to LPV/RTV arm) in 10 sites. 241 women started study treatment in Step 1 (121 in NVP arm, 120 in LPV/RTV arm); results below are based on data from these 141 participants.**
- **Only 2.5% of participants were lost to follow-up overall (only 2 participants, or 0.8%, were lost to follow-up before reaching a primary endpoint).**
- **The median duration of follow-up in Trial 1 was 73 weeks.**
- **Characteristics of Trial 1 participants at study entry were well balanced between arms: median age 31 years; median CD4+ count 139 cells/mm^3^; and median HIV-1 RNA 5.15 log_10_ copies/mL.**
- **At randomization, the median time since last use of SD NVP was 17 months.**
- **All Trial 1 participants self-reported ingesting SD NVP before study entry; written documentation to support that NVP had been provided was available for 73% of participants.**
- **44 women discontinued Step 1 treatment (NVP or LPV/RTV): 38 (31%) in NVP arm and 6 (5%) in LPV/RTV arm (p<0.0001).**
- **41 women reached a primary endpoint (virologic failure or death). A significantly larger proportion of women in the NVP arm (31, or 26%) than women in the LPV/RTV arm (10, or 8%) reached a primary endpoint (virologic failure or death) (p=0.0007). Of these endpoints, 36 were virologic failure (27 in the NVP arm, 9 in the LPV/RTV arm), and 5 were death without preceding virologic failure (4 in the NVP arm, 1 in the LPV/RTV arm).**
- **None of the 5 deaths were reported to be treatment-related. For the one woman who died of extrapulmonary tuberculosis, no information was available for relationship to study medication.**
- **The difference in the risk of virologic failure or death (between the LPV/RTV and NVP treatment arms) was greatest in the first 12-24 weeks on therapy, but may be diminished after that period. For example, by week 12, there were 17 virologic failures or deaths among women in the NVP arm compared with 4 among women in the LPV/RTV arm (varying by a factor of 4.25). After week 12, there were 14 vs. 6 events (varying by a factor of 2.33). However, the numbers of events after week 12 are very small, so it is difficult to evaluate whether or not the difference favoring LPV/RTV decreases over time after week 12.**
- **Baseline genotype results were available for 239 of the 241 Trial 1 participants. Thirty-three (14%) of the 239 women in Trial 1 with available results had NVP drug resistance mutations at baseline. The difference in outcomes between NVP and LPV/RTV was greater among women with baseline NVP resistance than among women without baseline NVP resistance.  Among the women with NVP resistance at baseline, 11 of the 15 (73%) in the NVP arm versus 1 of the 18 (6%) in the LPV/RTV arm experienced a primary endpoint. In contrast, among women without NVP resistance at baseline, the corresponding proportions were 19 of 105 (18%) and 9 of 101 (9%), respectively. There was some evidence that the difference between treatment arms varied according to the presence or not of NVP resistance at baseline (interaction p=0.040).**
- **There was an apparent trend toward decreasing difference (in primary endpoint) between NVP and LPV/RTV treatment arms with increasing time between last prior SD NVP exposure and treatment initiation (although this trend did not reach statistical significance when tested in the pre-specified manner):**
  - **Among women starting antiretroviral therapy 6 to <12 months after SD NVP, 15 [37%] vs. 1 [3%] of NVP and LPV/RTV arms reached an endpoint (p=0.008).**
  - **With duration since last exposure 12 to < 24 months, 11 [26%] vs. 4 [12%] reached an endpoint (p=0.056)**
  - **In women who took their last SD NVP at least 2 years earlier, 3 [12%] vs. 3 [10%] met an endpoint (p=0.72).**
  - **The p value for this interaction (of the difference between treatment arms and continuous time since last SD NVP) = 0.20**
- **The difference between the NVP and LPV/RTV treatment arms did not appear to be associated with the availability (or lack thereof) of written documentation of SD NVP exposure.**

**The decision as to whether to switch from NVP to LPV/RTV vs. to continue NVP is at the discretion of the study participant and investigator.**

## 2.2 Antiretrovirals

In developed countries, standard treatment for persons who are naïve to ARVs consists of three-drug combinations of two nucleoside reverse transcriptase inhibitors (NRTIs) with either an HIV-1 protease inhibitor (PI) or an NNRTI. These regimens provide effective inhibition of HIV-1 replication that results in functional immune reconstitution, a decrease in the risk of AIDS-related events, and improved survival [D’Aquila, 1996; Hammer, 1997; Gulick, 1997; Autran, 1997; Palella, 1998]. The World Health Organization ART guidelines for resource-limited settings recommend regimens comprising two NRTIs with one NNRTI as first-line regimens, taking into account efficacy, toxicity, cost and availability, and cold chain requirements of various regimens [WHO, 2003]. PI-based regimens may, however, have superior efficacy in women who have previously taken SD NVP (one of the hypotheses being tested in A5208/OCTANE).

The cost and availability of ARVs in resource-limited settings change rapidly; in 2000, the yearly cost of a three-drug antiretroviral regimen for one patient was approximately $15,000 per year in such settings, and is as low as $150 per year in 2004. It is important to establish the efficacy and tolerability of available antiretrovirals in sub-Saharan Africa, where ART is increasingly available and where 70% of persons living with HIV reside.

2.2.1 Nevirapine (NVP, Viramune®)

NVP is an NNRTI with activity against HIV-1 and is structurally a member of the dipyridodiazepinone chemical class of compounds. NVP binds directly to RT and blocks the RNA-dependent and DNA-dependent DNA polymerase activities by causing disruption of the enzymes’ catalytic site. The activity of NVP does not compete with template or nucleoside triphosphates. HIV-2 RT and eukaryotic DNA polymerases (such as human DNA polymerases alpha, beta, gamma, or delta) are not inhibited by NVP.

Clinical Experience

In the ACTG 241 study, heavily pretreated participants with CD4+ cell counts ≤350 cells/mm^3^ and ≥6 months of prior NRTI therapy were randomly assigned to therapy with open-label ZDV (600 mg/day) plus ddI (400 mg/day) and double-blinded placebo or NVP (200 mg/day for 2 weeks, then 400 mg/day). At 48 weeks, participants on the NVP arm had an 18% higher mean absolute CD4+ cell count (p=0.001) and a 0.25 log_10_ lower plasma HIV-1 RNA (p=0.028) compared with those on the placebo arm. Risk of disease progression was not statistically different between the two arms (p>0.2). The major toxicity was severe rash (9%) in the triple-therapy arm, which was attributed to NVP.

Recent results from the Atlantic Trial were reported when NVP (400 mg/day) was combined with didanosine (ddI) and stavudine (d4T) in ARV-naïve HIV-infected participants. In this study, d4T and ddI were combined with either NVP, indinavir (IDV) or 3TC. After 48 weeks of follow-up, the percentage of participants with HIV RNA ≤50 copies/mL using an intent-to-treat (ITT) analysis was 49% in the NVP arm, 49% in the IDV arm, and 40% in the triple nucleoside arm.

Week 24 results for the VIRGO trial (n=40 participants) for the NVP 400 mg QD plus d4T/ddI regimen showed a mean −2.49 log_10_ decrease in plasma HIV RNA and a mean CD4+ cell count increase of 152 cells/mm^3^. Viral suppression to <500 copies/mL and <50 copies/mL occurred in 68% and 48% of participants, respectively, based on an ITT analysis.

The 2NN study randomized 1216 treatment-naive patients to NVP QD, NVP BID, EFV, or NVP plus EFV (with an NRTI backbone of d4T and 3TC). Rates of treatment failure at 48 weeks were 43.7% in the NVP BID arm and 38.3% in the EFV arm [van Leth, 2003]. Percentages of patients with virologic suppression (<50 copies/mL) at 48 weeks were 65.4% in the NVP BID arm and 70.0% in the EFV arm. These differences were not statistically significant.

Genotypic changes associated with the in vivo use of NVP include the RT mutations K103N, V106A, Y181C/I, Y188C, and G190A. The K103N mutation is not uniformly predominant in isolates derived from HIV-infected participants treated over 48 weeks with ZDV/ddI/NVP, as <20% of participants failing this regimen had K103N.

Safety Profile

The safety of NVP has been assessed in more than 2800 patients in clinical trials. The experience from clinical trials and clinical practice has shown that the most serious AEs are clinical hepatitis/hepatic failure, Stevens-Johnson syndrome, toxic epidermal necrolysis, and hypersensitivity reactions characterized by rash, constitutional findings, and organ dysfunction. Fatalities due to these serious AEs have been reported. NVP is not to be taken BID until at least 14 days of QD dosing.

Hepatic Toxicity

In clinical trials, the overall NVP-attributable risk of hepatitis is approximately 1%. Increased AST (SGOT) or ALT (SGPT) values before the start of ART and/or history of hepatitis B or C infection are associated with a greater risk of hepatic AEs. Cases of hepatitis, severe and life-threatening hepatotoxicity, and fatal fulminant hepatitis have been reported in patients treated with NVP. Acute hepatotoxicity has progressed to hepatic failure with transaminase elevation, with or without hyperbilirubinemia, prolonged partial thromboplastin time (PTT), or eosinophilia. Hepatic dysfunction may be isolated or associated with signs of hypersensitivity. Symptoms of clinical hepatitis include fatigue, malaise, anorexia, nausea, jaundice, acholic stools, liver tenderness, or hepatomegaly. NVP-related hepatotoxicity can occur in the setting of normal serum transaminase or in the presence of a possible alternative diagnosis.

During the first 12 weeks of therapy, intensive monitoring of patients is required to detect potentially life-threatening hepatic events and skin reactions. However, approximately one third of cases have been reported to occur after the critical 12-week period. The optimal frequency of monitoring has not been established. Some experts recommend clinical and laboratory monitoring more often than once per month, and in particular, would include monitoring of liver function tests at baseline, prior to dose escalation and at 2 weeks post-dose escalation. After the initial 12-week period, clinical and laboratory monitoring every 2-3 months should continue throughout NVP treatment.

In study FTC 302, 468 patients were randomized to emtricitabine (FTC) or 3TC plus a background of d4T and either NVP (if screening HIV-1 RNA was <100,000 copies/mL, n=385) or EFV (if screening HIV-1 RNA was ≥100,000 copies/mL, n=83). Seventy-four percent of the patients completed 48 weeks of therapy. Overall, hepatotoxicity [defined as treatment-emergent Grade 3 (5 to 10 times the upper limit of normal) (ULN) or Grade 4 (greater than 10 times ULN) elevation in hepatic transaminase levels] occurred in 66 of the 468 patients. Hepatotoxicity was observed in 66 (17%) of patients receiving NVP compared with none receiving EFV. In 53 of these cases, the elevations were reported within the first 12 weeks of therapy. Rates of hepatic dysfunction were comparable between the randomized treatment arms of FTC versus 3TC. The hepatotoxicity was significantly and temporally associated with rash, nausea, fever and increased eosinophil count. Two patients developed liver failure and died. The risk of toxicity was greater in females with a BMI < 18.5 and albumin <35 g/dL [Sanne, 2005].

Physicians and participants should be vigilant for the appearance of signs or symptoms of clinical hepatitis or hypersensitivity reaction. Participants should be instructed to seek medical attention if they develop any of the symptoms of clinical hepatitis or hypersensitivity reaction. Liver function tests should be performed if a patient experiences signs or symptoms suggestive of clinical hepatitis and/or hypersensitivity reaction. The diagnosis of hepatotoxicity should be considered, even if liver function tests are initially normal or alternative diagnoses are possible, and monitoring should be done more frequently.

Rash and Hypersensitivity

The most common clinical toxicity of NVP is rash. Severe and life-threatening skin reactions, including fatal cases, have occurred in patients treated with NVP. These have included cases of Stevens-Johnson syndrome, toxic epidermal necrolysis, and hypersensitivity reactions characterized by rash, constitutional findings, and organ dysfunction. NVP-attributable rash occurred in 16% of patients on combination regimens in phase II/III controlled studies. Thirty-five percent of patients treated with the NVP-containing regimen experienced rash compared with 19% of control group patients treated with ZDV plus ddI or ZDV alone. Severe or life-threatening rash occurred in 6.6% of NVP-treated patients compared with 1.3% of patients treated in the control groups.

Rashes are usually mild-to-moderate, maculopapular erythematous cutaneous eruptions, with or without pruritus, located on the trunk, face, and extremities. In adult phase II/III clinical trials, the majority of rashes occurred within the first 6 weeks of therapy. Severe rashes occurred most frequently within the first 28 days of treatment; 25% of the patients with severe rashes required hospitalization, and one patient required surgical intervention. Overall, 7% of patients discontinued NVP due to rash.

In one clinical trial, concomitant use of prednisone to prevent NVP-associated rash increased the incidence and severity of rash during the first 6 weeks of NVP
therapy. The use of prednisone to prevent NVP-associated rash is not recommended.

Participants should be advised to notify their health care providers promptly if they develop any rash or signs and symptoms of a hypersensitivity reaction. Signs and symptoms of hypersensitivity include, but are not limited to, severe rash or rash accompanied by fever, general malaise, fatigue, muscle or joint aches, blisters, oral lesions, conjunctivitis, facial edema, and/or hepatitis, eosinophilia, granulocytopenia, and renal dysfunction.

NVP-associated rash and severe hepatitis are more common in persons with higher CD4+ cell counts especially among women with CD4+ cell counts >250 cells/mm^3^ [Lyons F, 2003; Imperiale, 2002].

A recent letter to healthcare providers from the manufacturer of NVP outlined important information about the safety of NVP based on recent post-marketing surveillance data and further analysis of the Viramune clinical trial database. The letter noted that women with CD4+ cell counts >250 cells/mm^3^, including pregnant women receiving chronic treatment for HIV infection, are at 12-fold higher risk of hepatotoxicity. Some of these events have been fatal. This subset of patients was identified by analyses of CD4+ cell count at the time of initiation of Viramune therapy. The greatest risk of severe and potentially fatal hepatic events (often associated with rash) occurs in the first 6 weeks of Viramune treatment. However, the risk continues after this time and patients should be monitored closely for the first 18 weeks of treatment with Viramune. In some cases, hepatic injury progresses despite discontinuation of treatment.

Additional information can be found in the Viramune package insert.

2.2.2 Lopinavir/Ritonavir (ABT-378/Ritonavir; LPV/RTV; Kaletra®, Aluvia®)

Lopinavir (LPV, ABT-378) is a potent inhibitor of HIV-1 protease. When co-formulated with LPV, ritonavir (RTV) inhibits the CYP3A-mediated metabolism of LPV, thereby providing increased plasma levels of LPV. Lopinavir/ritonavir (LPV/RTV) in a single fixed-dose combination capsule (Kaletra) was evaluated and approved by the FDA in 2000 for use in combination with other ARVs for the treatment of HIV-1 infection. A tablet formulation of LPV/RTV received FDA approval in October 2005. Aluvia is the tablet formulation of Kaletra. This tablet has a red coating whereas the capsule has a yellow coating.

Clinical Experience

A phase III study (M98-863) evaluated the safety and efficacy of LPV/RTV plus d4T and 3TC versus nelfinavir (NFV) plus d4T and 3TC [Walmsley, 2002]. Randomized participants had no more than 14 days of any ART and no prior d4T or 3TC treatment. The primary efficacy analyses included the proportion of participants with HIV-1 RNA level <400 copies/mL at week 24 and the duration of virologic response through week 48. Overall, 326 participants were assigned to the LPV/RTV group and 327 to the NFV group. Baseline HIV-1 RNA level was 4.9 log_10_ for each group. Baseline CD4+ cell counts were approximately 260 cells/mm^3^ for each group. At 48 weeks, the proportion of participants with HIV-1 RNA levels <400 (<50) copies/mL by ITT (missing value = failure, M = F) analysis were 75% (67%) for the LPV/RTV group compared with 63% (52%) for the NFV group (p<0.001) [Proportion <400 (< 50) copies/mL on treatment was 93% (83%) versus 82% (68%)]. Mean changes in CD4+ cell counts were 207 cells/mm^3^ for the LPV/RTV group and 195 cells/mm^3^ for the NFV group. Overall, 2% of participants in the LPV/RTV group and 4% in the NFV group discontinued therapy at or before week 48 because of study drug-related AEs. No significant differences between the two treatment groups were noted in AEs, except for increases in triglycerides. Moderate and severe AEs for the LPV/RTV and NFV groups, respectively, were as follows: diarrhea (16% and 17%), nausea (7% and 5%), asthenia (4% and 3%), abdominal pain (4% and 3%), SGPT/ALT >5 x ULN (4% each), total cholesterol >300 mg/dL (9% and 5%), triglycerides >750 mg/dL (9% and 1%,p = <0.001), and amylase >2 x ULN (3% and 2%).

Durability of response has been demonstrated with LPV/RTV in ARV-naive patients in the above study with 79% of the 326 participants on the LPV/RTV arm maintaining virologic suppression (viral load of <400 copies/mL) at 96 weeks, compared with 58% on the NFV arm [King, 2002].

Safety Profile

LPV/RTV has been studied in 701 patients as combination therapy in phase I/II and phase III trials.  The most common AEs associated with LPV/RTV therapy were diarrhea and nausea, which were generally of mild-to-moderate severity. Rates of discontinuation of randomized therapy due to AEs were 5.8% in LPV/RTV-treated and 4.9% in NFV-treated patients in study M98-863. Pancreatitis has been reported in patients receiving LPV/RTV, although a causal relationship has not been established. The most common laboratory abnormalities in patients receiving LPV/RTV were elevations in triglycerides and cholesterol, which may be marked, and less commonly elevations in AST and ALT.

LPV/RTV has not been studied in African populations or in other areas in which non-B subtypes predominate. The safety and pharmacokinetics of LPV/RTV in pregnancy have not been established. There has been no evidence of teratogenicity with administration of LPV/RTV to pregnant rats or rabbits. In rats treated with maternally toxic dosage (100 mg LPV/50 mg RTV/kg/day), embryonic and fetal developmental toxicities (early resorption, decreased fetal viability, decreased fetal body weight, increased incidence of skeletal variations and skeletal ossification delays) were observed. In rabbits, no embryonic or fetal developmental toxicities were observed with maternally toxic dosage, where drug exposure was 0.6-fold for LPV and 1.0-fold for RTV of the exposures in humans at recommended therapeutic dose.

Additional information can be found in the most recent Kaletra and Aluvia package inserts.

2.2.3 Emtricitabine (FTC, Emtriva™)

Emtricitabine (FTC) (5-fluoro-1-(2R,5S)-[2-(hydroxymethyl)-1,3-oxathiolan-5-yl]cytosine) is a synthetic nucleoside analogue with activity against HIV-1 reverse transcriptase. FTC is the negative (-) enantiomer of a thio analogue of cytidine, which differs from cytidine analogues in that it has a fluorine in the 5-position. FTC is phosphorylated by cellular enzymes to form the active intracellular metabolite, emtricitabine 5’-triphosphate (FTC-TP), which is a competitive inhibitor of HIV-1 RT and terminates the growing DNA chain.

Pre-Clinical

The antiretroviral activity of FTC has been shown to up to 10-fold greater than Epivir® (lamivudine, 3TC) against HIV‑1 clinical isolates in vitro using human peripheral blood mononuclear cells (PBMCs), macrophages and laboratory adapted T-cell lines (Schinazi, 1992, Schinazi, 2003). Furthermore, FTC is incorporated approximately 9-fold more efficiently into viral DNA than 3TC while being 24-fold less efficiently incorporated in human mitochondrial DNA polymerase [Feng, 1998; Feng, 1999; Feng, 2001]. FTC selects the M184V mutation less often and less rapidly in vitro during serial passage experiments in human PBMCs [Schinazi, 1993].

FTC is rapidly absorbed following oral administration, with peak plasma concentrations occurring within 1-2 hours of dosing and with good oral bioavailability (93%) [Wang, 2001; Rousseau, 2001]. FTC has a long plasma half-life (10 hours) such that steady state concentrations following 200 mg once-daily dosing are above the concentration needed to produce 90% inhibition (IC_90_) of HIV replication for more than 80 hours [Wang, 2001; Rousseau, 2001; Wang, 2002]. The intracellular half-life of the active moiety, FTC-TP, is even longer, ~39 hours, supporting once-daily dosing [Wang et al., 2001, Rousseau et al., 2001, Wang, 2002]. In vivo, FTC produced a 1.9 log_10_ reduction in HIV-1 RNA when given as monotherapy to HIV-infected patients for two weeks [Rousseau, 2001]. When given as monotherapy to HIV-infected patients for 10 days, FTC 200 mg QD demonstrated significantly greater antiviral activity compared to 3TC 150 mg BID as determined by plasma HIV-1 RNA average area under the curve minus baseline (AAUCMB) (p<0.05) [Rousseau, 2003].

Clinical Experience

Two phase III controlled studies (FTC-301A, and FTC-303) provide the most information concerning the safety and efficacy of FTC in HIV-1-infected adults treated for extended periods with combinations of ART [Cahn et al., 2003].

Study FTC-301A was a 48 week, double-blind, active-controlled, multicenter study comparing FTC (200 mg) QD to d4T in combination with QD open-label ddI and EFV in 571 ARV-naïve patients with plasma HIV-1 RNA >5,000 copies/mL. Patients had a mean age of 36 years (range 18 to 69), 85% were male, 52% Caucasian, 16% African American and 26% Hispanic. Patients had a mean baseline CD4+ cell count of 318 cells/mm^3^ (range 5-1317) and median baseline plasma HIV-1 RNA of 4.9 log_10_ copies/mL (range 2.6-7.0). Thirty-eight percent of patients had baseline viral loads >100,000 copies/mL and 31% had CD4+ cell counts <200 cells/mL.

At week 48, FTC was statistically superior to d4T with 81% of the patients in the FTC treatment group achieving and maintaining plasma HIV-1 RNA >400 copies/mL compared with 68% of the patients in the d4T treatment group. Likewise, the proportion of patients who achieved and maintained plasma HIV-1 RNA <50 copies/mL was statistically significantly different with 78% of patients in the FTC treatment group compared with 59% of patients in the d4T treatment group. Additionally, FTC-treated patients had a statistically greater increase in CD4+ cell count at Week 48 with a mean increase from baseline of 168 cells/mm^3^ for the FTC group and 134 cells/mm^3^ for the d4T group. The proportion of patients with virologic failure was 3% in the FTC group and 11% in the d4T group. A statistically greater proportion of patients in the d4T group experienced an adverse event that led to study drug discontinuation through Week 48 than in the FTC group (13% versus 7%).

Study FTC-303 was a 48 week, open-label, active-controlled, multicenter study comparing FTC to 3TC in combination with d4T or ZDV and a PI or NNRTI in 440 patients who were on a 3TC-containing triple-ARV regimen for at least 12 weeks prior to study entry and had plasma HIV RNA ≤400 copies/mL [Wakeford, 2003].

Patients were randomized 1:2 to continue therapy with 3TC (150 mg BID) or to switch to FTC (200 mg QD). All patients were maintained on their stable background regimen. Patients had a mean age of 42 years (range 22-80), 86% were male, 64% Caucasian, 21% African American, and 13% Hispanic. Patients had a mean baseline CD4+ cell count of 527 cells/mm^3^ (range 37-1909) and median baseline plasma HIV-1 RNA of 1.7 log_10_ copies/mL (range 1.7-4.0). The median duration of prior ART was 27.6 months.

Through 48 weeks of therapy, there was no statistically significant difference between treatment groups in efficacy outcomes. The proportion of patients with virologic failure was 7% in the FTC arm and 8% in the 3TC arm. Through 48 weeks of therapy, the proportion of patients who achieved and maintained plasma HIV-1 RNA <400 copies/mL was 77% in the FTC arm and 82% in the 3TC arm. The difference was largely attributed to attrition from the study and not loss of virological activity. Likewise, the proportion of patients who achieved and maintained plasma HIV-1 RNA below 50 copies/mL was 67% in the FTC arm and 72% in the 3TC arm. The mean increase from baseline in CD4+ cell counts was 29 cells/mm^3^ in the FTC arm and 61 cells/mm^3^ in the 3TC arm. These findings support equivalent efficacy of FTC 200 mg once-daily and 3TC 150 mg administered twice daily [Wakeford, 2003].

Safety Profile

More than 2000 adult patients with HIV infection have been treated with FTC alone or in combination with other ARVs for periods of 10 days to 200 weeks in Phase I-III clinical trials. Assessment of adverse events is based on data from studies FTC-301A and FTC‑303 in which 571 treatment naïve (FTC-301A) and 440 treatment experienced (FTC-303) patients received FTC 200 mg (n=580) or comparator drug (n=431) for 48 weeks.

The most common adverse events that occurred in patients receiving FTC with other ARVs in clinical trials were headache, diarrhea, nausea, and rash event, which were generally mild to moderate severity. Approximately 1% of patients discontinued participation in the clinical studies due to these events. All adverse events were reported with similar frequency in FTC and control treatment groups with the exception of skin discoloration, which was reported with higher frequency in the FTC-treated group. Skin discoloration, manifested by hyperpigmentation on the palms and/or soles was generally mild and asymptomatic. A summary of FTC treatment emergent adverse events in Studies FTC-301A and FTC-303 is provided in Table 1 below.

Table 1. Selected Treatment Emergent Adverse Events (All Grades, Regardless
of Causality) Reported in ≥3% of FTC-Treated Patients in Either
Study FTC-301A or FTC-303 (0-48 weeks)

|  | FTC-301A | | FTC-303 | |
| --- | --- | --- | --- | --- |
| Adverse Event | FTC+  ddI+EFV  (n=286) | d4T+  ddI+ EFV  (n=285) | FTC + ZDV/d4T + PI/NNRTI  (n=294) | 3TC + ZDV/d4T + PI/NNRTI  (n=146) |
| Body as a Whole  Abdominal Pain  Asthenia  Headache | 14%  12%  22% | 17%  17%  25% | 8%  16%  13% | 11%  10%  6% |
| Digestive System  Diarrhea  Dyspepsia  Nausea  Vomiting | 23%  8%  13%  9% | 32%  12%  23%  12% | 23%  4%  18%  9% | 18%  5%  12%  7% |
| Musculoskeletal  Arthralgia  Myalgia | 5%  6% | 6%  3% | 3%  4% | 4%  4% |
| Nervous System  Abnormal dreams  Depressive disorders  Dizziness  Insomnia  Neuropathy/Peripheral  Neuritis  Paresthesia | 11%  9%  25%  16%  4%  6% | 19%  13%  26%  21%  13%  12% | 2%  6%  4%  7%  4%  5% | <1%  10%  5%  3%  3%  7% |
| Respiratory  Increased cough  Rhinitis | 14%  12% | 8%  10% | 14%  18% | 11%  12% |
| Skin  Rash Event^1^ | 30% | 33% | 17% | 14% |

^1^ Rash event includes rash, pruritus, maculopapular rash, urtcaria, vesiculobullous rash, pustular rash, and allergic reaction.

Laboratory abnormalities in these studies occurred with similar frequency in the FTC and comparator groups. Embryofetal toxicology studies of FTC in mice and rabbits have not shown increased incidence of malformations [Szczech, 2003]. A summary of Grade 3 and 4 laboratory abnormalities is provided in Table 2 below.

Table 2. Treatment Emergent Grade 3/ 4 Laboratory Abnormalities Reported in ≥ 1% of FTC-Treated Patients in Either Study FTC-301A or FTC-303

|  | FTC-301A | | FTC-303 | |
| --- | --- | --- | --- | --- |
| Number of Patients Treated | FTC+  ddI+EFV  (n=286) | d4T+  ddI+ EFV  (n=285) | FTC + ZDV/d4T + PI/NNRTI  (n=294) | 3TC + ZDV/d4T + PI/NNRTI  (n=146) |
| Percentage with Grade 3 or Grade 4 laboratory abnormality | 34% | 38% | 31% | 28% |
| ALT (>5.0 x ULN^1^) | 5% | 6% | 2% | 1% |
| AST (>5.0 x ULN) | 6% | 9% | 3% | <1% |
| Bilirubin (>2.5 x ULN) | <1% | <1% | 1% | 2% |
| Creatine kinase (>4.0 x ULN) | 12% | 11% | 11% | 14% |
| Neutrophils (<750 mm^3^) | 5% | 7% | 5% | 3% |
| Pancreatic amylase (>2.0 x ULN) | <1% | 1% | 2% | 2% |
| Serum amylase (>2.0 x ULN) | 5% | 10% | 2% | 2% |
| Serum glucose (<40 or >250 mg/dL) | 2% | 3% | 3% | 3% |
| Serum lipase (>2.0 x ULN) | 1% | 2% | <1% | <1% |
| Triglycerides (>750 mg/dL) | 9% | 6% | 10% | 8% |

^1^ ULN=Upper Limit of Normal

Hepatitis B

Exacerbations of HBV have been reported in patients after discontinuation of FTC. Patients, who are coinfected with HBV, may have increased values on liver function tests and exacerbation of hepatitis symptoms when FTC is stopped. Usually these symptoms are self-limiting; however, serious complications have been reported. The causal relationship to FTC discontinuation is unknown. Patients coinfected with HBV and HIV should be closely monitored with both clinical and laboratory follow-up for several months after stopping FTC treatment.

Additional information can be found in the most recent Emtriva package insert.

2.2.4 Tenofovir Disoproxil Fumarate (TDF, Viread®)

Tenofovir disproxil fumarate (TDF), (9-[(R)-2-[[bis[[(isopropoxycarbonyl)oxy] methoxy]phosphinyl]methoxy]propyl] adenine fumarate (1:1)) (formerly known as PMPA prodrug or GS-4331-05) is approved for the treatment of HIV-1 infection. TDF is an orally bioavailable prodrug of tenofovir, an acyclic nucleotide analogue with activity in vitro against retroviruses, including HIV-1 and HIV-2, and against hepadnaviruses. TDF is metabolized intracellularly to the active metabolite, tenofovir diphosphate (PMPApp), which is a competitive inhibitor of HIV-1 reverse transcriptase that terminates the growing DNA chain. Although TDF is a nucleotide analogue, it has the same mechanism of action and resistance pattern as NRTIs. Therefore, for simplification of discussion, TDF will be referred to as an NRTI in this study.

Pre-Clinical

The in vitro antiviral activity of tenofovir against laboratory and clinical isolates of HIV-1 was assessed in lymphoblastoid cell lines, primary monocyte/macrophage cells and peripheral blood lymphocytes. The IC50 (50% inhibitory concentration) values for tenofovir were in the range of 0.04 µM to 8.5 µM. In drug combination studies of tenofovir with nucleoside reverse transcriptase inhibitors (abacavir, didanosine, lamivudine, stavudine, zalcitabine, zidovudine), non-nucleoside reverse transcriptase inhibitors (delavirdine, efavirenz, nevirapine), and protease inhibitors (amprenavir, indinavir, nelfinavir, ritonavir, saquinavir), additive to synergistic effects were observed. Tenofovir displayed antiviral activity in vitro against HIV-1 clades A, B, C, D, E, F, G and O (IC50 values ranged from 0.5 µM to 2.2 µM) (Viread Product Information, 2003).

Following oral administration of a single dose of TDF 300 mg to HIV‑infected patients in the fasted state, TDF is rapidly converted to tenofovir. Maximum tenofovir concentrations are observed in serum within one hour of dosing in the fasted state (Viread Product Information, 2003). Intracellularly, tenofovir is metabolized to its active metabolite, tenofovir diphosphate, by constitutively expressed cellular enzymes through two phosphorylation reactions. Because tenofovir is a nucleotide analogue, it does not require the initial phosphorylation reaction of nucleoside analogues. The median terminal elimination half-life of tenofovir is approximately 17 hours and steady‑state is achieved in approximately 3 to 4 days. Tenofovir diphosphate has a prolonged intracellular half-life ranging from 12 to 50 hours in activated and resting peripheral blood mononuclear cells (PBMCs) [Robbins, 1998].

Clinical Experience

Study 917 demonstrated that ARV‑naive subjects experienced a median decrease of −1.6 log_10_ copies/mL in HIV RNA when treated with Viread 300 mg once daily as monotherapy for 21 days. [Louie, 2002].

Efficacy in treatment-experienced subjects was shown in study 907, a randomized, 48 week, double-blind, placebo-controlled, phase 3 study. Five hundred fifty‑two treatment-experienced subjects with 5.4 years of prior ART and extensive nucleoside resistance at baseline received either TDF 300 mg or placebo through 24 weeks, followed by open-label TDF for the remaining 24 weeks. At baseline, mean (± SD) HIV‑1 RNA levels were 3.35 (± 0.52) log_10_ copies/mL in the TDF group vs. 3.38 (± 0.49) log_10_ copies/mL in the placebo group. For the primary efficacy endpoint (time-weighted average change from baseline to week 24 in HIV‑1 RNA, DAVG_24_), the TDF group demonstrated a significant change in HIV‑1 RNA compared with the placebo group (−0.61 and −0.03 in log_10_ copies/mL, respectively; p <0.001). Following 48‑weeks of treatment, the tenofovir DF group had a mean time‑weighted average change from baseline in viral load of −0.57 log_10_ copies/mL. Subjects who crossed over to 24 weeks of TDF after receiving placebo demonstrated a DAVG_24_ of −0.60 log_10_ copies/mL at week 48. At week 24, the mean change in HIV-1 RNA was similar to the DAVG results, with the tenofovir DF group demonstrating a significant change in HIV-1 RNA compared with placebo (−0.59 and −0.01 log_10_ copies/mL, respectively; p < 0.001). This antiviral response was sustained through week 48 with a mean change of −0.53 log_10_ copies/mL. Notably, subjects who began TDF at week 24 after receiving placebo demonstrated a mean change in HIV-1 RNA of −0.64 log_10_ copies/mL at week 48. Importantly, through 24 weeks, the rate of Grade 3 and 4 clinical adverse events during the double-blind phase was similar between the two groups (13% in the TDF group and 14% in the placebo group). Through 48 weeks, the frequency of Grade 3 or 4 clinical adverse events in the subjects randomized to tenofovir DF increased to 20% (Squires, 2003).

Study 903 is an ongoing trial, with a 144-week randomized, double‑blind trial designed to compare the efficacy and safety of a treatment regimen of TDF, 3TC, and EFV to a regimen of d4T, 3TC and EFV in 600 ARV-naive subjects with HIV infection. Following the completion of the double blind portion of the trial, there is an additional 2 year single arm open label portion of the trial in selected sites, wherein all patients receive TDF, 3TC and EFV as once daily regimen. (Patients originally randomized to the d4T arm switch to receive TDF.)

In a preliminary 144-week analysis, when missing observations in the ITT population were treated as having plasma HIV‑1 RNA concentrations greater than 400 copies/mL, 76% of subjects in the TDF group and 72% of subjects in the d4T active control group achieved plasma HIV‑1 RNA concentrations <400 copies/mL. Plasma HIV‑1 RNA concentrations <50 copies/mL at week 144 were seen in 73% and 69% of subjects in the TDF and d4T active control groups, respectively. The mean increases in CD4+ cell count from baseline to week 144 were 263 and 283 cells/mm^3^ for the TDF and d4T active control groups, respectively. The assessment of safety and tolerability indicate that the safety profile of TDF 300 mg/day was similar to that of the d4T active control. [Gallant, 2004].

Safety Profile

More than 1,200 patients have received TDF 300 mg once daily alone or in combination with other ARVs in phase I‑III clinical trials. Over 11,000 patients have received TDF in expanded access programs. The cumulative patient exposure to marketed TDF from first approval to 31 December 2003 is estimated to be approximately 200,000 patient-years of treatment.

In clinical trials in treatment-experienced patients (Studies 902 and 907), the safety profile of TDF 300 mg/day was similar to that of placebo. There were no clinically significant adverse events attributable to TDF 300 mg once daily other than a slightly higher incidence of mild to moderate gastrointestinal adverse events (nausea, diarrhea, vomiting and flatulence). Few adverse laboratory events were documented other than mild or moderate transient hypophosphatemia. Clinically significant events considered by the investigator to be related to TDF were uncommon and none suggested potential adverse drug reactions or drug-drug interactions [Schooley, 2002; Squires, 2003].

Study 910 was initiated to observe the long-term safety effects of TDF, in combination with other ARVs, in subjects who have completed prior TDF studies 901, 902, and 907. The long-term safety and tolerability of TDF were monitored using periodic assessments of concomitant medications, AEs, serial laboratory tests, and bone densitometry (in select subjects). A total of 687 subjects received TDF 300 mg either initially or through rollover. Long‑term follow up shows that the incidence of adverse events or laboratory abnormalities leading to discontinuation of TDF remained low despite mean treatment duration of more than two years, and extending to nearly four years in some subjects. None of the AEs or laboratory abnormalities that led to study drug discontinuation had a reported incidence of more than 1%. Furthermore, there is no indication of nephrotoxicity in this highly treatment-experienced population [Cheng, 2003].

In study GS-99-903 (N=600) {3960}, tenofovir DF in combination with lamivudine (300 mg/day) and efavirenz (600 mg/day) was at least as well tolerated as a stavudine-based control regimen during 144 weeks of treatment. With the exception of dizziness, the profile of frequent adverse events was similar to that in treatment-experienced patients. Most adverse events were of mild or moderate severity. The incidence of hypophosphatemia, creatinine elevations, nausea, diarrhea, vomiting and flatulence was similar in both treatment arms [Gallant, 2004].

Laboratory Abnormalities

Laboratory abnormalities observed in these studies occurred with similar frequency in the TDF and placebo-treated groups. In study 903, AE rates were similar between the d4T/3TC/EFV and TDF/3TC/EFV arms, although cholesterol levels and triglyceride levels were generally higher in the d4T-containing arm.

In rare cases, hypophosphatemia, proteinuria, glycosuria, and reduced creatinine clearance have been seen, and three cases of renal tubular injury have been reported [Reynes et al., Harris et al.]. TDF is effective in reducing HBV in coinfected patients [Nunez et al.].

TDF should not be used with two NRTIs as a triple ARV therapy when considering a new treatment regimen, given poor results when combined with abacavir (ABC) and 3TC or ddI and 3TC for naïve patients (Gilead communication).

Tenofovir and TDF administered in toxicology studies to rats, dogs, and monkeys at exposures (based on AUCs) between 6- and 12-fold higher than observed in humans caused bone toxicity. In monkeys, the bone toxicity was diagnosed as osteomalacia, and appeared to be reversible upon dose reduction or discontinuation of tenofovir. In rats and dogs, the bone toxicity manifested as reduced bone mineral density. The mechanism(s) underlying bone toxicity is unknown. It is not known whether long-term administration of TDF (≥1 year) will cause bone abnormalities. Therefore, appropriate consultation should be sought if bone abnormalities are suspected.

Hepatitis B Virus (HBV)

Exacerbations of HBV have been reported in patients after discontinuation of TDF. Patients, who are coinfected with HBV, may have increased values on liver function tests and exacerbation of hepatitis symptoms when TDF is stopped. Usually these symptoms are self-limiting; however, serious complications have been reported. The causal relationship to TDF discontinuation is unknown. Patients coinfected with hepatitis B (HBV) and HIV should be closely monitored with both clinical and laboratory for several months after stopping TDF treatment.

Bone Toxicity

In study 903 through 48 weeks, decreases from baseline BMD were seen at the lumbar spine and hip in both arms of the study. The proportion of patients who met a protocol-defined value of BMD loss (5% decrease in spine or 7% decrease in hip) was higher in the TDF group than in the d4T group. In addition, there were significant increases in levels of four laboratory parameters of bone metabolism (serum bone-specific alkaline phosphatase, serum osteocalcin, serum C-telopeptide, and urinary N-telopeptide) in the TDF group compared with the d4T group, suggesting increased bone turnover. Serum parathyroid hormone levels were also higher in the TDF group. There was one bone fracture reported in the TDF group compared with four in the d4T group; no pathologic fractures were identified over 48 weeks of study treatment. The clinical significance of changes in the BMD and the biochemical markers is unknown, and follow-up is continuing to assess long-term impact.

Further information can be found in the TDF Investigator Brochure and the most recent Viread package insert.

2.2.5 Emtricitabine and Tenofovir Disoproxil Fumarate Fixed Dose Combination Tablet (FTC/TDF, Truvada®)

Gilead Sciences has developed Truvada, a new product containing FTC 200 mg and TDF 300 mg in a fixed-dose combination (FDC) tablet formulation. A New Drug Application (NDA) for the FDC was filed with the U.S. FDA on March 12, 2004 and was approved on August 2, 2004. As a component of the NDA, two phase I studies evaluating the pharmacokinetics of co-administered FTC and TDF tablet formulation have been completed.

Clinical Pharmacokinetics

Study FTC-114 was a 21-day, open-label, randomized, three-way crossover study to evaluate the steady-state pharmacokinetics of FTC (200 mg capsules given once-daily for 7 days) and tenofovir (administered as TDF tablets 300 mg once-daily for 7 days) when administered alone and together in healthy volunteers [Blum, 2003]. A total of 19 healthy volunteers received in a randomized order each of the following three 7-day treatments over a 21-day treatment period; drug was administered 30 minutes after a standardized breakfast on days 1, 5, 6, and 7 of each treatment period:

-Treatment A: FTC 200 mg QD x7 days

-Treatment B: TDF 300 mg QD x 7 days

-Treatment C: FTC 200 mg + TDF 300 mg QD x 7 days.

Plasma pharmacokinetic parameters at steady-state were assessed over a 24 hour dosing interval following the last dose of each treatment using a non-compartmental analysis. Results based on 17 subjects who completed the study showed that FTC had no effect on the pharmacokinetics of tenofovir when administered with TDF on the basis of statistical analyses of steady state AUC, C_max_, and C_min_ values. Tenofovir DF also had no clinically significant effect on the pharmacokinetics of FTC on the basis of AUC, C_max_, and C_min_ values. Although C_min_ of FTC increased approximately 20% with TDF co-administration, AUC and C_max_ were not affected. FTC and TDF were generally well tolerated when administered alone or together for periods of up to 7 days in this healthy volunteer population.

Bioequivalence and Food Effect

Study GS-US-104-172 was a phase 1, 28-day, randomized, four-way crossover, pharmacokinetic study in healthy volunteers designed to evaluate the bioequivalence of the FTC/TDF combination tablet compared to the FTC capsule and TDF tablet administered concurrently and also the effect of food (high-fat meal and light meal) on pharmacokinetics [Kearney, 2004]. A total of 44 healthy volunteers received in a randomized order each of the following single treatments either in a fasted state, high-fat meal or light meal over a 28-day treatment period; drug was administered on days 1, 8, 15, and 22 with a 7-day washout period separating study treatments:

-Treatment A: Single 200 mg FTC capsule co-administered with a single 300 mg TDF tablet, under fasting conditions

-Treatment B: FTC 200 mg/TDF 300 mg combination tablet administered under fasting conditions

-Treatment C: FTC 200 mg/TDF 300 mg combination tablet administered under fed (high-fat meal) conditions

-Treatment D: FTC 200 mg/TDF 300 mg combination tablet administered under fed (light meal) conditions.

Plasma pharmacokinetic parameters at steady-state were assessed over a 48 hour dosing interval following the last dose of each treatment using a non-compartmental analysis. Results based on 39 subjects who completed the study revealed the ratios for both the rate and extent (C_max_ and AUC) of tenofovir bioavailability after its administration as TDF or as the combination tablet were contained within the bounds of 80% and 125%, demonstrating bioequivalence of tenofovir between the two treatments. Similarly, bioequivalence was demonstrated between the FTC capsule and the FTC/TDF combination tablet. The 90% confidence intervals for the geometric least squares means ratios of FTC C_max_ and AUC for FTC capsules and the combination tablet were contained within the 80% and 125% interval [Kearney, 2004].

Administration of the FTC/TDF combination tablet after either a high-fat or a light meal was associated with a delay in the time to maximum plasma tenofovir concentration (T_max_) relative to T_max_ for the fasted state. Intake of a high-fat meal (784 kcal and 58% fat) or a light meal (373 kcal and 20% fat) resulted in an altered pharmacokinetic profile for tenofovir. The C_max_ of tenofovir increased by approximately 16% and 13.5%, respectively, compared with the fasted-state administration. Likewise, an increase of approximately 35% or 34% in tenofovir AUC_0-∞_ was observed after administration with a high-fat or light meal, respectively, compared with the fasted state. FTC pharmacokinetic parameters after ingestion of food (high-fat or light meal) were essentially the same as those for the fasting state, and the 90% confidence intervals for the ratios of treatment geometric means for C_max_ and AUC_0_-∞ after either a high-fat or light meal were contained within 80% to 120%, indicating no food effect on the pharmacokinetic profile of FTC.

Overall, Study GS-US-104-172 demonstrated bioequivalence between the FTC/TDF combination tablet and the FTC capsule and TDF tablet formulations when administered separately. Administration of the FTC/TDF combination tablet with either a high-fat meal or light meal increased tenofovir exposure by approximately 30% compared with fasted-state administration. Clinical experience with TDF indicates that the effect of food on tenofovir exposure is not of clinical relevance. FTC and TDF, either administered as a combination tablet (containing FTC 200 mg/ TDF 300 mg) or co-administered as FTC 200 mg capsule and TDF 300 mg tablet were well tolerated.

Clinical Experience

Study M02-418 was a Phase III, randomized, open-label, multicenter study designed to compare lopinavir 800 mg/ritonavir 200 mg QD vs. lopinavir 400 mg/ritonavir 100 mg BID with the background regimen of FTC 200 mg QD and TDF 300 mg QD in ARV-naïve patients with HIV-1 RNA >1000 copies/mL [Gathe, 2004; Molina, 2004; Podzamczer, 2003]. A total of 190 patients, between the ages of 19 and 75 years were enrolled; 115 to the QD arm and 75 to the BID arm. At Week 48, based on the ITT (NC=F) analysis, 70% of patients in the QD regimen demonstrated HIV-1 RNA <50 copies/mL, compared to 64% of those in the BID group (95% CI: -7%; 20%). In addition, increase in CD4+ cell counts was similar between the 2 groups. Resistance testing results were available in 15 patients at Week 48; 8 in the QD group and 7 in the BID group. Genotypic analysis did not identify any LPV or TDF resistance mutations. Resistance to FTC was identified in a total of 3 patients (2 in the QD group and 1 in the BID group) [Molina, 2004].

Gastrointestinal adverse events were the most common cause for discontinuation. Overall, the most common AEs (>3%) reported were diarrhea, nausea, and vomiting, with diarrhea being reported significantly higher in the QD group (16% vs. 5%; p=0.04). The most common Grade 3/4 laboratory abnormalities (>3%) reported were increased ALT (>5 x ULN), AST (>5 x ULN), triglyceride (>750 mg/dL), and amylase (>2 x ULN) levels; no significant differences between the 2 groups were observed [Gathe, 2004].

Study 934 is a phase III, randomized, open-label, multicenter study designed to compare a regimen of EFV with either TDF 300 mg + FTC 200 mg QD or ZDV 300 mg/3TC 150 mg BID as FDC Combivir [Gazzard, 2004]. A planned 24 week interim analysis was presented showing that discontinuation occurred more frequently in the ZDV/3TC group than TDF + FTC, mostly because of adverse events such as anemia and nausea. The 24 week data demonstrated that using the time to loss of virologic failure primary analysis that the proportion of subjects with plasma HIV-1 RNA levels less than 50 copies/mL was 73% in the TDF + FTC group compared to 65% in the ZDV/3TC-treated subjects (p=0.038). Virologic failure was uncommon in both groups with genotype data limited to 10 subjects on ZDV/3TC and 8 on TDF + FTC, with similar resistance patterns between the study groups, mostly wild-type, M184V/I, NNRTI resistance or both M184V/I and NNRTI resistance with no subjects developing the K65R mutation.

Further information can be found in the most recent Truvada package insert.

2.2.6 Zidovudine (ZDV, Retrovir®)

ZDV is generally well tolerated. The major AEs include headache, fatigue, malaise, nausea, anemia, and neutropenia. Long-term ZDV therapy is associated with myopathy and rare cases of steatosis with hepatic failure and death.

Additional information can be found in the most recent Retrovir package insert.

2.2.7 Didanosine (ddI, ddI-EC, Videx®)

Didanosine is an acid-labile antiretroviral NRTI; the tablet formulation of ddI includes antacids to prevent acid hydrolysis of the drug in the stomach. In most studies, the 400-mg QD dose has demonstrated both pharmacokinetic and virologic equivalence and similar toxicity rates to the 200-mg BID dose through week 24. Results from study AI454-148, however, indicated an inferior virologic response at 48 weeks in patients on a regimen of ddI, ZDV, and NFV compared with patients on a regimen of ZDV, 3TC, and NFV. The proportions of patients with HIV-1 RNA <400 copies/mL were 50% and 59%, respectively, while the proportions of those with HIV-1 RNA <50 copies/mL were 34% and 47%, respectively. CD4+ cell counts were comparable between the two arms. The most common AEs associated with ddI are gastrointestinal upset, peripheral neuropathy, and pancreatitis.

In addition, an enteric-coated (EC) capsule of ddI is available. The capsule does not require the buffering used in the tablet formulation. Pharmacokinetic studies comparing the EC capsule to the buffered tablets indicate that the AUCs are equivalent, the C_max_ of the EC formulation is 60% of the C_max_ of the tablets, and the median T_max_ values are 2.33 hours for the EC formulation and 0.67 hours for the tablets (Bristol-Myers Squibb communication/ Videx package insert). Gamma scintigraphy indicates the delay in T_max_ is related to the time needed to dissolve the capsule in the stomach, and that absorption is rapid once the drug enters the small intestine. In healthy volunteers, following a high-fat meal, mean AUC was reduced 19%, C_max_ was reduced 46%, and T_max_ was increased from 2.00 hours (fasting) to 5.25 hours (fed). Therefore, the same restrictions on food intake apply to the EC capsules as to the tablets. Overall, participants on the EC formulation have experienced similar rates of gastrointestinal toxicity (primarily diarrhea) as those on tablets.

Clinicians should use caution when coadministering TDF, ddI EC, and either NVP or EFV in treatment-naïve HIV patients with high baseline viral loads. Results from two recently conducted, investigator-sponsored trials by Podzamczer et al. (the ININ Study) and Gatell (J.M. Gattell’s written communication to BMS) have demonstrated a potential for early virologic failure associated with this antiretroviral regimen in treatment-naïve HIV patients with high baseline viral loads. The ININ Study [Podzamczer, 2004] is an open-label, randomized, multicenter pilot study with a planned enrollment of 50 treatment-naïve HIV patients designed to assess efficacy and safety of TDF 300 mg once daily + ddI EC 250 mg once daily (< 60 kg: 200 mg once daily) + EFV 600 mg once daily compared with TDF 300 mg once daily + ddI EC 250 mg once daily (< 60 kg: 200 mg once daily) + EFV 600 mg once daily + LPV/RTV 400/100 mg twice daily. Of the 36 enrolled patients, 26 were available for follow-up at 3 months. Six of 14 patients (42.8%) in the TDF + ddI EC + EFV arm experienced protocol-defined virologic failure, versus 0 of 12 patients in the TDF + ddI EC + EFV + LPV/RTV arm. Baseline viral load >100,000 copies/mL and advanced stage of disease (CD4+ cell count <200 cells/mm^3^ plus CDC stage C or B3) were seen in all six patients with virologic failure but in none of the eight patients without virologic failure. Resistance patterns that included G190E/S (n=3), L74V/I (n=4), and K65R (n=2) mutations were observed at failure. A retrospective database analysis of 5000 treatment-naïve HIV patients in whom therapy was initiated between October 2002 and March 2004 was performed (Gatell, written communication to BMS). Fourteen patients were identified as having received a regimen of ddI EC 250 mg once daily and TDF 300 mg once daily, plus either EFV 600 mg once daily (n=10) or NVP 400 mg once daily (n=4). After 12 weeks of therapy, 5/14 patients (36%) experienced suboptimal (plasma viral load drop <2 log_10_ copies/mL) response rates. Two additional patients (total 7/14, 50%) who were treatment-responders at Week 12 reached protocol-defined virologic failure at Week 24. The seven cases of virologic failure consisted of 2/4 patients receiving NVP- and 5/10 patients receiving EFV-containing regimens. At baseline, virologic failure patients had a median log_10_ viral load of 5.8 (range, 4.7-6.0) copies/mL and a median CD4+ cell count of 126 (range, 24-281) cells/mm^3^. The mechanism of early virologic failure in these patients is unclear. Further investigations are ongoing to better understand the clinical implications of these results.

Early virologic failure appears to be limited to the specific combination of TDF + ddI EC + either EFV or NVP as there are data from registrational trials supporting the efficacy of EFV and TDF-based regimens as well as EFV and ddI EC-based regimens in treatment-naïve HIV patients (Viread and Emtriva package inserts) [Saag, 2004].

Additional information can be found in the most recent Videx package insert.

2.2.8 Efavirenz (EFV, Sustiva®, Stocrin®)

EFV is a potent NNRTI that is approved by the FDA and widely used in combination with other antiretroviral agents for the treatment of HIV-1 infection. Studies support its use for initial therapy as well as for salvage therapy.

Clinical Experience

A phase III study (DMP 266-006) compared the safety and efficacy of ZDV (300 mg BID) plus 3TC (150 mg BID) plus EFV (600 mg QD), EFV plus IDV (800 mg Q8h), and ZDV plus 3TC plus IDV. IDV was administered in a fasted state [Staszewski, 1999]. A total of 1266 participants who were EFV-, NNRTI-, PI-, and 3TC-naïve at study entry were enrolled. The mean baseline CD4+ cell count was 341 cells/mm^3^, and the mean baseline HIV-1 RNA level was 4.78 log_10_ copies/mL. At 48 weeks, the proportion of participants with HIV-1 RNA levels <400 (< 50) copies/mL was 68% (62%) for the EFV/NRTI group, 55% (49%) for the EFV/IDV group, and 49% (43%) for the IDV/NRTI group. No differences in CD4+ cell count changes were noted among the groups. During long-term follow-up, the proportion of participants with HIV-1 RNA <50 copies/mL at 72 weeks

was 58% for the EFV/NRTI group, 42% for the EFV/IDV group, and 36% for the IDV/NRTI group.

Recently presented results of ACTG 384 [Robbins, 2002] confirmed that the regimen of EFV with ZDV/3TC was more potent and more durable than a single PI-containing regimen (NFV with ZDV/3TC) and comparable to a four-drug regimen (EFV, NFV with ZDV/3TC).

Safety Profile

The most significant AEs observed in participants receiving EFV were nervous system symptoms, psychiatric symptoms, and rash. Fifty-three percent of participants noted central nervous system (CNS) complaints. CNS symptoms usually begin during the first 1 or 2 days of therapy and generally resolve after the first 2-4 weeks. Dosing at bedtime improves the tolerability of these symptoms and is recommended during the first weeks of therapy and in patients who continue to experience these symptoms.

Serious psychiatric AEs have been reported including severe depression, suicidal ideation or attempts, aggressive behavior, paranoid reactions, and manic reactions. Rash is usually mild to moderate and occurs within the first 2 weeks of initiating therapy. In most participants, rash resolves with continuing EFV therapy within 1 month.

Malformations have been observed in fetuses from EFV-treated monkeys that received doses resulting in plasma drug concentrations similar to those in humans given 600 mg/day. Therefore, pregnancy should be avoided in women receiving EFV, and barrier contraception should always be used in combination with other methods of contraception (e.g., oral or other hormonal contraceptives).

More information can be found in the most recent Sustiva or Stocrin package insert.

## 2.3 Rationale

- - 1. **Rationale for Steps 1 and 2**

Study Medications

The Step 1 initial treatment regimens in A5208/OCTANE will consist of TDF and FTC, combined with either LPV/RTV or NVP.

The TDF/FTC backbone was chosen for several reasons: 1) Combinations of TDF, FTC, and an NNRTI or PI are extremely potent and have been associated with substantial virologic response rates in several studies in treatment-naïve patient populations. 2) TDF and FTC can be taken QD, which is likely to enhance adherence. 3) TDF should be well-tolerated; it is less likely to be associated with anemia than ZDV-based regimens and less likely to be associated with facial wasting and/or lipid metabolism dysregulation than d4T-based regimens. Although ZDV- and d4T-based regimens are currently more widely available in settings in which this study will be undertaken, it is anticipated that TDF will be readily available in the near future and experience with it in settings where ZDV- and d4T-associated resistance mutations are being selected will be critical. Gilead announced in April 2003 the Gilead Access Program, which will provide access to TDF at no profit in every country in Africa and in 15 additional countries in other parts of the world classified as "least developed" by the United Nations (UN). It will be important to gain clinical experience with TDF as this drug becomes more available in the developing world.

WHO guidelines acknowledge that PIs are an accepted standard of care as part of first-line ART regimens, and that “advantages of PI-based regimens …are proven clinical efficacy and well described toxicities” [WHO, 2003]. Disadvantages include cost, heat instability (with certain PIs), and drug interactions with medications such as rifampin, which is used in tuberculosis (TB) treatment regimens. If, however, a PI-based regimen is shown to be of significantly superior efficacy than an NNRTI-based regimen, particularly among women who previously took SD NVP, then PIs may replace NNRTIs in first-line regimens for at least a subset of persons requiring ART in developing nations. Since late 2002, as significant strides have been made in price reductions for several PIs, these agents are becoming more readily available. LPV/RTV has recently been chosen by the government national ART programs of both Botswana and South Africa to become the PI of choice on their formularies. With increasing documentation of NNRTI resistance in the HIV-infected population in the United States [Little, 2002], which is likely to follow elsewhere with increasing use of NNRTIs, it will be critical to develop experience with PIs in settings where NNRTI resistance is on the rise. Of the available PIs, atazanavir (ATV) and LPV/RTV are the two that would seem particularly suited to use in resource-limited settings (RLS). Other currently available PIs are disadvantaged by pharmacokinetic properties, including the need to boost with RTV and sub-optimal treatment responses. ATV has the further advantage of being much less sensitive to ambient heat than other PIs, thus reducing a logistical challenge in settings where access to refrigeration is uncertain. LPV/RTV was chosen over ATV because of an unexplained drug interaction between ATV and TDF resulting in approximately 40% less exposure to ATV than when it is used in non-TDF containing regimens. Were ATV to be used in this study, it would require boosting with non-co-formulated RTV adding unwarranted complexity.

NNRTIs are included in the first-line ART regimens recommended by WHO in resource-limited settings as well as by the Department of Health and Human Services (DHHS) in the United States. The choice of NNRTI for this study depends on several factors.

The K103N mutation is the most common major NNRTI mutation observed on population sequencing of virus from women 6-8 weeks after receipt of SD NVP (virus from 16 of 18 [89%] women with resistance at this time point had a K103N mutation alone or in combination with one or more other mutations, and 6 of 18 [33%] women harbored virus with detectable Y181C mutations) [Eshleman, Mracna 2001]. Of interest, the pattern of NVP resistance mutations detected after SD NVP appears to depend upon the timing of sample collection. In another study performed by Eshleman, the most common resistance mutation detected from maternal samples taken seven days after SD NVP was Y181C (detected in 13 of 15 [87%] women) compared with K103N (in 6 of 15 [40%] women) [Eshleman, 2003]. The K103N confers high-level resistance to both NVP and EFV, and the Y181C mutation confers high-level resistance to NVP and clinically significant resistance to EFV. It is highly likely that resistance selection with SD NVP will have the same implications for treatment response to either NVP or EFV.

EFV is less often associated with hepatotoxicity than is NVP, although the concern about NVP-related hepatotoxicity is less among patients with a CD4+ cell count <250 cells/mm^3^ at initiation of therapy. Although EFV may be slightly more efficacious than NVP based upon data from observational studies [Cozzi-Lepri et al, 2002; Phillips, 2001], the 2NN study suggested in the context of a large randomized clinical trial (RCT) that EFV and NVP had similar treatment and virologic efficacies [van Leth et al, 2003].

Concerns about fetal anomalies associated with EFV exposure during pregnancy make it critical that other alternatives be available in a population of women who might be contemplating additional pregnancies, as is expected to be the case in this study. Furthermore, NNRTIs may have a negative impact on the efficacy of hormonal contraception, and women cannot always negotiate condom use by their partners even if they do not desire another pregnancy. For these reasons, NVP is the NNRTI of choice among women of childbearing age in most resource-limited settings, and is therefore the most relevant NNRTI to study in this population.

Because a substantial proportion of women who have been previously exposed to SD NVP may experience virologic failure on NVP-containing regimens relatively early in treatment, the study will attempt to provide an effective second regimen for participants for the duration of the study. Women who fail virologically or who experience treatment-limiting toxicity or intolerance with NVP will enter Step 2 and switch to LPV/RTV, and vice versa. Women who must discontinue the Step 1 initial regimen due to virologic failure may also change to available NRTIs as part of a Step 2 second regimen at the discretion of the site investigator. Single or dual NRTI substitutions secondary to virologic failure, toxicity, or intolerance (but without changing LPV/RTV or NVP) will not lead to a step change. These NRTIs may include ZDV or ddI provided by the study, and other NRTIs that may be available outside of the study.

Inclusion of Only Women in the Two Trials

Enrollment in the randomized clinical trial (RCT) among participants previously exposed to SD NVP for prevention of MTCT will, by necessity, be restricted to women. Enrollment in the RCT among non-NVP exposed participants will also be restricted to women, given the differences in toxicities experienced by women versus men with certain antiretrovirals (e.g., NVP), as well as the possibility of differences in adherence or virologic response to ART in women and men. Such differences could complicate the comparison of the relative efficacy of a PI- versus an NNRTI-based regimen in NVP-exposed and non-NVP exposed populations if men were to be enrolled only the NVP-unexposed group.

Pharmacokinetic Sampling in Arm 1A

Women appear to be at increased risk of NVP-induced rash and hepatitis, but the pharmacokinetics of NVP in women, especially those experiencing drug toxicity, has not been well described [Pollard 1998]. A retrospective study of routine therapeutic drug monitoring found that median toxic NVP drug levels (>6 mg/L) occurred more frequently in women than men (57% vs. 40.7%) and that gender was the single predictive factor [LaPorte 2003]. The association of NVP-induced adverse reactions in women with higher NVP plasma exposure are primarily derived from pharmacokinetic studies involving predominately male participants. Causes of higher plasma NVP exposure in women are unknown but might be smaller body mass, reduced drug clearance, or altered metabolism and formation of NVP metabolites.

Pharmacokinetics of NVP

Nevirapine is well absorbed (>93±9%) after oral administration and has a long plasma elimination half-life of 45 to 77 hours after a single dose [Lamson, 1999]. NVP is 60% bound to plasma proteins, primarily albumin. After multiple dosing, plasma concentrations and the area under the plasma concentration versus time curve (AUC) are markedly reduced and the half-life is approximately 24 hours, which is attributed to gut and hepatic enzyme autoinduction by CYP3A4 and CYP2B6. Maximal enzyme induction occurs within 2-4 weeks of multiple dosing. NVP is eliminated primarily in the urine as 2-, 3-, 8-, and 12-hydroxyNVP; less than 3% is excreted in urine as the unchanged parent compound. The major metabolites 2- and 3-hydroxyl NVP are formed by CYP3A4 and CYP2B6, while the major 12-hydroxyNVP and the minor metabolite, 8-hydroxyNVP, are dependent on CYP2B6, CYP2D6, and CYP2C9 [Erickson, 1999; Riska, 1999].

Results of the effects of gender on NVP pharmacokinetics are conflicting and based on limited sample size. Pharmacokinetic data from the manufacturer, obtained in HIV-negative women, indicate that the volume of distribution of NVP is higher in women (1.54 L/kg) compared with men (1.38 L/kg) but that AUC is not different. In ACTG 241, gender significantly correlated with NVP clearance, averaging 3.97 L/hr for men compared with 3.02 L/hr for women, but women were not well represented (10 out of 82 patients) [Zhou 1999]. A pivotal efficacy trial for NVP including 8 women and 35 men demonstrated no significant differences in pharmacokinetics between women and men.

Women appear to be at a greater risk of developing rash than men. A retrospective cohort study of 358 patients who received a NVP-based regimen reported that 15.8% of women developed a rash compared with 8.4% of men (p=0.05) [Bersiff-Matcha 2001]. After adjustments for age and baseline CD4+ cell count in a multivariate analysis, women had a 7-fold increased risk of severe rash. To reduce the occurrence of rash, and considering the induction of drug metabolism that occurs with NVP, the manufacturer recommends an initial NVP dose of 200 mg daily for 2 weeks, followed by dose escalation to the therapeutic dosage of 200 mg twice daily. Rash was further reduced in HIV-infected persons who began a lower than recommended dose escalation regimen (100 mg daily for one week, then increasing by 100 mg/week up to the 400 mg daily dose) compared with the standard manufacturer method [Barreiro, 2000]. Because dose escalation minimizes the occurrence of rash, higher drug exposure to NVP may explain the increased incidence of rash in women.

A direct correlation of NVP-induced hepatotoxicity with drug exposure has been proposed [Nunez, 2003]. Significantly higher NVP trough concentrations (e.g., 6.5 vs. 5.2 mcg/mL) were observed in those (gender unknown) who developed hepatotoxicity. NVP drug concentrations >6 mcg/mL were associated with a 92% risk of liver toxicity [Gonzalez, 2002]. Additionally, higher total and unbound NVP trough levels were weakly associated with higher GT levels (p=0.020, 0.006) but not the ALT level [Almond, 2003]. Gender was predictive of hepatotoxicity in the South African FTC-302 study where 15% of participants developed NVP hepatotoxicity [Sanne, 2000]. The high proportion of women participants (59%) also raises unresolved questions of a gender predisposition. Additional risk factors for NVP-induced hepatotoxicity have included higher baseline CD4+ cell count, co-infection with hepatitis B and/or C, alcohol consumption, wasting, concomitant use of d4T, and baseline liver function abnormalities. The risk of NVP-induced hepatotoxicity may also increase with a longer duration of exposure [Martinez, 2001].

- - 1. **Rationale for Step 3**

**Significant advances have been made since 2000 in rolling out HIV care and ART in RLS. Nevertheless, ART programs continue to face challenges in providing uninterrupted care and treatment to large numbers of patients. Concern has been raised as to the welfare of clinical trials participants who are transitioned to local care and treatment at study end in RLS. This is particularly true when participants need to switch some of their ARVs at study end, as not all study-provided ARVs are available locally. A5208/OCTANE provides an ideal opportunity to document clinical, virologic, and immunologic outcomes among such participants, and to either allay these concerns or identify important areas that deserve future attention when planning and executing clinical trials globally.**

**Furthermore, one of the critical questions related to ART in RLS is the importance and role of routine viral load monitoring (vs. clinical and/or immunologic monitoring) in patients on ART. Viral load monitoring is currently available to only a small proportion of patients receiving ART globally. The vast majority of participants in A5208/OCTANE will have experienced prolonged virologic suppression on ART at the end of Step 1 or 2. The extended follow-up in Step 3 will allow the team to explore whether virologic outcomes, regimen switches, and the emergence of new ARV drug resistance differ between participants receiving care in treatment programs that do and do not conduct routine viral load monitoring. At the time of preparation of Version 4.0 of the A5208/OCTANE protocol, half of the local treatment programs at A5208/OCTANE sites do offer routine viral load monitoring, and half do not. Although the observational data collected during Step 3 will not conclusively answer the question of the role of viral load monitoring, if we do find that a very high proportion of participants with stable virologic suppression experience good clinical and virologic outcomes regardless of viral load monitoring strategy, this will provide valuable data on this important topic.**

**Finally, the vast majority of participants will be able to continue the LPV/RTV or NVP that they are receiving at the time of transitioning from Step 1 or 2 to local care in Step 3. Additional follow-up in Step 3 will help identify whether SD NVP-exposed women treated with NVP in their first regimen continue to experience excess virologic failure compared with those treated with LPV/RTV in first-line; and will provide additional important data regarding responses to second-line treatment.**

**The Step 3 follow-up period of 72 weeks is expected to provide adequate time to evaluate longer-term outcomes following transition to local care and treatment programs. Most local non-study supported treatment programs will see participants every 1-3 months for drug dispensing and clinical visits, while CD4 and viral load monitoring may only occur once every 6 months if done at all. One of the objectives of following A5208 participants is to assess outcomes in the setting of different laboratory monitoring strategies. A 72-week follow-up period will provide the opportunity to assess outcomes in sites with different laboratory monitoring practices after each participant has had 2-3 viral load assessments, either through the study alone or through the study along with locally performed monitoring.**

**Transition to Local Care – Step 3**

**Consenting participants who are on study (on either Step 1 or Step 2) up to 60 weeks after randomization of the last participant will be registered to Step 3 on the same day that they discontinue Step 1 or Step 2. Research sites will refer participants to locally provided non-A5208-supported HIV care and treatment programs/facilities in a timely enough fashion to minimize any interruption in ART. On Step 3, participants will receive HIV treatment including ART from local sources rather than the A5208 study clinic. Non-study ART will start wihin 1 day of discontinuing Step 1 or Step 2. In Step 3, participants will be seen for study visits at the A5208 study clinic (as described below) 12 weeks and again 72 weeks after entering Step 3. It is expected that the majority of participants who have been on NVP and LPV/RTV will continue to have access to these drugs. Ideally, participants should have access to either Truvada or TDF/3TC, but any locally available approved background will be acceptable.**

**Participants who do not wish to participate in Step 3 of the study will also be referred to local care and treatment programs/facilities and will go off Step 1 or Step 2 up to 60 weeks after randomization of the last participant to A5208.**

**On Step 3, following transition to local HIV treatment and care, it is expected that participants will continue to maintain high rates of virologic suppression. The rate of clinical outcomes is not expected to increase, although some toxicities may occur depending on changes in ARVs. If many participants need to transition to ZDV or d4T, higher rates of cytopenia and neuropathy/lactic acidosis, respectively, may be observed. Additionally, "flares" may be experienced by participants with hepatitis B who discontinue TDF. The team hopes that these events will be minimized by advocating for the continuation of the same ARVs in study participants, to the greatest extent possible (and will convey to local treatment clinics their concerns about monitoring for such toxicities when ARV switches do occur).**

**Other expectations include the following:**

- **among participants with stable virologic suppression (i.e., for at least the previous 18 months on Step 1 or 2), the frequency of regimen change and of virologic/genotypic/immunologic/clinical outcomes will be similar at sites that perform routine viral load monitoring compared with sites that do not provide this.**
- **ongoing excess virologic failures in Trial 1 participants who elect to continue NVP will not be seen.**
- **participants in both arms will continue to experience AEs and new diagnoses at a low, constant rate;**
- **participants in the LPV/RTV arm will experience more hyperlipidemia than those in the NVP arm.**

**Participants will be seen 12 weeks and again 72 weeks after entry to Step 3 and will be monitored for toxicity, virologic failure, and signs and symptoms leading to change in ARVs, as described in section 6.0 of the protocol. Between study visits, all monitoring will be performed according to local standard of care (SoC) at local treatment facilities.**

**In order to enhance data completeness and attendance of the week 72 week visit, study staff will have an additional brief telephone conversation, a face-to-face meeting with study participants, and/or will obtain updates from the local care provider/clinic 48 weeks after entry to Step 3.**

**The team realizes that we will not be powered to effectively compare laboratory monitoring strategies in this population, but even a description of the proportion of participants who experience virologic failure and any new genotypic resistance at sites that do not provide plasma HIV-1 RNA monitoring would be valuable.**

# 3.0 STUDY DESIGN

A5208/OCTANE is a phase III study comprising two RCTs to be conducted concurrently. Both trials will compare the virologic response to NNRTI-based (Arm 1A) versus PI-based (Arm 1B) ART in HIV-infected, treatment-naïve women. There will be up to **3** steps in this study. At entry, all participants will be registered to Step 1.

Trial 1 will evaluate the superiority of PI-based ART over NNRTI-based ART in women with prior SD NVP prophylaxis for MTCT of HIV. Trial 2 will evaluate the equivalence of PI- and NNRTI-based ART in women with no prior NVP exposure.

Step 1: Initial Regimen

At entry, **740** HIV-infected, treatment-naïve women, with CD4+ cell count <200 cells/mm^3^ will enter Trial 1 (240 participants) or Trial 2 (**500** participants) based on their prior exposure to NVP. Within each trial, participants will be stratified using their screening CD4+ cell counts as follows: <50 or ≥50 cells/mm^3^. Participants will enter Step 1 and be randomized to Arm 1A or Arm 1B. The regimens will be:

Arm 1A

NVP 200 mg po QD in the AM for 14 days, then NVP 200 mg po BID PLUS

FTC 200 mg po QD PLUS

TDF 300 mg po QD

OR

NVP (as above) PLUS

FTC/TDF 200mg/300mg po QD

OR

Arm 1B

LPV/RTV 400/100 mg po BID PLUS

FTC 200 mg po QD PLUS

TDF 300 mg po QD

OR

LPV/RTV (as above) PLUS

FTC/TDF 200mg/300mg po QD

Study drugs will be provided for all participants through 48 weeks after the final participant is randomized.

Step 2: Second Regimen

Participants on Step 1 who discontinue NVP or LPV/RTV, either because of virologic failure, toxicity, or intolerability, may enter Step 2 only if they switch to LPV/RTV or NVP, respectively. Since participants who meet the protocol definition of virologic failure may have continued benefit from the Step 1 regimen, and/or may have limited second regimen options, switching to a Step 2 regimen will not be **mandatory. Participants may remain on the Step 1 regimen at their own and the site investigator’s discretion despite the occurrence of virologic failure.**

Participants randomized to Arm 1A treatment will switch to:

Arm 2A

LPV/RTV 400/100 mg po BID PLUS

2 or more NRTIs

Participants randomized Arm 1B treatment will switch to:

Arm 2B

NVP 200 mg po QD for 14 days, then NVP 200 mg po BID PLUS

2 or more NRTIs

Choice of NRTIs is at the discretion of the site investigator and may include any potentially viable combination of the study-provided drugs used in Step 1, and/or additional NRTIs provided by A5208/OCTANE, and/or non-study drugs not available through A5208/OCTANE.

The following drugs will be available through A5208/OCTANE: NVP, LPV/RTV, FTC, TDF, FTC/TDF, ddI, EFV, and ZDV.

Development of tuberculosis (TB) while on study

Participants who develop TB and need rifampin-containing TB treatment while on study may be offered EFV (800 mg po QD) in place of NVP or LPV/RTV (based on assigned treatment) if they can use appropriate contraception (as outlined below). NVP (unchanged dose) will be used in participants of childbearing potential who cannot use contraception that would allow treatment with EFV. All participants on TB treatment may continue to receive FTC and TDF or FTC/TDF.

These study drug changes will be made available for the duration of the rifampin-based TB treatment, and for 30 days after stopping rifampin. Thereafter, the participant will return to her assigned study drug regimen.

NOTE: Participants who are participating in sexual activity that could lead to pregnancy and who are receiving EFV must agree to use two reliable methods of contraception, including a reliable barrier method of contraception together with another reliable form of contraception while receiving EFV and for 6 weeks after stopping EFV (see section 5.0).

**Step 3: Transition to Local Care**

**Participants who are still on study at the end of Step 1 or 2, regardless of whether they are still on ART, may be registered to Step 3 and be followed for an additional 72 weeks.**

**Entry to Step 3 will occur at the last study visit for Steps 1 and 2. Ideally, the final visit for Step 1 or 2 will be the first q12 week visit scheduled after June 4, 2009. However, if the site has not yet registered to Version 4.0 of the protocol by the time of this visit, then the participant will remain on study and will return for the combined final Step 1 or 2 visit and Step 3 entry visit as early as possible following the site’s registration to Version 4.0 and before August 27, 2009. No ingestion of study-provided ARVs will occur after the last day on Step 1 or Step 2.**

**At entry to Step 3, participants will be transitioned to** **locally available ARVs and to local clinical care. It is expected that sites will work with local non-A5208-supported treatment facilities to attempt to maintain participants on the same regimen, including the NRTI backbone, beginning at entry to Step 3. Sites will also work ahead of time with local treatment clinics to ensure that participants will have an uninterrupted supply of ARVs as they transition off study ARVs and onto locally-provided ARVs.**

**At week 12 of Step 3, participants will return to the study clinic for a follow-up visit. At week 48, staff will contact participants by phone (or by home/work or other face-to-face visit, if permission for this has been given) to determine vital status, major interim clinical events, ARV regimen changes, and to remind the participant of the week 72 visit. No examination or laboratory testing will be performed at week 48.**

**At week 72 of Step 3, participants will be asked to return again for a follow-up visit.**

**If participants are unable to be contacted or cannot be seen in the study clinic for any of these visits/contacts, then the site is expected to collect as much information as possible either via phone contact with the participant or from other sources including available clinic records (see Step 3 Clinic Visit/Chart Review in section 6.3.4 under Clinical Assessment).**

Pregnancy

Participants who become pregnant while on study and choose to remain on the study will be followed on study through study end. Participants who deliver a child while on-study will be counseled regarding the risks and benefits of and the alternatives to breast-feeding. Women who choose to breast-feed may continue on study.

Pregnant or breast-feeding participants may substitute ZDV for TDF, but will continue to receive FTC and either NVP or LPV/RTV, based on assignment at time of pregnancy. At the completion of pregnancy and breast-feeding, participants will discontinue ZDV and restart TDF. Women who cannot tolerate ZDV may instead take another NRTI (including NRTIs provided by the study or other NRTIs that are locally available) such as ddI, d4T, or TDF during their pregnancy and while breast-feeding, with the approval of the site investigator.

HIV ARV Resistance Testing

At visits indicated in the schedule of events, plasma samples will be collected for possible ARV drug resistance analysis using the ViroSeq HIV-1 Genotyping System. This FDA-cleared system is based on population (bulk) sequencing.

Plasma samples from each participant will also be stored for possible HIV ARV resistance testing using more sensitive methods specifically designed to detect minority variants of HIV-1 with ARV drug resistance mutations. Some of these methods will also be used to evaluate the genetic relatedness of HIV minority variants present at baseline with HIV variants selected at failure in participants receiving NNRTI and/or PI-based regimens.

Since all antiretroviral resistance testing performed in A5208/OCTANE will be done retrospectively, the results of these tests will not be available to guide Step 2 drug selection. If antiretroviral resistance testing is obtained outside of A5208/OCTANE, site investigators may use this information to guide the choice of Step 2 regimens.

Pharmacokinetic sampling in Arm 1A

Participants randomized to Arm 1A in Step 1 will have a plasma sample collected 14 days and 28 days after randomization. Each participant will be asked to report the time of her last dose and to have maintained a medication diary for the previous 72 hours.

# 4.0 SELECTION AND ENROLLMENT OF PARTICIPANTS

The following criteria apply to Trial 1 and Trial 2 unless otherwise noted.

## 4.1 Inclusion Criteria for Step 1

4.1.1 HIV-1 infection, documented by a rapid HIV test or any licensed ELISA test kit, and confirmed by either an ELISA, an IFA, a Western blot, or plasma HIV-1 RNA at the study-associated, DAIDS-approved laboratory. Discordant

confirmatory results should be followed by plasma HIV-1 RNA determination at a DAIDS-approved laboratory for quality control purposes.

4.1.2 Women age ≥ 13 years, or who have attained the minimum age of consent, as defined by the local IRB, whichever is greater.

4.1.3 CD4+ cell count <200 cells/mm^3^ obtained within 90 days prior to study entry from any DAIDS-approved laboratory.

- - 1. For participants in Trial 1, prior SD NVP MTCT prophylaxis.

NOTE: Receipt of SD NVP more than once for any given pregnancy or in >1 pregnancy is not exclusionary.

- - 1. For participants in Trial 1, documentation of all prior SD NVP MTCT prophylaxis.

NOTE: Documentation of each course of prophylaxis may be the current participant report, post partum participant report, documentation of observed dose, results of post partum plasma, or any combination of the above.

4.1.6 For participants in Trial 1, the last SD NVP MTCT prophylaxis course must have been completed at least 6 months prior to study entry.

4.1.7 Plasma HIV-1 RNA quantitation using the Roche Amplicor HIV-1 Monitor Assay (version 1.5, standard not ultra sensitive) within 45 days prior to study entry from any DAIDS-approved laboratory.

4.1.8 The following laboratory values obtained within 45 days prior to study entry:

- Absolute neutrophil count (ANC) ≥750/mm^3^
- Hemoglobin ≥7.0 g/dL
- Platelet count ≥50,000/mm^3^
- AST (SGOT), ALT (SGPT), and alkaline phosphatase ≤2.5 x ULN
- Total bilirubin ≤2.5 x ULN

4.1.9 Evidence of normal renal function within 45 days prior to study entry as determined by an estimated creatinine clearance of ≥60 mL/min using the formula:

{[140 - age(yr)]x [weight(kg)]÷[72 x serum Cr(mg/dL)]} x 0.85

4.1.10 For participants of reproductive potential (defined as girls who have reached menarche or women who have not been post-menopausal for at least 24 consecutive months, i.e. who have had menses within the preceding 24 months), or have not undergone surgical sterilization (e.g. hysterectomy, or bilateral oophorectomy, salpingotomy, or tubal ligation) must have a negative serum or urine pregnancy test within 45 days prior to study entry*.*

4.1.11 For participants of reproductive potential, willingness to abstain from participation in a conception process (e.g. active attempt to become pregnant or in vitro fertilization). If participating in sexual activity that could lead to pregnancy, participants must use at least one reliable form of contraception listed below while receiving protocol-specified medications and for 6 weeks after stopping the medication.

1. Condoms (male or female) with or without a spermicidal agent (condoms are recommended because their appropriate use is the only contraception method effective for preventing HIV transmission).
2. Diaphragm or cervical cap with spermicide
3. IUD
4. Hormonal-based contraception

Interactions of study drugs with estrogen-based contraceptives: the effectiveness of estrogen-based contraceptives when co-administered with LPV/RTV or NVP is unknown; LPV/RTV and NVP decrease plasma levels of ethinyl estradiol; therefore, estrogen-based contraceptives are not reliable for women receiving LPV/RTV and NVP, and an alternative contraception method must be used.

NOTE: Participants who are not of reproductive potential (girls who have not reached menarche, women who have been post-menopausal for at least 24 consecutive months) girls and women who are not participating in sexual activity that could lead to pregnancy, or women who have undergone surgical sterilization, (e.g. hysterectomy, or bilateral oophorectomy, salpingotomy, or tubal ligation) are eligible without requiring the use of contraceptives. Participant report is acceptable documentation for menopause, hysterectomy, bilateral oophorectomy or tubal ligation, or sexual activity.

4.1.12 Karnofsky performance score ≥70 on at least one occasion within 45 days prior to study entry.

4.1.13 Ability and willingness of participant or legal guardian/representative to give informed consent.

4.1.14 Intent to remain in current geographical area of residence for the duration of study.

4.1.15 Willingness to attend study visits as required by the study.

## 4.2 Exclusion Criteria for Step 1

4.2.1 Receipt of any ARV (including for purposes of occupational or sexual post-exposure prophylaxis or MTCT prevention), except as noted below, at any time prior to study entry.

NOTE A: For participants in either trial, receipt of up to 10 weeks (cumulative) of ZDV alone, for MTCT prophylaxis or other purpose, which was completed at least 6 months prior to study entry, is not exclusionary.

NOTE B: For participants in Trial 1, exposure to NVP, as described in 4.1.4 is not exclusionary.

4.2.2 For participants in Trial 2, any prior exposure to NVP.

4.2.3 For participants in Trial 1, any prior exposure to NVP other than the use of SD NVP for prevention of MTCT.

4.2.4 Use of systemic cancer chemotherapy, systemic investigational agents, immunomodulators (growth factors, systemic corticosteroids, HIV vaccines, immune globulin, interleukins, interferons) or rifampin within 30 days prior to study entry.

4.2.5 Breastfeeding or pregnancy.

4.2.6 Known allergy/sensitivity to study drugs or their formulations.

4.2.7 Any condition, including active drug or alcohol use or dependence that, in the opinion of the investigator, would interfere with adherence to study requirements.

4.2.8 Serious illness requiring systemic treatment and/or hospitalization until participant either completes therapy or is clinically stable on therapy, in the opinion of the investigator, for at least 30 days prior to study entry.

4.2.9 Receipt of tuberculosis (TB) treatment within 30 days prior to study entry.

4.2.10 Use of any prohibited medications listed in section 5.4.2 within 30 days prior to study entry.

4.2.11 Current compulsory detention (involuntary incarceration) in a correctional facility, prison, or jail for legal reasons or in a medical facility for treatment of either a psychiatric or physical (e.g., infectious disease) illness.

## 4.3 Inclusion Criteria for Step 2

- - 1. Discontinuation of NVP or LPV/RTV (with or without discontinuing the NRTIs) as part of the Step 1 initial regimen due to virologic failure, toxicity, intolerance or participant/investigator discretion and willingness to initiate a treatment regimen that includes a switch to either LPV/RTV or NVP.
    2. The following laboratory values obtained within 45 days prior to Step 2 registration:
- ANC ≥750/mm^3^
- Hemoglobin ≥7.0 g/dL
- Platelet count ≥50,000/mm^3^
- AST (SGOT), ALT (SGPT), and alkaline phosphatase ≤2.5 x ULN
- Total bilirubin ≤2.5 x ULN

4.3.3 For participants who remain on TDF, an estimated creatinine clearance of ≥50 mL/min within 45 days prior to Step 2 registration using the formula:

{[140 - age(yr)]x [weight(kg)]÷[72 x serum Cr(mg/dL)]} x 0.85

## 4.4 Exclusion Criterion for Step 2

4.4.1 Contraindication to the ARV switch (NVP to LPV/RTV or LPV/RTV to NVP) as determined by the site investigator.

## 4.5 Inclusion Criteria for Step 3

**4.5.1 Completion of final Step 1 or 2 study visit between June 5, 2009 and August 27, 2009.**

**4.5.2 Willingness and ability to provide informed consent for participation in Step 3.**

## 4.6 Exclusion Criteria for Step 3

**None.**

## **4.7** Study Enrollment Procedures

**4.7.1** Prior to implementation of this protocol, sites must have the protocol and **the protocol** consent form approved by their local institutional review board (IRB). All registration materials should be forwarded to [ICTUprotocol@s-3.com](mailto:ICTUprotocol@s-3.com) at the ACTG Operations Center for a preliminary review for completeness. The ACTG Operations Center will forward the original registration packet to the DAIDS Regulatory Compliance Center (RCC) for approval. Sites must be registered with and approved by the DAIDS/RCC Protocol Registration Office. Site registration must occur before any participants can be enrolled in this study.

Once a candidate for study entry has been identified, details will be carefully discussed with the participant. The participant (or parent or legal guardian if the participant is younger than 18 years of age or under guardianship) will be asked to read and sign the consent form that was approved by both the local IRB and the DAIDS/RCC Protocol Registration Office. If the participant and legal guardian are unable to read, the process for consenting illiterate participants, as defined by the local IRB, should be followed.

**4.7.2** At entry, participants will be randomized to one arm of Step 1.  Sites will register participants to A5208/OCTANE according to standard ACTG Data Management Center procedures.

Participants who meet the requirements for Step 2 will be registered to Step 2 according to standard ACTG Data Management Center procedures.

**Participants who meet the requirements for Step 3 will be registered to Step 3 according to standard ACTG Data Management Center procedures.**

## **4.8** Coenrollment Guidelines

**Coenrollment into A5243, “Plan for Obtaining Human Biological Samples at Non-U.S. Clinical Research Sites for Currently Unspecified Genetic Analyses,” is strongly encouraged.**

**Coenrollment into other studies will be handled on a case-by-case basis. Sites should discuss coenrollment with the A5208/OCTANE CMC prior to coenrolling an A5208/OCTANE participant into another clinical study.**

# 5.0 STUDY TREATMENT

A5208/OCTANE is a study comprising two randomized clinical trials (RCT) to be conducted concurrently. Participants will be enrolled into one of the 2 RCTs as follows:

#

- Trial 1 will enroll women with prior SD nevirapine (NVP) prophylaxis for mother-to-child-transmission (MTCT) of HIV.
- Trial 2 will enroll women with no prior NVP exposure.

Study treatment is defined **for Steps 1 and 2** as any protocol-directed combination of the following drugs: NVP, LPV/RTV, FTC, TDF, EFV, ddI, and ZDV. **There is no protocol-defined study treatment in Step 3.**

## 5.1 Regimens, Substitutions, Administration, and Duration

5.1.1 Study Regimens

Step 1: Initial Regimen

At entry, participants will be enrolled in Trial 1 or Trial 2 based on their prior NVP exposure. Within each trial, participants will enter Step 1 and be randomized 1:1 to one of two treatment arms (Arm 1A or Arm 1B).

Step 2: Second Regimen

Participants in Step 1 who discontinue NVP or LPV/RTV, either because of virologic failure, toxicity, or intolerability, may enter Step 2 only if they switch to LPV/RTV or NVP, respectively.

Participants (Trial 1 and Trial 2) randomized to Arm 1A treatment will switch to Arm 2A:

LPV/RTV + 2 or more NRTIs

Participants (Trial 1 and Trial 2) randomized to Arm 1B treatment will switch to Arm 2B:

NVP + 2 or more NRTIs

Trial 1

Women with prior SD NVP exposure for MTCT of HIV prophylaxis

Trial 2

Women with no prior NVP exposure

Step 1

Arm 1A

- Nevirapine (NVP) 200mg PO QD in AM for 14 days, then 200 mg PO BID
- Emtricitabine (FTC) 200mg PO QD
- Tenofovir disproxil fumerate (TDF) 300mg PO QD

OR

- NVP (as above)
- FTC/TDF 200mg/300 mg PO QD

Arm 1B

- Lopinavir/ritonavir (LPV/RTV) 400/100mg PO BID
- Emtricitabine (FTC) 200mg PO QD
- Tenofovir disproxil fumerate (TDF) 300mg PO QD

OR

- LPV/RTV (as above)
- FTC/TDF 200mg/300 mg PO QD

Step 2

for participants who discontinue NVP

Step 2

for participants who discontinue LPV/RTV

Arm 2A

- LPV/RTV 400/100 mg PO BID
- Plus 2 or more NRTIs

Arm 2B

- Nevirapine (NVP) 200 mg PO QD for 14 days, then 200 mg PO BID
- Plus 2 or more NRTIs

The choice of NRTIs is at the discretion of the site investigator and may include any potentially viable combination of the study-provided drugs used in Step 1, and/or additional NRTIs provided by A5208/OCTANE (including ZDV and ddI), and/or non-study drugs not available through A5208/OCTANE.

Following is a table with potential NRTI choices in participants switching to Step 2 regimens due to virologic failure on Step 1. The information in this table is neither an exhaustive nor definitive list of potential NRTI choices following virologic failure; data on the relative efficacy of these regimens in this setting do not exist. Site investigators are encouraged (but not required) to discuss choice of NRTIs in Step 2 with the A5208 clinical management committee (CMC) ([actg.cmca5208@fstrf.org](mailto:actg.cmca5208@fstrf.org)).

| NRTIs in first-line regimen | NRTIs options in second-line regimen | Comments |
| --- | --- | --- |
| Truvada (TDF/FTC) | Truvada | Consider continuation of TDF/FTC as part of second regimen, particularly in participants with early virologic failure and/or participants switching from NVP to LPV/RTV and/or participants with chronic HBV infection |
| Truvada (TDF/FTC) | Truvada + ZDV | Consider addition of ZDV to TDF/FTC |
| Truvada (TDF/FTC) | ZDV/ddI | The efficacy and side effects of this combination may make it less attractive than others |
| Truvada (TDF/FTC) | ddI/ABC | If ABC is available through non-study sources |

While high-level resistance to FTC and 3TC is likely to be present in a substantial proportion of participants experiencing virologic failure on a first regimen that contains FTC, it is reasonable to consider including FTC or 3TC in a second-line regimen (as the continued presence of FTC or 3TC may promote the presence of a less fit virus and improve viral sensitivity to TDF, ZDV and d4T by selecting for virus harboring the M184V mutation, and may confer residual antiviral activity).

Participants should avoid the combination of TDF, ddI EC, and either NVP or EFV. Recent studies suggest that such combinations increase the likelihood of early virologic failure in treatment-naïve patients with high baseline viral loads.

Participants must be registered to Step 2 through the SDAC/DMC randomization system. A new prescription with the new SID number must be written for the pharmacist to dispense Step 2 medications.

Since participants who meet the protocol definition of virologic failure may have continued benefit from the Step 1 regimen, and/or may have limited second regimen options, switching to a Step 2 regimen will not be mandatory. Participants may remain on the Step 1 regimen at the discretion of the participant and site investigator despite the occurrence of virologic failure.

**Step 3**

**Participants who complete their final Step 1 or 2 study visit between June 5, 2009 and August 27, 2009, regardless of whether they are still on ART, will be registered to Step 3 at this visit and be followed for an additional 72 weeks. No study-provided ARVs will be provided at this final Step 1 or 2 visit and no study-provided ARVs may be ingested after the day of this final Step 1 or 2 visit. Any unused study-provided ARVs will be returned to the study clinic at the final Step 1 or Step 2 visit.**

**Participants on ART are expected to continue ART using locally provided ARVs. No HIV care or treatment, including ART, will be provided through A5208 in Step 3.**

**Sites are expected to make all necessary arrangements so that provision of ART can begin at a participant’s entry to Step 3. To the extent possible, participants will remain on their A5208/OCTANE regimen including the NRTI backbone. Where changes in ARVs must be made (because of local availability), participants may need to be counseled regarding possible toxicities when changing ARVs and may need to be monitored more closely for the first several weeks/months on the new ARV. Additional information is posted on the A5208/OCTANE PSWP.**

5.1.2 Study Drug Substitutions

Toxicity or intolerance

Substitution of one or two NRTIs for reasons of toxicity or intolerance (without change in LPV/RTV or NVP) will not lead to a step change.

In the case of treatment-limiting toxicity attributed to TDF (see section 7.0, Toxicity Management, for further instructions), ZDV or ddI may be substituted for TDF.

Tuberculosis

Participants who develop TB and need rifampin-containing TB treatment while on study may be offered EFV in place of NVP or LPV/RTV. All participants on TB therapy will continue to receive FTC and TDF or FTC/TDF. For participants of childbearing potential who cannot use the contraceptive methods necessary for EFV treatment (outlined below), NVP will be substituted for LPV/RTV and NVP will be continued in participants already receiving NVP. None of these temporary drug substitutions result in a step change.

EFV will be administered orally, 800 mg once daily, for the duration of rifampin-containing TB treatment and for thirty days after stopping rifampin. If 800mg once daily is not tolerated, the dose may be decreased to 600mg once daily. Thirty days after completing rifampin-containing TB treatment, participants will change back to the NNRTI or PI that they were taking prior to their development of TB.

Participants who receive rifabutin for treatment of TB may remain on NVP and LPV/RTV at unaltered doses. However, participants who receive LPV/RTV with rifabutin should have their rifabutin dose reduced to 150 mg three times weekly. Participants who receive NVP with rifabutin do not need to have dose alterations of either drug, but should be monitored carefully for rifabutin toxicity.

NOTE: Participants of reproductive potential must have a negative pregnancy test within 48 hours prior to initiating EFV. In addition, if participating in sexual activity that could lead to pregnancy while receiving EFV, participants must agree that two reliable methods of contraception, including a reliable barrier method of contraception (condoms or cervical cap) together with another reliable form of contraceptive (condoms, with or without a spermicidal agent; a diaphragm or cervical cap with spermicide; an IUD; or hormonal-based contraceptive) will be used simultaneously while receiving EFV and for 6 weeks after stopping EFV.

Participants who are not of reproductive potential, as defined in section 4.0, or whose male partner(s) has/have undergone successful vasectomy with documented azoospermia or have documented azoospermia for any other reason, are eligible without requiring the use of contraceptives. Written or oral documentation communicated by clinician or clinician’s staff confirming lack of reproductive potential by one of the following is required: physician report/letter, discharge summary, FSH measurement elevated into the menopausal range as established by the reporting laboratory. In addition, the participant’s own statement regarding her or her partner(s)’s lack of reproductive potential must be recorded in the source documentation.

If a participant reports a history of infertility based on one of the above categories or she states that her partner(s) has/have had a vasectomy but documentation of either from a clinician or clinician’s staff is not available, the participant must agree to use at least one barrier method of contraception, with a possible second method required at the discretion of the site study physician.

Pregnancy

Participants who become pregnant or begin breast-feeding and choose to continue on study may substitute ZDV for TDF, but will continue to receive FTC and either NVP or LPV/RTV, based on assignment at time of pregnancy. Women who cannot tolerate ZDV may use another NRTI such as ddI, d4T, or TDF during their pregnancies with the site investigator’s approval.

Participants who are taking EFV when they become pregnant will discontinue EFV immediately and replace it with NVP or another antiretroviral drug. It will be at the discretion of the site investigator to determine whether a woman should return to EFV following pregnancy. Breastfeeding participants will be allowed to receive EFV.

None of these temporary drug substitutions result in a step change.

5.1.3 Study Drug Administration

5.1.3.1 NVP: one 200-mg tablet will be administered orally once daily for the first 14 days (lead-in period), then orally twice daily thereafter. In Step 1, NVP must be ingested in the morning during the lead-in period. The site clinician should review the signs and symptoms of NVP-related hypersensitivity and hepatitis with the participant prior to dispensing NVP. Participants should contact the site clinician as soon as possible if they develop rash, or signs and symptoms of hypersensitivity or hepatitis. If rash occurs during the lead-in period, do not escalate the dose until the rash has resolved. After reaching full dose, if NVP dosing is interrupted for >7 days, NVP should be started with the lead-in of 200 mg once daily for 14 days, then 200 mg twice daily. NVP may be taken with or without food. **The lead-in period for NVP must never exceed 28 days. If the lead-in dosing period for NVP exceeds 28 daysd, then an alternate treatment must be sought.**

- - - 1. LPV/RTV: three soft-gel capsules (133.3 mg of LPV and 33.3 mg of RTV per capsule) will be administered orally twice daily with food.

LPV/RTV: two fixed-dose combination tablets (each tablet contains 200 mg of lopinavir and 50 mg of ritonavir) orally twice daily with or without food. Tablets should be swallowed whole and not chewed, broken, or crushed.

Lopinavir/ritonavir tablets will be available for all subjects who enroll under Version 3.0. Subjects who enrolled prior to Version 3.0 (i.e., before LPV/RTV tablets were available) may elect to switch from LPV/RTV capsules and receive the LPV/RTV tablets through A5208 at the time they consent to Version 3.0. A new prescription for the LPV/RTV tablets must be written in order for the pharmacist to dispense the new formulation.

5.1.3.3 FTC: one 200-mg capsule will be administered orally once daily with or without food.

5.1.3.4 TDF: one 300-mg tablet will be administered orally once daily with or without food.

5.1.3.5 FTC/TDF: one capsule (200 mg of FTC and 300 mg of TDF per capsule) will be administered orally once daily with or without food.

5.1.3.6 ZDV: one 300-mg tablet will be administered orally twice daily with or without food.

5.1.3.7 ddI EC: 400 mg capsules will be administered orally once daily without food for subjects who weigh >60 kg and 250 mg once daily for subjects who weigh <60 kg.

A dose of 250 mg once daily will be administered if the regimen contains TDF for adults weighing ≥60 kg, or 200 mg for adults weighing <60 kg. ddI EC capsules should be swallowed intact.

All ddI formulations should be administered on an empty stomach, at least 30 minutes before or 2 hours after a meal.

5.1.3.8 EFV will be available only for participants who develop TB and require a rifampin-containing TB regimen. The dose administered will be four 200-mg capsules orally once daily with or without food. Bedtime dosing is recommended.

5.1.4 Duration

Study drugs will be provided **in Step 1 and Step 2** for all participants. **After the day of the final Step 1 or 2 study visit, no ARVs will be provided through the study and none may be ingested after this point. Any unused study-provided ARVs will be returned to the study clinic at the final Step 1 or Step 2 visit.**

Participants who become pregnant while on study and choose to remain on study will be followed on study and will be provided study drugs through **Step 1 or 2**.

## 5.2 Study Product Formulation and Preparation

5.2.1 Nevirapine, NVP, Viramune® 200-mg tablets will be stored at 15-30°C (59-86°F).

5.2.2 Lopinavir/Ritonavir, LPV/RTV, Kaletra® 400-mg/100-mg is co-formulated to contain 133.3 mg of lopinavir and 33.3mg of ritonavir in each soft gelatin capsule. Store capsules at 2-8°C (36-46°F) prior to dispensing. Avoid exposure to excessive heat. After dispensing, if stored up to 25°C (77°F), capsules should be used within 42 days.

Lopinavir/Ritonavir, LPV/RTV tablets are co-formulated to contain 200 mg of lopinavir and 50 mg of ritonavir in each film-coated tablet. Store tablets at 20-25˚C (68-77˚F); excursions permitted from 15-30˚C (59-86˚F). Dispense in original container. For participant use: exposure of this product to high humidity outside the original container for longer than 2 weeks is not recommended.

5.2.3 Emtricitabine, FTC, Emtriva® 200-mg capsules will be stored at 15-30°C (59-86°F).

5.2.4 Tenofovir, TDF, Viread® 300-mg tablets should be stored and dispensed in the original container. Each bottle contains a silica gel desiccant canister to protect the product from humidity and should remain in the original container. TDF should be stored at 25°C (77°F), excursions permitted between 15-30°C (59-86°F).

5.2.5 Emtricitabine, FTC/Tenofovir Disoproxil Fumarate, TDF, Truvada® is co-formulated to contain 200-mg/300-mg in each capsule. Each bottle contains a silica gel desiccant canister that should remain in the original container to protect the product from humidity. Store capsules at 15-30°C (59-86°F).

5.2.6 Zidovudine, ZDV 300-mg tablets will be stored at 2-30°C (36-86°F) and protect from light.

5.2.7 Didanosine Delayed-Release Capsules Enteric-Coated Beadlet, ddI EC, Videx® EC: 125-mg, 200-mg, 250-mg, and 400-mg capsules will be stored at 25°C (77°F), excursions permitted between 15°-30°C (59°-86°F).

5.2.8 Efavirenz, EFV 200-mg capsules will be stored at 15-30°C (59-86°F).

## 5.3 Study Product Supply, Distribution, and Pharmacy

- - 1. Study Product Acquisition

These study drugs will be provided by the following companies:

- - - NVP: Boehringer Ingelheim Pharmaceuticals, Inc.
    - LPV/RTV: Abbott Laboratories, Inc.
    - FTC, TDF and FTC/TDF: Gilead Sciences, Inc.
    - ddI EC: Bristol-Myers Squibb
    - ZDV: GlaxoSmithKline
    - EFV: Merck & Co., Inc.

NVP, LPV/RTV, FTC, TDF, FTC/ TDF, ddI EC, and ZDV will be available through the NIAID Clinical Research Products Management Center. The EFV will be available or coordinated through the NIAID CRPMC. The A5208/OCTANE site pharmacist can obtain these study agents for this protocol by following the instructions in the manual, *Pharmacy Guidelines and Instructions for DAIDS Clinical Trials Networks,* in the section Study Product **Management Responsibilities**.

5.3.2 Study Product Accountability

The A5208/OCTANE site pharmacist is required to maintain complete records of all study products received from the NIAID Clinical Research Products Management Center and subsequently dispensed. Further instruction will be provided by the DAIDS Pharmaceutical Affairs Branch regarding final disposition of unused study products. The procedures to be followed are provided in the manual, *Pharmacy Guidelines and Instructions for DAIDS Clinical Trials Networks,* in the section Study Product **Management Responsibilities**.

## 5.4 Concomitant Medications **(Steps 1 and 2)**

**NOTE: Although the subsections below may be consulted for guidance in Step 3, there is no study requirement to follow these in Version 4.0 of the protocol.**

Sites must refer to the most recent study medication’s package insert or investigator’s brochure to access additional current information on prohibited and precautionary medications. To avoid drug interaction and adverse events, the manufacturer’s package inserts of the antiretroviral and concomitant agent should be referred to whenever a concomitant medication is initiated or dose changed.

Concomitant use of ingested traditional medicines is strongly discouraged while participants are taking ART.

Information on drugs without trade names, with many marketed forms, or those not available in the U.S. may be found at:

<http://www.hiv-druginteractions.org/drug/pdf/pi_col.pdf>

<http://www.ucsf.edu/hivcntr/pharmacy/Drug_Intxn_Web.htm>

5.4.1 Required Medications

No concomitant medications are required.

5.4.2 Prohibited Medications

- - - 1. Prohibited Medications with NVP

| *Medication Class* | *Prohibited Concomitant Medications* |
| --- | --- |
| Antihistamines | Astemizole (Hismanal®)  Terfenadine (Seldane®) |
| Anti-infectives | Systemic Ketoconazole |
| Alternative/Complementary | St. John’s wort (Hypericum perforatum) |
| GI Motility | Cisapride (Propulsid®) |
| Psychiatric Medication | Pimozide |
| Sedatives/Hypnotics | Midazolam (Versed®)^2^  Triazolam (Halcion®) |
| Other | Dihydroergotamine  Ergonovine  Ergotamine  Methylergonovine |

^1^ Rifampin decreases NVP serum levels. Concomitant use of Rifampin with NVP-containing regimens is not

recommended, although sometimes its use with Rifampin is difficult to avoid.

^2^ Midazolam can be used with caution as a single dose, when given in a monitored situation for procedural sedation.

- - - 1. Prohibited Medications with LPV/RTV

| *Medication Class* | *Prohibited Concomitant Medications* |
| --- | --- |
| Antiarrhythmics | Amiodarone  Quinadine |
| Antihistamines | Astemizole (Hismanal®)  Terfenadine (Seldane®) |
| Anti-infectives | Systemic Itraconazole (Sporonox®)  Rifampin, Rifapentine, Rifampicin |
| Alternative/Complementary | St. John’s wort (Hypericum perforatum) |
| HMG CoA Reductase Inhibitors | Lovastatin (Mevacor®)  Simvastatin (Zocor®) |
| GI Motility | Cisapride (Propulsid®) |
| Psychiatric Medication | Pimozide |
| Sedatives/Hypnotics | Midazolam (Versed®)^1^  Triazolam (Halcion®) |
| Other | Dihydroergotamine  Ergonovine  Ergotamine  Fluticasone (Flonase®)  Methylergonovine |

**^1^Oral midazolam is contraindicated. If lopinavir/ritonavir is co-administered with parenteral midazolam, close monitoring for respiratory depression and/or prolonged sedation should be exercised and dosage adjustment should be considered.**

5.4.3 Precautionary Medications

Use of the agents listed below may require additional monitoring of drug levels for adverse events. These PRECAUTIONARY MEDICATIONS include, but may not be limited to the following:

5.4.3.1 Precautionary Medications with NVP

| *Medication Class* | *Precautionary Concomitant Medications* |
| --- | --- |
| Anticonvulsants | Carbamazepine (Tegretol®)  Clonazepam (Klonopin®)  Ethosuximide  Phenobarbital  Phenytoin (Dilantin®) |
| Anti-infectives | Artemotil  Atovaquone (Mepron®)  Atovoquone/Proguanil (Malarone®)  Caspofungin (Cancidas®)  Clarithromycin (Biaxin®)  Dapsone  Fluconazole (Diflucan™)  Rifabutin (Mycobutin®)^1^  Systemic Itraconazole (Sporonox®)  Rifampin, Rifapentine, Rifampicin^2^ |
| Alternative/Complementary | Milk thistle (Silymarin, Silybum, marianum) |
| Antiarrhythmics | Amiodarone  Disopyramide  Lidocane |
| Calcium Channel Blockers | Diltiazem (Cardiazem® and others)  Nifedipine (Adalat® and Procardia®)  Verapamil (Calan® and Isoptin®) |
| Hormonal Agents | Glucocorticoids  Oral Contraceptives^3^ |
| Hypoglycemics | Pioglitazone (Actos®) |
| Sedatives/Hypnotics | All benzodiazepines:  Alprazolam (Xanax®)  Diazepam (Valium®)  Estazolam (ProSom®)  Flurazepam (Dalmane®)  Oxazepam (Serax®)  Temazepam (Restoril®)  Buspirone (BuSpar®)  Zaleplon (Sonata®)  Zolpidem (Ambien®) |
| Other Agents | Methadone^4^  Theophylline  Warfarin |

^1^ Data suggesting dose adjustments for rifabutin when co-administered with NVP are insufficient. Caution should be used in concomitant administration. ^2^ Rifampin decreases NVP serum levels. Concomitant use of Rifampin with NVP-containing regimens is not recommended, although sometimes its use with Rifampin is difficult to avoid.

^3^ An alternative or additional contraceptive measure should be used when estrogen-based oral contraceptives are coadministered with NVP. ^4^ The dose of methadone may need to be increased in regimens containing NVP. Participants on NVP-containing regimens should be closely monitored for symptoms of opiate withdrawals.

5.4.3.2 Precautionary Medications with LPV/RTV

| *Medication Class* | *Precautionary Concomitant Medications* |
| --- | --- |
| Analgesics | Codeine and other opiates  Propoxyphene (Darvon® and others)  Tramadol (Ultram®) |
| Antiarrhythmics | Bepridil (Bepadin, Vascor®)  Disopyramide (Norpace®)  Lidocaine (Xylocaine®  Mexiletine (Mexitil®) |
| Anticonvulsants | Carbamazepine (Tegretol ®)  Phenobarbital  Phenytoin (Dilantin®) |
| Anti-infectives | Atovaquone (Mepron®)  Atovoquone/Proguanil (Malarone®)  Caspofungin (Cancidas®)  Clindamycin (Cleocin®)  Clarithromycin (Biaxin®)  Dapsone  Systemic ketoconazole (Nizoral®)  Fluconazole (Diflucan®)  Miconazole (Monistat®)  Rifabutin (Mycobutin®)^1^ |
| Alternative/Complementary | Echinacea  Garlic Supplements  Milk thistle (Silymarin, *Silybum, marianum)* |
| Beta Blockers:  (Selected agents are listed, but caution should be taken with the entire class, especially those that are extensively hepatically metabolized. Atenolol, nadolol, and sotalol  undergo minimal hepatic metabolism.) | Alprenolol  Atenolol (Tenormin® and others)  Bisopropol (Zebeta®)  Carvedilol (Coreg®)  Esmolol (Brevibloc®)  Labetalol (Trandate®, Normodyne®)  Metoprolol (Lopressor® and Toprol XL®)  Pindolol (Visken®)  Propranolol (Inderal®)  Timolol (Blocadren®) |
| Calcium Channel Blockers:  (Dihydropyridines and others) | Amlodipine (Norvasc®)  Aranidipine  Cilnidipine  Diltiazem (Cardiazem® and others)  Felodipine (Plendil®)  Isradipine (Dynacirc®)  Lacidipine (Lacipil®)  Lercandipine  Manidipine  Nicardipine (Cardene®)  Nifedipine (Adalat® and Procardia®)  Nilvadipine  Nimodipine (Nimotop®)  Nisoldipine (Sular®)  Nitrendipine (Nitrendipine®)  Verapamil (Calan®) and Isoptin®) |
| Hormonal Agents | Estrogens and Progesterones^2^  Glucocorticoids |
| Hypoglycemics | Pioglitazone (Actos®) |
| HMG Co Reductase Inhibitors | Atorvastatin (Lipitor®)  Fluvastatin (Lescol®) |
| Psychiatric Medications: | Clozapine (Clozaril®)  Bupropion (Wellbutrin®, Zyban®)  Fluoxetine (Prozac® and others)  Paroxetine (Paxil®)  Risperidone (Risperdal®)  Venlafexine (Effexor®)  Nefazadone (Serzone®) |
| Tricyclic antidepressants | Amitriptyline (Elavil® and others)  Desipramine (Norpramin® and others)  Imipramine (Tofranil® and others)  Nortriptyline (Pamelor® and others) |
| Sedative/Hypnotics | All benzodiazepines:  Alprazolam (Xanax®)  Diazepam (Valium®)  Estazolam (ProSom®)  Flurazepam (Dalmane®)  Oxazepam (Serax®)  Temazepam (Restoril®)  Buspirone (BuSpar®)  Trazodone  Zaleplon (Sonata®)  Zolpidem (Ambien®) |
| Other Agents | Theophylline  Warfarin  Methadone^3^ |

^1^ Rifabutin should be dose-reduced to 1/4 of the recommended dose of 300 mg QD when given with LPV/RTV. A maximum dose of 150 mg QOD or three times per week is recommended. ^2^ Alternative or additional contraceptive measure should be used when estrogen-based oral contraceptives are coadministered with LPV/RTV. ^3^ The dose of methadone may need to be increased in regimens containing LPV/RTV. Participants on LPV/RTV- containing regimens should be closely monitored for symptoms of opiate withdrawals.

5.4.3.3 Precautionary Medications with FTC

The primary route of FTC elimination is by renal excretion. It undergoes glomerular filtration as well as active renal tubular secretion, most likely mediated by a cationic transporter. Theoretically, drugs that are secreted via the same carrier-mediated renal tubular transporter could compete with FTC, thus decreasing the clearance of either or both drugs. Caution should be taken when FTC is coadministered with other agents that are renally excreted.

5.4.3.4 Precautionary Medications with TDF

Coadministration of TDF with agents with either a nephrotoxic potential (for example, amphotericin B, aminoglycosides, cidofovir, acyclovir, ganciclovir) or agents that are renally excreted may increase serum drug concentrations of TDF and/or increase the concentrations of the other renally excreted agents. Additional monitoring may be indicated if participants are placed on these agents while on TDF. If ddI is coadministered with TDF, the dose of ddI should be reduced to 125mg po BID among persons weighing >60 kg. There are no guidelines for dose reduction of ddI when co-administered with TDF among persons weighing <60 kg.

Coadministration of TDF and LPV/RTV increases both the AUC and Cmax of TDF. This could result in an increase in TDF-associated toxicities (including renal disorders) and participants receiving these drugs should be monitored closely.

5.4.3.5 Precautionary Medications with FTC/TDF

See sections 5.4.3.3 and 5.4.3.4 above.

5.4.3.6 Precautionary Medications with ZDV

Caution should be taken when ZDV is co-administered with drugs known to cause pronounced anemia or other severe ZDV-associated events. ZDV dose reduction may be considered

d4T and ZDV should never be used concomitantly.

Concomitant use of ZDV with doxorubicin should be avoided since an antagonistic relationship has been demonstrated.

5.4.3.7 Precautionary Medications with ddI EC

Concomitant use of ddI EC and d4T should be avoided in general and in particular during pregnancy.

If ddI ECis co-administered with TDF, a dose reduction of ddI to 250 mg once daily for participants weighing ≥60 kg or 200 mg once daily for participants weighing <60 kg is recommended. Both drugs may be taken together with a light meal.

Co-administration of ribavirin and ddI EC is not recommended. Reports of fatal hepatic failure, as well as peripheral neuropathy, pancreatitis, and symptomatic hyperlactatemia/lactic acidosis have been reported in clinical trials.

Co-administration of ddI EC with drugs that are known to cause pancreatitis may increase the risk of this toxicity.

Antacids that contain magnesium or aluminum may increase the side effects associated with antacid components of ddI and should be used with caution.

## 5.5 Adherence Assessment

An adherence questionnaire will be administered and pill counts will be performed by site personnel at the visits indicated in the Schedule of Events (section 6.1) to determine participant adherence to all ARVs.

## 5.6 Adherence Interview (Step 3)

**In Step 3, participants will either be administered an adherence questionnaire or will be interviewed by telephone regarding adherence to their current regimen’s ARVs. No pill counts need to be performed.**

# 6.0 CLINICAL AND LABORATORY EVALUATIONS

## 6.1 Schedule of Events—Trial 1 and Trial 2**, Steps 1 and 2**

| Evaluation | Screening | Step 1 Entry  (Week 0) | Step 2 Entry | Weeks After Step 1 or Step 2 Entry^1^ | | | | | | | | After 48 weeks (Step 1 or 2) | Vir. Failure Confir-mation | **Step 1 or 2** D/C |
| --- | --- | --- | --- | --- | --- | --- | --- | --- | --- | --- | --- | --- | --- | --- |
|  |  |  |  | (± 7 d) | | | (± 14 days) | | | | |  |  |  |
|  |  |  |  | 2 | 4 | 8 | 12 | 16 | 24 | 36 | 48 |  |  |  |
| Documentation of HIV | X |  |  |  |  |  |  |  |  |  |  |  |  |  |
| Medical/Medication History | X |  |  |  |  |  |  |  |  |  |  |  |  |  |
| Concomitant Medications/Treatment Modifications |  | X | X | X | X | X | X | X | X | X | X | q 12w |  | X |
| Clinical Assessments | X | X | X | X | X | X | X | X | X | X | X | q 12w |  | X |
| Hematology & Chemistries | X | X^2^ | X |  | X |  | X |  | X | X | X | q 12w |  | X |
| Lipid Levels |  | X | X |  |  |  |  |  | X |  | X | q 48w |  | X |
| Liver Function Tests | X | X^2^ | X | X | X | X | X | X | X | X | X | q 12w |  | X |
| Pregnancy Testing | X | X |  | As indicated; perform monthly for participants taking EFV | | | | | | | | |  |  |
| HbSAg |  | X |  | Repeat as indicated | | | | | | | | |  |  |
| Lipase |  |  |  | Perform for symptoms suggestive of pancreatitis | | | | | | | | |  |  |
| CPK |  |  |  | Perform for symptoms suggestive of myositis | | | | | | | | |  |  |
| Lactate |  |  |  | Perform for symptoms suggestive of lactic acidosis | | | | | | | | |  |  |
| CD4+/CD8+ | X | X | X |  |  |  | X |  | X | X | X | q 12w |  | X |
| HIV-1 RNA, real time | X | X | X |  |  |  | X |  | X | X | X | q 12w | X | X |
| HIV-1 RNA, batched |  |  |  |  | X |  |  | X |  |  |  |  |  |  |
| Plasma for Resistance Testing |  | X | X |  |  |  |  |  |  |  |  |  | X |  |
| Stored Plasma |  | X | X |  |  | X | X |  | X |  | X | q 24w | X | X |
| Stored PBMC |  | X | X |  |  |  |  |  | X |  | X |  | X |  |
| PK Sampling, Arm 1A only |  |  |  | X | X |  |  |  |  |  |  |  |  |  |
| Adherence Assessments |  |  |  |  | X |  | X |  | X |  | X | q 24w |  |  |
| QOL/RU Assessments |  | X |  |  |  |  |  |  | X |  | X | q 24w |  |  |

^1^See section 6.2.2, “Step 2 Registration”

^2^See section 6.2.2, “Step 1 Entry”

## **6.1 Schedule of Events -** Step 3

| **Evaluation** | **Step 3**  **Entry^1^** | **Wk 12**  **(± 14 days)** | **Wk 48**  **(± 28 days) (Interview/Chart Review Only)** | **Wk 72**  **(± 28 days) or**  **Premature D/C** |
| --- | --- | --- | --- | --- |
| **ARV Treatment Modifications/Concomitant Medications** |  | **X** | **X** | **X** |
| **Clinical Assessments/Chart Review** |  | **X** | **X** | **X** |
| **Hematology & Chemistries** |  | **X** |  | **X** |
| **Lipid Levels** |  | **X** |  | **X** |
| **Liver Function Tests** |  | **X** |  | **X** |
| **Pregnancy Testing** | **X** | **As indicated** | | |
| **HbSAg** | **As indicated** | | | |
| **Lipase** | **Perform for symptoms suggestive of pancreatitis** | | | |
| **CPK** | **Perform for symptoms suggestive of myositis** | | | |
| **Lactate** | **Perform for symptoms suggestive of lactic acidosis** | | | |
| **CD4+/CD8+** |  | **X** |  | **X** |
| **HIV-1 RNA, real time** |  | **X** |  | **X** |
| **Documentation of CD4+/CD8+ & HIV-1 RNA from Local Clinic** |  | **X** |  | **X** |
| **Stored Plasma for Resistance Testing** | **X** | **X** |  | **X** |
| **Adherence Interview** |  | **X** | **X** | **X** |
| **QOL/RU Assessments** |  | **X** |  | **X** |
| **Description of Locally-Provided Care and Treatment Programs** | **X** | **X** | **X** | **X** |

**^1^ The Step 3 entry visit is expected to be conducted at the same time as the Step 1 or 2 D/C visit. Participants who are entering Step 3 will have all Step 1 or 2 D/C evaluations as well as all Step 3 Entry evaluations.**

6.2 Timing of Evaluations

- - 1. Prerandomization Evaluations **(Steps 1 and 2)**

These evaluations occur prior to the participant taking any study drugs.

Screening

Screening evaluations to determine eligibility must be completed within 45 days prior to study entry, unless otherwise specified.

- - 1. On-Study Evaluations **(Steps 1 and 2)**

Evaluations should occur after randomization and on the weeks indicated, within the following windows: ± 7 days of the visits through week 8 visit; and within 14 days of the weeks thereafter. After week 8, consecutive study visits must be separated by at least 14 days. Study drugs should be dispensed monthly to all participants whenever possible. However, a 2-month supply of study drugs may be dispensed at the site’s discretion, based on the participant’s schedule and demonstrated level of adherence to ARVs.

Step 1 Entry

Entry evaluations must occur at least 7 days after screening evaluations and no more than 3 days after randomization, and before dispensing study drugs. Hematology, chemistry, and liver function tests do not need to be repeated at entry if the screening tests were performed within 30 days prior to study entry. Participants should begin treatment as soon as possible, preferably within 72 hours after randomization. Participants who have not begun treatment within 1 week after randomization will go off study and will be replaced.

Step 2 Registration

Participants in Step 1 who discontinue NVP or LPV/RTV, either because of virologic failure, toxicity, or intolerability, may enter Step 2 only if they switch to LPV/RTV or NVP, respectively. Participants must begin a Step 2 regimen no less than 72 hours after the discontinuation of NVP or LPV/RTV and within 72 hours after Step 2 registration. Following the Step 2 Registration visit, Step 2 evaluations will follow the same schedule as Step 1.

- - 1. Evaluations for randomized participants who do not start study drugs (**Step 1)**

Participants who are randomized but withdraw from the study prior to starting study treatment should have screening, entry, and off-study forms completed and keyed. No further follow-up is required for these participants.

- - 1. Virologic Failure Confirmation Evaluations (**Steps 1 and 2)**

After receipt of results indicating virologic failure, sites should schedule an additional clinic visit (ideally within 2-**4 weeks)** for the confirmatory measurement and other evaluations listed in the Virologic Failure Confirmation column. If another visit cannot be scheduled, these evaluations must be performed at the **next attended visit.** This applies in both Steps 1 and 2, even if a participant is being followed off study treatment.

For participants whose baseline plasma HIV-1 RNA level is ≤750,000 copies/mL, virologic failure is defined as a plasma HIV-1 RNA level that is <1 log_10_ below baseline 12 weeks after treatment is initiated OR as a plasma HIV-1 RNA level that is ≥400 copies/mL at or after 24 weeks of treatment.

For participants whose baseline HIV-1 RNA level is >750,000 copies/mL, virologic failure is defined as a plasma level that is >75,000 copies/mL 12 weeks after treatment is initiated, OR as a plasma HIV-1 RNA level that is ≥400 copies/mL at or after 24 weeks of treatment.

A plasma HIV-1 RNA level that is <400 copies/mL 12 weeks after treatment is initiated is NOT considered an indication of virologic failure, regardless of the participant’s baseline plasma HIV-1 RNA level.

In all cases, the initial indication of virologic failure should be confirmed (ideally within 2 to **4 weeks and no later than the next** attended **visit**). The baseline value will be the value obtained at the study entry visit. If this value is not available, then the baseline value will be the screening value.

**Virologic Failure Confirmation Evaluations (Step 3)**

**After receipt of results indicating virologic failure in Step 3, sites are not required by the study to confirm virologic failure.**

- - 1. Discontinuation (D/C) Evaluations **(Step 1 or 2)**

Study Discontinuation These evaluations are required at the participant’s final study visit **on Step 1 or 2**.

- - 1. Premature Treatment Discontinuation (**Steps 1 and 2 only)**

Premature discontinuation of study treatment is defined in Step 1 as permanently discontinuing all study treatment prior to study completion. Premature discontinuation of study treatment is defined in Step 2 as permanently discontinuing all study treatment prior to study completion.

Participants who prematurely discontinue all study treatment will have the treatment discontinuation evaluations indicated in the schedule of events. Participants who prematurely discontinue all study treatment will be encouraged to continue in the study in an off study treatment/on-study status and receive all study evaluations per the schedule of events through to completion of the study.

- - 1. Pregnancy

**Steps 1 and 2**

Results of the pregnancy test obtained at entry must be known to be negative prior to initiation of study treatment.

A separate consent will be obtained for participants who become pregnant on study and choose to continue on study. These participants will be followed on their current schedule until 48 weeks after the final participant is randomized.

**Step 3**

**Participants who are pregnant at entry to Step 3 or who become pregnant during Step 3 may remain on study and will be provided ART according to local standard of care guidelines.**

- - 1. **Step 3 Evaluations**

**Entry**

**The Step 3 entry visit is expected to be on the same day as the Step 1 or 2 discontinuation visit. Ideally, this will be at the first q12 week visit scheduled after June 4, 2009. However, if the site has not yet registered to Version 4.0 of the protocol by the time of this visit, then the participant will remain on study and will return for the combined Step 1 or 2 discontinuation visit and Step 3 entry visit as early as possible following the site’s registration to Version 4.0 and before August 27, 2009. In these cases, sites will perform only those discontinuation visit evaluations that were not performed in the previous 6 weeks. Sites should note that, depending on timing, lipid levels may be the only discontinuation evaluation that needs to be performed for participants who are called in for a second visit between June 5^th^ and August 27^th^, 2009.**

**By the time of the Step 3 entry visit, participants should have had arrangements made to receive locally provided ART and HIV care outside of the study setting. No study-provided ARVs may be dispensed at the final Step 1 or 2 visit, nor may any study-provided ARVs be ingested after the day of this final Step 1 or 2 visit.**

**Week 12-72**

**Participants in Step 3 will be followed for up to 72 additional weeks while receiving their HIV care and treatment through local non-A5208-provided programs.**

**At week 12 (i.e., 12 weeks after entering Step 3) ±14 days, participants will return to the study clinic for the evaluations listed. At week 48 ±28 days, study staff will collect abbreviated information on vital status, changes in ART regimen, and major clinical events (e.g., new diagnoses, hospitalizations).**

**Information for the week 48 visit can be collected either by a study clinic visit, a telephone interview with the participant or a participant’s medical caregiver in the local treatment clinic(s), by a chart review (using records obtained from the local clinic(s) at which the participant is receving care), or from other reliable sources. At week 72 ±28 days, participants are expected to return for a final study clinic visit for the evaluations listed. In addition, although participants are expected to come to the study clinic for the weeks 12 and 72 visits, much of the information requested at these visits can be obtained from these sources if the participant misses the visit.**

**Premature Discontinuation**

**Participants who elect not to complete all Step 3 visits will be asked to come in for a premature discontinuation visit at which the evaluations listed in this column in the Step 3 SoE will be performed. If a visit cannot be conducted, then a chart review will suffice.**

## 6.3 Special Instructions and Definitions of Evaluations

All clinical and laboratory information required by this protocol is to be present in the source documents. Sites must refer to the Source Document Guidelines on the ACTG Web site for information about what must be included in the source document: [**http://www3.niaid.nih.gov/research/resources/DAIDSClinRsrch/PDF/SourceDocAppndx.pdf**](http://www3.niaid.nih.gov/research/resources/DAIDSClinRsrch/PDF/SourceDocAppndx.pdf)

All evaluations are to be recorded on the CRF and keyed into the database unless otherwise specified.

6.3.1 Documentation of HIV **(Steps 1 and 2)**

Rapid HIV test or any licensed ELISA test kit, confirmed at the study-associated, DAIDS-approved laboratory with either an ELISA or IFA or Western Blot or plasma HIV-1 RNA. Discordant confirmatory results should be followed by plasma HIV-1 RNA determination performed at a DAIDS-approved laboratory for quality control purposes.

6.3.2 Medical/Medication History **(Steps 1 and 2)**

Medical History

A medical history, including allergies to any medications and their formulations, must be collected at screening. All diagnoses identified by the ACTG Criteria for Clinical Events and Other Diseases ([http://www.fstrf.org/ACTG/appendices/appendices.html](http://www.fstrf.org/ACTG/appendices/appendicies.html)) must be recorded on the CRF.

Medication History

A medication history must be collected at screening, including a complete HIV treatment history, with start and stop dates of any antiretroviral medication (estimated if the exact dates cannot be obtained), immune-based therapy, or HIV-related vaccines, and blinded study medications.

A complete treatment history of any prescription medications taken for the treatment or prophylaxis of opportunistic infections, including actual or estimated start and stop dates, must be recorded. Current prescription medications being taken for the treatment or prophylaxis of opportunistic infections must be recorded on the CRF. Any history of treatment of TB disease or latent TB infection, including specific medications and actual or estimated start and stop dates, must be recorded on the CRF.

All ARVs taken at any time prior to entry must be recorded on the CRF. For participants in Trial 1, the number of times SD NVP prophylaxis was taken and the estimated date(s) of ingestion must be recorded, along with the nature of the evidence supporting actual ingestion of each SD NVP course. Participants should be shown an actual NVP tablet or a picture of a NVP tablet, for visual recognition.

All other current medications must be recorded. With the exception of the agents listed below, current medications must be recorded on the CRF. Sites should contact the study team if there are questions regarding the reporting requirements of drugs not listed below.

- acetaminophen/paracetamol
- antacids
- calamine lotion
- castor oil
- ferrous sulfate or other iron preparations
- hydrocortisone topical cream
- ibuprofen
- multivitamins and prenatal vitamins other than Vitamin B6 or B complex
- chlorpheniramine
- diphenydramine
- folate.

All other prescription and nonprescription medications (in addition to those noted above) taken within 45 days prior to entry, including actual or estimated start and stop dates, must also be recorded.

Current alternative therapies and traditional treatments are to be recorded as yes/no on the CRF. If answered yes, the alternative therapy or traditional treatment must be listed or described in the source documents.

6.3.3 Concomitant Medications/Treatment Modifications

Concomitant Medications

Beginning at entry **and during Steps 1 and 2**, all concomitant medications initiated or discontinued since the last visit will be recorded on the CRF, except as noted in 6.3.2 above. Alternative/traditional treatments are to be recorded as yes/no on the CRF. If answered yes, the alternative therapy or traditional treatment must be listed or described in the source documents. Nonprescription medications will be recorded in the source documents only.

**On Step 3, the only concomitant medications that need to be recorded on the CRF are as follows:**

- - - **medications for TB treatment**
    - **medications for TB prophylaxis**
    - **cotrimoxazole prophylaxis**
    - **systemic antifungal treatment or prophylaxis (do not include topical treatments)**
    - **oral, injectable, or implantable contraceptives**
    - **systemic anticancer chemotherapy**

**In Step 3, sites should also record other concomitant medications that they become aware of on the source documents only.**

Treatment Modifications

All modifications to study drugs including initial doses, participant-initiated and/or protocol-mandated interruptions of >3 consecutive days, and permanent discontinuation of study drugs **(in Steps 1 or 2) or of any ARVs (in Step 3)** will be recorded on the CRF at each visit. Participant-initiated and protocol-mandated interruptions include both inadvertent and deliberate interruptions of any study drugs **(in Steps 1 or 2) or of any ARVs (in Step 3)**.

Participants initiating treatment with study-supplied ARVs which are not part of the originally randomized treatment regimens should be evaluated by the site investigator to determine whether there are any contraindications.

**At entry to Step 3, participants will discontinue all study-provided ARVs and initiate locally provided ARVs. Initiation of locally provided ARVs will be recorded on the CRF. At all subsequent Step 3 visits, all modifications to locally-provided ARVs will be recorded on the CRF (as noted above).**

6.3.4 Clinical Assessments

Complete Physical Exam **(Steps 1 or 2)**

A complete physical exam will be performed at screening and at discontinuation only and will include axillary or oral temperature (centigrade), pulse, height (cm), weight (kg), blood pressure, and examination of the following body compartments: HEENT (head, eyes, ears, nose, and throat), neck, chest, heart, abdomen, extremities, skin, and neurologic. All information should be recorded on the CRF.

Targeted Physical Exam **(Steps 1 or 2 and beginning at week 12 in Step 3)**

A targeted physical examination will be performed at each study visit, beginning at entry. Each examination will include documentation of vital signs (axillary or oral temperature [centigrade], blood pressure, weight [kg], and respiratory rate) and will be driven by any signs or symptoms previously identified that the participant has experienced since the last visit. All information should be recorded on the CRF.

Signs and Symptoms

At entry, all signs/symptoms, regardless of grade, that occurred within 30 days prior to entry must be recorded on the CRF.

After entry**, on Steps 1 and 2,** all signs, symptoms, HIV-related and AIDS-defining events, deaths and toxicities must be documented. Any signs or symptoms that led to a change in treatment, regardless of grade, must be recorded on the CRF. All WHO Stage 3 or 4 signs or symptoms must be reported on the CRFs. All rashes, regardless of grade, must be recorded on the CRF. All other signs and symptoms Grade ≥3 must be recorded on the CRF.

**In Step 3, only signs or symptoms that led to a change in treatment or are associated with a reportable diagnosis, regardless of grade, must be recorded on the CRF.**

Sites should refer to The Division of AIDS Table for Grading the Severity of Adult and Pediatric Adverse Events, Version 1.0, December 2004, which is available on the RCC website: <http://rcc.tech-res-intl.com/>.

Diagnoses**:** **Step 1, Step 2, and Step 3**

After entry, only diagnoses identified by the ACTG Criteria for Clinical Events and Other Diseases ([http://www.fstrf.org/ACTG/appendices/appendices.html](http://www.fstrf.org/ACTG/appendices/appendicies.html)) **as well as any other diagnoses (using a general code) that the site considers significant for any reason (due to severity of the illness, impact upon participant’s ART, WHO Stage III-IV or AIDS-defining diagnosis, or for any other reason),** since the last visit must be recorded on the CRF, along with any laboratory values and/or signs and symptoms associated with each diagnosis. Sites must refer to the study CRF for the appropriate appendix used for the current ACTG criteria. Additional diagnoses that are not specifically included in the appendix may be reported at the site investigator’s discretion. **Non-specific, mild disorders (such as mild skin, eye, ear, nose, throat, or GI disorders without a specific code) do not need to be reported.**

Pregnancy**: Step 1, Step 2, and Step 3**

If a woman becomes pregnant and remains on study, pregnancy-related information will be reported on a CRF when the site becomes aware of the pregnancy. Pregnancy-related information will include obstetrical history, gestational age estimation (based upon LMP, physical examination, and/or ultrasound, if available), pregnancy outcome (spontaneous abortion, induced abortion, delivery of live birth or stillbirth and/or intrauterine fetal demise), date of delivery/outcome, mode of delivery, and basic information regarding fetus/newborn (singleton/twins/triplets etc, gender, Apgar score/weight if available, physical examination and gestational age by examination, if available), antepartum and postpartum medical complications, and additional intrapartum antiretroviral prophylaxis, if any.

Pregnancies that occur on **Step 1 or Step 2** should be reported to The Antiretroviral Pregnancy Registry by fax at +44 1895 825 005. More information is available at [www.apregistry.com](http://www.apregistry.com/).

**Step 3 Information Related to Local Care**

**At weeks 12, 48 and 72, information will be collected from each participant (and reported on CRF) regarding the clinic(s) at which the participant is receiving HIV care and treatment; the frequency of visits attended at these clinics (expected and actual); the frequency of ARV collection (expected and actual); and obstacles that the participant is experiencing in accessing care, including obtaining and taking uninterrupted ART.**

**Step 3 Clinic Visit/Chart Review**

**At Step 3 week 12 and week 72, a study clinic visit is expected.**

**At Step 3 week 48, a telephone interview or home visit or other non-study clinic visit or discussion with the participant or her care providers is expected. The information listed for this visit in the Step 3 Schedule of Events table is expected to be obtained either directly from the participant or if the participant is unable to be contacted but has not formally withdrawn from the study, from any available documentation from local providers of clinical care.**

**Note that all reasonable efforts should be made to have participants return for the Step 3 week 72 visit.**

**For the week 12 and week 72/Premature Discontinuation visits, some information listed for these visits in the Step 3 Schedule of Events table may also be obtained either directly from the participant or, if the participant is unable to be contacted but has not formally withdrawn from the study, from any available documentation from or discussion with local providers of clinical care.**

LABORATORY EVALUATIONS

At entry, laboratory values obtained **at the entry visit and** within 45 days prior to entry will be recorded on the CRF, regardless of grade. Only the most recent value should be recorded for any screening laboratory evaluation that is repeated at entry. Guidelines for reporting immunologic and virologic evaluations appear in sections 6.3.13 and 6.3.14, below.

After entry, all **triglyceride,** cholesterol, LDL **(calculated or measured), and** HDL **(fasting or non-fasting)** values and all Grade ≥2 elevations in ALT, AST, or creatinine must be recorded on the CRFs. All other Grade ≥3 laboratory toxicities must be recorded on the CRFs. Any laboratory results that led to a change in treatment, regardless of grade, must be recorded on the CRFs. **All laboratory values that define a WHO Stage 3 or 4 illness must be reported on the CRFs.** When obtained, all lipase, CPK, and lactate levels will be recorded in the CRF, regardless of grade.

Sites should refer to the Division of AIDS Table for Grading the Severity of Adult and Pediatric Adverse Events, Version 1, December 2004, which can be found on the RCC Web site: <http://rcc.tech-res-intl.com/>.

6.3.5 Hematology & Chemistries

Hematology

Hemoglobin, hematocrit, red blood cells (RBC), mean corpuscular volume (MCV), white blood cell count (WBC), differential WBC, absolute neutrophil count (ANC), and platelets.

If applicable, samples for hematology evaluations should be collected within 4 weeks after the initiation of ZDV.

Blood Chemistries

Sodium, potassium, chloride, glucose, bicarbonate, creatinine, and albumin.

6.3.6 Lipid Levels

Triglycerides, cholesterol, LDL (calculated or measured), and HDL (fasting or non-fasting). Triglycerides will also be evaluated when samples are collected for suspected pancreatitis (section 6.3.10).

6.3.7 Liver Function Tests

Total bilirubin, AST (SGOT), ALT (SGPT), and alkaline phosphatase.

6.3.8 Pregnancy Testing

For women with reproductive potential: serum or urine β-HCG (urine test must have a sensitivity of 25-50 mIU/mL). After screening and entry, complete pregnancy testing only when pregnancy is suspected except as noted below.

NOTE: Participants of reproductive potential who are participating in sexual activity that could lead to pregnancy must have a pregnancy test before initiating EFV and monthly while receiving EFV and again 1 month after discontinuing EFV.

6.3.9 Hepatitis B Serologies (HbSAg)

Hepatitis B surface antigen must be performed at entry and recorded on the CRF. After entry, complete HbSAg only when hepatitis B is suspected.

6.3.10 Lipase

Lipase will be measured only when needed for evaluation of suspected pancreatitis. A triglyceride level should be drawn with the lipase (fasting or non-fasting). If a baseline measurement is needed, it will be obtained from stored samples.

If applicable, samples for lipase evaluation should be collected within 4 weeks after the initiation of ddI.

6.3.11 Creatine Kinase (CK)

CK will be measured only when needed for evaluation of suspected myositis. If myositis is indicated on the initial measurement, a repeat sample may be drawn for confirmation.

6.3.12 Lactate

Lactate levels should be collected only when needed for evaluation of suspected lactic acidosis. The participant should sit, relaxed for 5 minutes prior to venipuncture.  The participant should be instructed not to clench her fist before or during the procedure and to relax the hand as much as possible.  If possible, a tourniquet should not be used.  If a tourniquet is necessary, it should be applied lightly and the lactate should be drawn first, before the other samples with the tourniquet still in place.  The specimen should be placed on ice immediately and sent to the laboratory for immediate processing, preferably within 30 minutes of collection.  The Lactic Acidosis Guidelines on the ACTG website [**https://actgnetwork.org/protocols/psmet.aspx**](https://actgnetwork.org/protocols/psmet.aspx)**.**

IMMUNOLOGIC STUDIES

**NOTE: in Step 3, Section 6.3.13 applies only to immunologic studies performed as part of the study, and not necessarily to CD4+/CD8+ performed as part of a local treatment program.**

6.3.13 CD4+/CD8+

Determinations of CD4+ and CD8+ cell counts and subset percentage evaluations should be performed at the same DAIDS-approved laboratory, if possible, throughout the course of the study.

Eligibility will be based on the screening value.

Because of the diurnal variation in CD4+ and CD8+ cell counts, determinations for individual participants should be obtained consistently in either the morning or the afternoon throughout the study, if possible.

All CD4+ and CD8+ cell count results will be recorded on the CRFs.

VIROLOGIC STUDIES

**NOTE: in Step 3, Section 6.3.14 applies only to virologic studies performed as part of the study, and not necessarily to HIV-1 RNA performed as part of a local treatment program.**

The A5208/OCTANE Manual of Operations contains collection, processing, storage, and shipping instructions.

6.3.14 Plasma HIV-1 RNA

Determinations of HIV-1 RNA levels must be performed within 45 days prior to entry at a DAIDS-approved laboratory. All evaluations are to be done using the Roche Amplicor HIV-1 Monitor Assay (version 1.5, standard not ultra sensitive).

Plasma HIV-1 RNA will be run in real time as indicated on the Schedule of Events (section 6.1). For participants continuing treatment beyond 48 weeks, real-time HIV RNA will be repeated every 12 weeks.

In addition, plasma will be stored for batched, retrospective HIV-1 RNA assays from visits indicated in section 6.1.

All plasma HIV-1 RNA results will be recorded on the CRFs.

6.3.15 Plasma for Resistance Testing

Plasma for resistance testing (routine genotyping and minority variants assays) will be collected at Step 1 and Step 2 entry and confirmation of failure visits. All samples will be assayed with the ViroSeq HIV-1 Genotyping System (FDA-cleared population-based genotyping assay). Selected samples will be analyzed with one or more research assays for HIV minority variants, some of which are mentioned in section 2.1 (Background). **Plasma will also be stored for possible resistance testing at Step 3 registration, and at 12 and 72 weeks on Step 3.**

Resistance testing will be performed retrospectively at selected centralized, experienced laboratories (to be identified at a later date). Most potential study sites will not have the ability to perform real-time resistance testing. In sites that have this capability, resistance testing will be allowed for purposes of clinical care. Such testing would be performed independently of trial-related testing, and not at the expense of A5208/OCTANE. Even if such testing is obtained outside of the trial, samples for resistance testing will still be stored as outlined in section 6.1, and resistance testing will still be performed at the centralized laboratories using the ViroSeq system as described above. This will provide a uniform set of resistance data at all study sites. Results of resistance testing performed using trial samples at the centralized laboratories will not be used for the management of individual participants.

Sites will be notified by the DMC of which samples to ship and when to ship stored samples to the centralized laboratories for resistance testing. A schedule (e.g. quarterly) will be prepared for batch shipment of samples from each site. During the study, an attempt will be made to transfer relevant technologies and skills (such as real-time genotyping) to local laboratories.

HIV Subtyping

HIV subtyping will be performed on one sample from each participant. Subtyping will be performed by phylogenetic analysis (computer-based sequence comparisons) of *pol* region HIV sequences obtained from genotyping with the ViroSeq system. In some cases, additional regions of the HIV genome (e.g. *env*, *gag*) will be sequenced for further characterization of HIV subtypes.

**6.3.16 Documentation of CD4+/CD8+ and Plasma HIV-1 RNA from Local Clinics (Step 3)**

**Results of all CD4+/CD8+ and HIV-1 RNA testing that was performed at the local treatment clinic will be recorded on the CRF.**

6.3.17 Stored Plasma **(Steps 1 and 2)**

Additional samples for resistance testing and other virologic assays will be collected at the visits indicated in section 6.1.

6.3.18 Stored PBMC **(Steps 1 and 2)**

Cell pellets prepared from whole blood collected at visits indicated in section 6.1 will be stored to enable future analysis of proviral HIV DNA. Sites will be notified by the DMC of which samples to ship and when to ship stored samples to centralized laboratories for testing.

PHARMACOLOGIC STUDIES **(Steps 1 and 2 only)**

The A5208/OCTANE Manual of Operations contains collection, processing, storage, and shipping instructions.

6.3.19 Pharmacokinetic (PK) Sampling, Arm 1A Only

For participants in Arm 1A (Step 1, Arm A only), a PK sample will be collected 14 (± 7) days following randomization and prior to NVP dose escalation to BID administration. Although the PK sampling may occur at a time when a participant has been taking NVP for less than 2 weeks, dose escalation should occur only after a participant has taken NVP for at least 14 days.

A second PK sample will be collected 28 (± 7) days following randomization.

At each of these visits, participants will be asked to report the time of their last dose of NVP (i.e., their morning dose on the day of the PK sampling) and to have maintained a medication diary for the previous 72 hours. Participants will be asked to report all their NVP doses for the previous three days. Samples should not be collected from participants who have missed any dose of NVP during the previous 72 hours prior to the PK sampling.

In addition, Arm 1A participants who experience a NVP-related toxicity (i.e., rash, regardless of grade, or Grade ≥2 transaminase change) will be asked to return to the clinic for the collection of an additional PK sample within 3 days of the toxicity’s latest occurrence. Only the time of the last dose of NVP should be recorded on the CRF. A PK sample should not be collected from participants who have missed all NVP doses for the previous 3 days.

OTHER ASSESSMENTS

6.3.20 Adherence Assessments

**In Steps 1 and 2, t**he adherence interviews and pill counts for each antiretroviral drug the participant is taking will be performed by site personnel according to section 6.1, Schedule of Events, and recorded on the CRFs. These assessments will require an additional 15 minutes.

NOTE: Site personnel are defined as site pharmacist, nursing staff, clinician, or other trained clinical personnel (e.g., adherence counselors, social workers).

**In Step 3, participants will either be administered an adherence questionnaire or will be interviewed by telephone regarding adherence to their current regimen’s ARVs. No pill counts need to be performed.**

6.3.21 Quality of Life (QOL)/Resource Utilization (RU) Assessments

Beginning at entry, a short questionnaire will be used to assess QOL/RU every 24 weeks on study **and as indicated in Step 3**. This questionnaire will require an additional 5-10 minutes to complete.

- - 1. **Description of Local Treatment and Care Programs (Step 3)**

**Specific information regarding the nature of monitoring (e.g., clinical, virologic, immunologic, adherence) and frequency of visits in the local treatment program will be captured from the participant and from her medical records/care providers, if available. This information will be captured for each treatment clinic at which Step 3 study participants are receiving care. In addition, any information regarding unexpected interruptions in drug supply or other issues related to drug supply or care provision should also be noted at each time point indicated in section 6.1, Schedule of Events for Step 3.**

# TOXICITY MANAGEMENT

**Steps 1 and 2**

Unanticipated and anticipated toxicities will be graded according to the Division of AIDS Table for Grading the Severity of Adult and Pediatric Adverse Events, Version 1.0, December 2004 (see A5208/OCTANE Manual of Operations, Version 2.0).

Toxicity management at non-U.S. sites may require greater reliance on clinical symptoms and clinician judgment if access to laboratory, radiologic, and other testing is limited locally.

This section provides guidelines for management of toxicities related to NVP, LPV/RTV, FTC, TDF, FTC/TDF, and, as required, ZDV, ddI, and EFV only. When one study drug is held for resolution of toxicity, all study drugs in the regimen should be held concurrently. Participants taking FTC/TDF who are advised, based on toxicity, to discontinue either the FTC or TDF component, may continue to receive the other component as an individual drug.

Toxicities related to non-study drugs should be handled according to the package insert and by best medical judgment.

Every attempt should be made to continue to follow participants who discontinue study treatment because of a Grade 3 or 4 adverse event until resolution of the adverse event can be documented.

**Step 3**

**In Step 3, all toxicity management will be according to local standard of care. Although the sections below may be consulted for guidance, there is no study requirement to follow these in Version 4.0 of the protocol.**

## 7.1 Dosage Modification Instructions

7.1.1 If NVP is interrupted for more than 7 days, the medication must be reintroduced with a 14-day lead-in period (i.e. NVP 200 mg po QD for 14 days, then 200 mg po BID thereafter).

7.1.2 There will be no dose reductions for NVP, LPV/RTV, FTC, TDF, or FTC/TDF. As indicated in subsequent sections, dose reductions of ZDV, ddI, or EFV may be necessary. Dose reductions are specified in the table below.

Dose Reduction Instructions

| Drug | Initial Dose | Reduced Dose |
| --- | --- | --- |
| ZDV | 300 mg BID | 200 mg BID |
| ddI^1^ | 200 mg BID | 125 mg BID |
| ddI^2^ | 125 mg BID | 125 mg QD |
| EFV^3^ | 800 mg QD | 600 mg QD |

^1^For participants weighing >60 kg.

^2^For participants weighing <60 kg.

^3^ For participants taking rifampin only.

Following dose reduction of study medication(s), a return to full dose(s) should be considered if signs, symptoms, and/or laboratory abnormalities return to pre-entry/entry levels.

## 7.2 Guidelines for Most Grade 1 or 2 Toxicities

Participants who develop a Grade 1 or 2 adverse event or toxicity may continue study treatment without alteration of the medication or dosage, with the exceptions noted below in section 7.5.

## 7.3 Guidelines for Most Grade 3 Toxicities

Participants who develop a Grade 3 adverse event or toxicity judged to be study drug-related should have one or more of their study ARVs switched or the entire regimen held. The participant should be re-evaluated weekly until the adverse event returns to Grade ≤2, at which time study treatment may be reintroduced at the discretion of the investigator or according to standard practice, depending upon the type of adverse event. Investigators are encouraged to discuss toxicity management with the A5208/OCTANE CMC, which should be notified of regimen changes or modifications and, as necessary, consulted for advice. If the relationship between study drug and the Grade 3 event is uncertain, the study drug of concern may be resumed at the discretion of the site investigator, only after discussion with the A5208/OCTANE CMC, and with the exceptions noted below in section 7.5.

Participants experiencing adverse events requiring permanent discontinuation of NVP or LPV/RTV study treatment should be followed weekly until resolution of the adverse event to Grade ≤1 or until stabilized and no longer in need of such frequent monitoring, as determined by the site investigator.

## 7.4 Guidelines for Most Grade 4 Toxicities

Participants who develop a symptomatic Grade 4 adverse event or toxicity will have all study treatment withheld until resolution of the adverse event to a Grade ≤2. Alternative study-provided or non-study provided medications should replace the study treatment thought to be most likely related to the adverse event, once ART is restarted. If the relationship between study drug and the symptomatic Grade 4 event is uncertain, the study drug of concern may be resumed at the discretion of the site investigator, only after discussion with the A5208/OCTANE CMC, and with the exceptions noted below in section 7.5.

Participants with Grade 4 asymptomatic laboratory abnormalities may continue study treatment after discussion with the A5208/OCTANE CMC only if the site investigator has compelling evidence that the toxicity is NOT related to the study treatment and with the exceptions noted below in section 7.5.

Participants experiencing adverse events requiring permanent discontinuation of NVP or LPV/RTV study treatment should be followed weekly until resolution of the adverse event to Grade ≤1 or until stabilized and no longer in need of such frequent monitoring, as determined by the site investigator.

## 7.5 Management of Laboratory Abnormalities and Clinical Syndromes

7.5.1 Rash

Rash Management for Participants Not on NVP or EFV

Grade 1 – 2:

Study treatment should continue without interruption. Participants with a Grade 1 or 2 rash may be treated symptomatically with permitted antipyretic, antihistamine, and/or nonsteroidal anti-inflammatory medications, but should be monitored closely by the site investigator.

Grade 3:

All study treatment should be held for any Grade 3 rash, unless the rash is determined to be unrelated to study medications. May restart study medications if clinically indicated when resolution to Grade ≤2.

Grade 4:

Discontinue all study medications permanently.

Rash Management for Participants on NVP or EFV

Rash of Any Grade:

For rash of any grade in participants on NVP or EFV, ALT/AST, CBC with differential, and creatinine should be performed promptly and participants evaluated for signs and symptoms relating to clinical hepatitis and hypersensitivity reactions, including:

(a) systemic symptoms (fever, clinical hepatitis, muscle or joint aches);

(b) allergic symptoms (urticaria, wheezing);

(c) exfoliation;

(d) mucosal involvement or conjunctivitis;

(e) elevated ALT or AST above entry value;

(f) eosinophilia; or

(g) elevated serum creatinine Grade> 2

The presence of any of the above (in the absence of another clear explanation) should lead to immediate and permanent discontinuation of NVP or EFV, regardless of the grade of the rash.

In addition, if participants have signs or symptoms of clinical hepatitis (which may include nausea and/or vomiting, anorexia, jaundice, acholic stools, hepatomegaly, hepatic tenderness, fever, fatigue, arthralgia) and rash of any grade, NVP or EFV should be permanently discontinued.

Grade 1 – 2:

For participants on NVP or EFV who develop a Grade 1 or 2 rash but with none of the clinical or laboratory abnormalities listed above as being consistent with HSR, and no evidence of clinical hepatitis, NVP or EFV may be continued with very close follow-up, at the discretion of the site investigator. The presence or development of any signs/symptoms or laboratory abnormalities associated with HSR should lead to the permanent discontinuation of NVP or EFV.

Grade 3 – 4:

For participants on NVP or EFV who develop a Grade 3 or 4 rash, but with no constitutional symptoms, no increase above baseline of the LFTs, and no evidence of clinical hepatitis, all ART should be discontinued. If rash resolves to ≤ Grade 1, ART that does not contain either NVP or EFV may be restarted, in consultation with the A5208/OCTANE CMC.

Urticaria – Mild to Moderate:

For participants who have mild to moderate urticaria, without constitutional symptoms, without increases above baseline of the LFTs, or without evidence of clinical hepatitis, NVP or EFV may be continued with close follow-up at the discretion of the site investigator. If participants have an urticarial rash and NVP or EFV is discontinued for any reason, neither NVP nor EFV should be restarted.

Other Considerations:

Pruritus or rash with pruritus and minor accompanying symptoms may be managed with antihistamines, antipyretics, and/or nonsteroidal anti-inflammatory medications.

The use of prednisone to prevent NVP-associated rash is not recommended although it may be used to treat severe HSR or rash.

Participants who experience rash during the first 2 weeks of the NVP lead-in period of 200 mg/day should not have their NVP dose increased until the rash has resolved.

Participants experiencing HSR to NVP or EFV should discontinue all study treatment and must not be rechallenged with the causal agent. NVP or EFV should be permanently discontinued. If the participant was receiving NVP or EFV, LPV/RTV should replace NVP or EFV.

Participant should be advised to contact the site investigator immediately if there is any worsening of the rash, or if systemic signs or symptoms develop that could be compatible with a HSR or clinical hepatitis.

If the rash is considered most likely due to concomitant illness or medication, standard management, including discontinuation of the likely causative agent, should be undertaken. If no other causative factor is found after clinical evaluation, participants should be treated symptomatically until the rash resolves.

Upon resolution to Grade ≤1, the site investigator should discuss with the A5208/OCTANE CMC whether re-initiation of ART is appropriate and which agents should be part of the treatment regimen.

If the rash that resulted in temporary discontinuation of study treatment is thought to be secondary to EFV or NVP then, once symptoms have resolved to Grade ≤1, the NRTIs may be reintroduced along with an alternative agent third antiretroviral drug (LPV/RTV, if the participant has not yet taken this study drug).

All Grade >2 skin rashes and rashes requiring treatment discontinuation (permanent and temporary) should be reported to the A5208/OCTANE CMC.

For persistent rash, if the rash does not resolve within 14 days of onset, the A5208/OCTANE CMC should be contacted.

In the event that a Grade 2, 3, or 4 rash fails to resolve, increases in severity, is associated with systemic (fever, malaise, nausea) or allergic (e.g., urticaria) symptoms, or is associated with mucosal or target lesions, all study treatment should be discontinued.

7.5.2 AST and ALT Elevations

Nearly all antiretrovirals can cause alterations in liver functions tests. Further, concomitant illness may also alter these laboratory parameters. Therefore, changes in AST or ALT should be evaluated within the clinical context of the abnormalities.

The participant must be carefully assessed for any symptoms or signs of hepatotoxicity, including fatigue, malaise, anorexia and nausea, jaundice, acholic stools, RUQ pain or hepatomegaly. If symptoms or signs of clinical hepatitis are present, study treatment must be discontinued (see below).

At study entry, hepatitis B surface antigen (HBsAg), will be obtained. Results will be made available to site investigators to facilitate management of hepatitis B coinfected participants in the event that FTC or TDF need to be discontinued, which could potentially worsen hepatitis B disease.

Grade 1

- For study participants with Grade 0 ALT and AST at study entry, an increase to Grade 1 ALT or AST even in an asymptomatic participant may be of concern (particularly among those on NVP).
- ALT and AST must be repeated as soon as possible (at most within 1 week) of a new Grade 1 ALT or AST. Study treatment may be continued while repeating ALT and AST as long as the participant is asymptomatic.
- Participants with a confirmed Grade 1 ALT or AST who are asymptomatic may continue study medications with continued close observation.

Asymptomatic Grade 2

- Participants should have ALT/AST re-checked as soon as possible (at most within 1 week) and then be followed weekly until levels are Grade ≤1. The frequency of follow up may be altered at the discretion of the site investigator following consultation with the A5208/OCTANE CMC.
- Among participants taking NVP or EFV, if the repeat ALT/AST remains Grade 2, NVP or EFV must be permanently discontinued and substituted with LPV/RTV, unless the site investigators firmly attribute the elevation in ALT/AST to concomitant medication or illness.
- Careful assessments should be undertaken for alcohol use, non-study medication-related drug toxicity, the lactic acidosis syndrome, and viral hepatitis as the cause of the transaminase elevation. If the AST/ALT elevation is considered most likely to be due to concomitant illness or medication, standard management, including discontinuation of the likely causative agent, should be undertaken.

Asymptomatic Grade 3

- Participants should have ALT/AST re-checked as soon as possible (at most within 1 week) and then be followed weekly until levels are Grade ≤1.
- Participants not taking NVP or EFV: if an asymptomatic Grade 3 ALT/AST is attributed to concomitant illness or medication, study medications may be continued at the discretion of the site investigator, and treatment of the underlying illness and/or removal of the likely causative agent should be undertaken.
- Participants taking NVP or EFV: study treatment should be discontinued and ALT/AST followed weekly until levels are Grade ≤1, after which study treatment without NVP or EFV may be resumed (NVP and EFV should be permanently discontinued).

Asymptomatic Grade 4

- All study medications should be stopped, and ALT/AST followed at least weekly until Grade ≤1, at which time treatment may be resumed in consultation with the CMC. If the participant was taking NVP or EFV before the Grade 4 ALT/AST occurred, either of these medications should be considered the most likely cause of the elevations and should not be given to the participant again. Other study medications may be restarted if the laboratory abnormalities were thought to be secondary to a concomitant illness or non-study medication.
- If Grade 4 elevations recur in the absence of an NNRTI drug, all current ART should be discontinued. Subsequent ART regimens may be considered, at the discretion of the site investigator in consultation with the A5208/OCTANE CMC.
- Careful assessments should be undertaken for alcohol use, non-study medication-related drug toxicity, the lactic acidosis syndrome, and viral hepatitis as the cause of the transaminase elevation. If the AST/ALT elevation is considered most likely to be due to concomitant illness or medication, standard management, including discontinuation of the likely causative agent, should be undertaken.

Symptomatic Hepatitis

- Participants taking NVP or EFV should be monitored carefully for the development of signs and symptoms of hepatitis, which include fatigue, malaise, anorexia, nausea, acholic stools, bilirubinuria, jaundice, liver tenderness, or hepatomegaly, with or without initially abnormal serum transaminase levels. Anyone with these signs and symptoms must seek medical attention promptly and have LFTs performed immediately.
- Symptomatic participants must discontinue NVP immediately if AST or ALT increases by 1 or more grades above baseline.
- If the site investigator determines that the participant has clinical hepatitis with or without LFT abnormality and NVP cannot be excluded as the cause, NVP should be permanently discontinued and not restarted after recovery.

7.5.3 Creatinine Clearance for Participants Receiving TDF or FTC/TDF

Estimated creatinine clearance <50 mL/min

- Estimated creatinine clearance must be repeated as soon as possible (at most within 1 week) of a value of <50 mL/min, calculated using the following formula:

{([140 - age(yr)]x [weight(kg)])÷[72 x serum Cr(mg/dL)]} x 0.85

Study treatment may be continued while repeating estimated creatinine clearance levels as long as the participant is asymptomatic.

- For confirmed level of <50 mL/min, all antiretroviral agents should be held. **Alternatively, in discussion with the clinical management committee, TDF can be held, or another NRTI such as ZDV, d4T or abacavir can be substituted for TDF with appropriate renal dosing adjustments while the etiology of the renal insufficiency is being investigated and renal function is being closely followed.**
- **Participants with a confirmed level of <50 mL/min should undergo a thorough evaluation for potential causes of decreased renal function in addition to receiving treatment, as appropriate. If TDF is the only potential cause of renal insufficiency found, then TDF should be permanently discontinued.**
- Participants should be followed as medically indicated until the estimated creatinine clearance level returns to ≥60 mL/min, after which **TDF-containing ART may be restarted with careful monitoring of renal function if the renal insufficiency was ascribed to other etiologies rather than to TDF**. If the estimated creatinine clearance remains <60 mL/min, ART may be resumed with best available treatment without TDF.

Participants who are receiving FTC/TDF and must permanently discontinue TDF may continue to receive FTC and may substitute another NRTI for TDF.

7.5.4 Diarrhea

Diarrhea is a common side effect of HIV infection and of study treatment (in particular of LPV/RTV and ddI), and often subsides after several weeks of antiretroviral treatment. If no infectious cause of diarrhea is found and onset is temporally related to new medication, symptomatic management with antidiarrheal agents is appropriate.

Grade ≥3

- If Grade ≥3 diarrhea persists beyond 14 days of symptomatic management, then all study treatment should be held.
- Upon resolution to Grade ≤2 or to the pre-entry/entry value, then restart study treatment at full doses.
- If Grade ≥3 diarrhea recurs upon the resumption of study treatment despite symptomatic management, study treatment should again be interrupted and alternative antiretroviral agents should be considered in consultation with the A5208/OCTANE CMC.

7.5.5 Nausea (with or without vomiting)

Although common, nausea following initiation of HIV therapy with antiretroviral medications usually subsides or resolves during the first few weeks of treatment.

Grade 1 - 2

- Continue study treatment without interruption and treat symptomatically with permitted oral antiemetic therapies or antiemetic suppositories.
- Participants should be instructed to take medications with food (with the exception of ddI, among the few participants who may receive ddI).
- Investigators should consider the diagnoses of pancreatitis or lactic acidosis syndrome, and evaluate participants for these events if clinically indicated.

Grade ≥3

- Hold all study drugs and treat symptomatically.
- Upon resolution to Grade ≤2 or baseline, restart study treatment at full doses.
- If Grade ≥3 nausea and vomiting recurs upon the resumption of study treatment despite symptomatic management, study treatment should again be interrupted and alternative antiretroviral agents should be considered in consultation with the A5208/OCTANE CMC.
- For participants who experience nausea and vomiting Grade ≥3 related to initiating ZDV, dose reduction of ZDV to 200 mg BID may be used.

7.5.6 Lactic Acidosis

A sometimes fatal syndrome of lactic acidemia / lactic acidosis, often associated with evidence of hepatic steatosis, is a recognized but rare complication of NRTI therapy.

Lactate level greater than the upper limit of normal confirmed by repeat lactate level analysis may be part of a syndrome referred to as lactic acidemia or lactic acidosis. Lactic acidemia refers to the presence of plasma lactate above ULN (confirmed) without evidence of a metabolic acidosis. In addition, lactic acidemia may be symptomatic or symptomatic. Lactic acidosis is a potentially life-threatening condition and presents with elevated plasma lactate level AND an arterial pH less than 7.35, in general with low bicarbonate or increased anion gap. It is usually accompanied by symptoms which may be vague and/or subtle.

The lactic academia/lactic acidosis syndrome is felt to be secondary to mitochondrial toxicity induced by the inhibitory effect of NRTIs on DNA polymerase gamma, a key enzyme needed for mitochondrial DNA synthesis. Current knowledge regarding this syndrome is incomplete. Obesity, female gender, and prolonged NRTI exposure may be risk factors. Symptoms of lactic acidosis frequently involve nonspecific symptoms such as fatigue, weakness, and fever, but in the majority of cases also involve symptoms suggestive of hepatic dysfunction such as nausea, vomiting, abdominal or epigastric discomfort, abdominal distension, hepatomegaly, and new onset elevated liver enzymes. A high index of suspicion may be required to diagnose this condition. Alternatively, it is possible that unwarranted concern may be raised by over interpretation of lactic acid levels. NRTI toxicity is only one cause of lactic acidosis. Lactic acid elevations are also seen in the context of diabetes mellitus, uremia, liver disease, severe infections, malignancies, alkaloses, and drug and toxin ingestion of such substances as ethanol, methanol, ethylene glycol, and salicylates.

The following case definitions of symptomatic and asymptomatic lactic acidosis will be used in A5208/OCTANE:

Symptomatic Lactic Acidosis:

New, otherwise unexplained, and persistent occurrence of one or more of the following symptoms:

- - - Nausea and/or vomiting
    - Abdominal pain or gastric discomfort
    - Abdominal distention
    - Increased hepatic transaminase levels
    - Unexplained fatigue
    - Dyspnea
    - Weight loss >5% body weight
    - Muscle weakness

AND

Lactate level (if available) >2 × ULN confirmed by repeat lactate level analysis (as soon as possible, generally within 1 week). In the absence of lactate levels, serum bicarbonate levels and anion gap should be assessed. The presence of depressed bicarbonate levels or an increased anion gap would suggest the possibility of lactic acidosis.

Asymptomatic Lactic Acidemia/Lactic Acidosis:

Lactate level (if available) >4 × ULN confirmed by repeat lactate level analysis (as soon as possible, generally within 1 week) even in the absence of the symptoms listed above.

Symptomatic and Asymptomatic Lactic Acidemia/Lactic Acidosis:

- Discontinue study treatment
- Repeat lactate levels every 4 weeks until the level returns to <2 x ULN
- Upon resolution to <2 x ULN, a new antiretroviral regimen may be initiated in consultation with the A5208/OCTANE CMC.

7.5.7 Lipase Elevations and Pancreatitis

The diagnosis of pancreatitis will be reported as a clinical finding (i.e., symptomatic pancreatitis). The enzyme abnormality that will be used for helping to make the diagnosis of pancreatitis is the lipase level.

For symptomatic (gastrointestinal symptoms, particularly abdominal pain) elevations in lipase of the following grades:

Grade <1

- Search for other causes of symptoms. If none are found and symptoms persist, repeat lipase within 2 weeks.

Grade ≥1

- Follow participants and repeat lipase as soon as possible (within 1 week is optimal).

Grade ≤2

- If repeat lipase is Grade ≤2 and symptoms persist, then exclude other possible diagnoses (e.g., renal insufficiency causing false elevations in lipase).
- If no other diagnosis is found, then diagnose as clinical pancreatitis, and hold all study treatment.

Grade ≥3

- If repeat lipase is Grade ≥3, hold all study treatment and follow at least weekly until serum lipase returns to Grade ≤2.
- Upon resolution to Grade ≤2, a new antiretroviral regimen may be initiated in consultation with the A5208/OCTANE CMC.
  - ddI should be permanently discontinued after occurrence of a Grade ≥3 lipase in a participant taking ddI.
- Diagnose as clinical pancreatitis. The severity of clinical pancreatitis will be recorded as the highest severity level of any of the associated clinical signs and symptoms.

For asymptomatic elevations in lipase (elevations without abdominal pain or nausea):

Grade <1:

- Search for other causes of elevated lipase. If none are found and elevated lipase persists, repeat lipase within 2 weeks.

Grade ≥1:

- Follow participants and repeat lipase as soon as possible (within 1 week is optimal).

Grade 2

- If repeat lipase is Grade 2, then exclude other possible diagnoses (e.g., renal insufficiency causing false elevations in lipase).
- If no other diagnosis is found, and no symptoms occur, then follow lipase every 2 weeks until Grade <1. No other action need be taken.

Grade ≥3

- If repeat lipase is Grade ≥3, hold all study treatment and follow at least weekly until serum lipase returns to Grade ≤2.
- Upon resolution to Grade ≤2, a new antiretroviral regimen may be initiated in consultation with the A5208/OCTANE CMC.
  - ddI should be permanently discontinued after occurrence of a Grade ≥3 lipase in a participant taking ddI.

If a participant becomes symptomatic, then the directions regarding symptomatic elevations in lipase above should be followed.

Upon reinitiation, lipase determinations should be performed monthly. Any elevation of lipase of Grade ≥3 or any recurrence of symptoms during this period will lead to a re-evaluation and permanent discontinuation of the suspected study drugs(s).

7.5.8 Hypertriglyceridemia

For triglyceride levels >1200 mg/dL in asymptomatic participants, determine levels of plasma lipids after an overnight fast, then follow guidelines for lipase elevations (above).

In participants with isolated hypertriglyceridemia and high LDL cholesterol, weight reduction should be strongly encouraged if obesity is present. Fat intake should be decreased, but the concomitant increase in carbohydrate intake may raise triglyceride and lower HDL levels. Severe hypertriglyceridemia and hyperchylomicronemia require very low fat diets, avoidance of free sugars, and decreased alcohol intake.

Pharmacologic treatment of hypertriglyceridemia is dependent upon local availability of such treatment, and is at the discretion of the site investigator. Treatment should be documented as concomitant drugs on the CRFs. The preferred treatment is with gemfibrozil (600 mg q12h, 30 minutes prior to the morning and evening meals). Niacin (500 mg/day to start and increasing to 4 g/day) produces frequent cutaneous flushing, with or without pruritis. However the cutaneous symptoms tend to subside after several weeks and may be minimized by initiating therapy at low doses. Because of its propensity to worsen the control of blood sugar, niacin should be used with caution in participants with diabetes mellitus or a history of hyperglycemia.

Bile sequestering resins are discouraged because their use is usually associated with increased triglyceride levels. HMG-CoA reductase inhibitors tend more to decrease cholesterol than triglycerides and are not recommended as first-line therapy for hypertriglyceridemia, and some have significant drug interactions with protease inhibitors.

7.5.9 CK Elevation

CK measurements will not be performed routinely as part of the protocol. CK will be measured only if participants develop clinical symptoms consistent with a diagnosis of myositis. For persistent CK elevations >3000 mg/dL (about 20 x ULN), CK should be redrawn after participants abstain from exercise for 24 hours. If CK is still >3000 mg/dL, ZDV should be discontinued and replaced with another NRTI, among the few participants who will be taking ZDV.

7.5.10 Anemia/Neutropenia

Study treatment-related hematologic toxicities should be quite rare among participants receiving the primary Step 1 treatment regimens. A small number of participants may receive ZDV rather than TDF, and it is with regard to these participants that most of this section is relevant.

Grade 2 anemia or neutropenia

- Participants who are taking ZDV may have the ZDV dose reduced to 200 mg BID or may substitute another NRTI for ZDV.

Grade 3 anemia or neutropenia

- For participants taking ZDV, another NRTI may be substituted for ZDV. Alternatively, ZDV may be held until the adverse event returns to Grade ≤2.
- Upon resolution to Grade ≤2, ZDV may be resumed at a reduced dose.
- If the same Grade 3 adverse event recurs on a reduced dose of ZDV, then ZDV should be replaced with another NRTI. The investigator in conjunction with the study team may choose to continue ZDV at a reduced dose in the setting of Grade 3 anemia if the risks of discontinuing the ZDV outweigh the benefits and a switch to an alternative NRTI is not feasible.

Grade 4 anemia or neutropenia

- **Generally,** study treatment **should be interrupted** until the **anemia or neutropenia** returns to Grade ≤2. **However, if the etiology of the anemia or neutropenia is ascribed to factors other than study drug, then study treatment (with the exception of ZDV) may be continued at the discretion of the site investigator and in consultation with the clinical management committee, with close hematologic monitoring.**

7.5.11 CNS Symptoms with EFV

Participants should be informed that EFV may cause dizziness, impaired concentration, and/or drowsiness and instructed that if they experience these symptoms they should avoid potentially hazardous tasks such as driving or operating machinery.

Participants should be informed that these symptoms are likely to improve with continued therapy. Dosing at bedtime improves the tolerability of these symptoms and is recommended during the first weeks of therapy and in participants who continue to experience these symptoms. Those receiving EFV should be alerted to the potential for additive CNS effects when EFV is used concomitantly with alcohol or psychoactive drugs.

There have been reports of delusions and inappropriate behavior, predominantly in participants with a history of mental illness or substance abuse. Severe acute depression has also been infrequently reported in both EFV-treated and control-treated participants. Discontinuation of EFV may be required and substitution of NVP implemented.

Participants who experience adverse events attributable to EFV have the option of reducing their dose from 800 mg QD to 600 mg QD. In the event that a participant experiences treatment-limiting CNS adverse events attributable to EFV, EFV should be discontinued and may be replaced with another drug in consultation with the A5208/OCTANE CMC.

7.5.12 Peripheral Neuropathy

Participants should be monitored for the development of peripheral neuropathy, which is usually characterized by numbness, tingling, or pain in the feet or hands.

Treatment of the peripheral neuropathy is according to the site investigator, but generally begins with non-opioid analgesics, including nonsteroidal anti-inflammatory agents and acetaminophen, and the use of tricyclic antidepressants when more severe pain is present.

Grade 1

- Study treatment may be continued at present dosage. Symptomatic treatment may be provided at the discretion of the site investigator.

Grade 2

- Participant should be managed per site investigator discretion, which may include symptom management, or dose reduction of ddI or temporary cessation of ddI, for the few participants who will be taking this drug in A5208/OCTANE.

- If Grade 2 toxicity resolves to Grade ≤1 within 28 days after a dose reduction in ddI, then the dose of ddI may be continued at the reduced dose or increased back to the initial dose at the discretion of the site investigator.

Grade ≥3

- Permanently discontinue ddI, and substitute another NRTI.
- Symptomatic treatment may be provided at the discretion of the local investigator.

## 7.6 Management of Immune Reconstitution Inflammatory Syndromes

Inflammatory syndromes have been reported to occur shortly after the initiation of potent combination antiretroviral therapy. When these syndromes are suspected the following management plan should be followed. Consultation with the A5208/OCTANE CMC is recommended.

- Continue antiretroviral treatment.
- Confirm diagnosis of opportunistic infection (OI).
- Continue or initiate specific therapy for the infection.
- Evaluate the participant clinically to exclude a new infectious process if the participant was already receiving therapy for the OI.
- Initiate anti-inflammatory agents, initially nonsteroidals or, if needed corticosteroids at the discretion of the site investigator in consultation with the A5208/OCTANE CMC.

# CRITERIA FOR STUDY OR TREATMENT DISCONTINUATION

## 8.1 Criteria for Treatment Discontinuation **(Steps 1 and 2 only)**

- Drug-related toxicity (see section 7.0 Toxicity Management).
- Requirement for prohibited concomitant medications (see section 5.4).
- Failure by the participant to attend 3 consecutive clinic visits.
- Participant repeatedly noncompliant with study treatment as prescribed, as determined by the site investigator.
- Clinical reasons believed life threatening by the site investigator, even if not addressed in the toxicity management of the protocol.

## 8.2 Criteria for Study Discontinuation **(Steps 1, 2, and 3)**

- Request by the participant to withdraw.
- Request of the primary care provider if s/he thinks the study is no longer in the best interest of the participant.
- Participant judged by the investigator to be at significant risk of failing to comply with the provisions of the protocol as to cause harm to self or seriously interfere with the validity of the study results.
- At the discretion of the ACTG, NIAID, IRB or EC, Office for Human Research Protections (OHRP), local ministry of health, investigator, or pharmaceutical supporter.
- Imprisonment or involuntary confinement in a medical facility (e.g., for psychiatric illness or infectious disease)

# 9.0 STATISTICAL CONSIDERATIONS

**Sections 9.1 to 9.6 address statistical considerations for the randomized component of the study including Steps 1 and 2, as described in Version 1.0 of the protocol with amendments included in Versions 2.0 and 3.0. Section 9.7 provides statistical considerations for Step 3, the observational follow-up component that was introduced into the study in Version 4.0 of the protocol.**

## 9.1 General Design Issues **(Steps 1 and 2)**

**This phase III study includes two randomized clinical trials (RCTs) to be undertaken in parallel. Both trials will compare the effect of NNRTI-based versus PI-based ART on the rate of virologic failure in women who have received no prior ART. The two trials will enroll women who, respectively, have or have not previously received SD NVP for the prevention of mother-to-child transmission (MTCT) of HIV.**

**For the RCT that enrolls women who have previously received SD NVP (Trial 1), it is hypothesized that resistance arising from the exposure to SD NVP will lead to superiority of the PI-based therapy over the NNRTI-based therapy. For the RCT that enrolls women with no prior exposure to SD NVP (Trial 2), it is hypothesized (based on trials enrolling study populations that are predominantly male and infected with HIV-1 subtype B) that the effect of NNRTI-based and PI-based therapy will be similar and, hence, that an equivalence trial is appropriate. Originally, Trial 2 was therefore designed to be well-powered to demonstrate equivalence of the two regimens. However, the sample size for Trial 2 (and, consequently, the anticipated median duration of follow-up for Trials 1 and 2) was increased after it was identified that the primary endpoint rate was lower than anticipated. Recognizing that the primary purpose of Trial 2 is to show that NNRTI-based therapy is not inferior to PI-based therapy in the event that PI-based therapy is found to be superior to NNRTI-based therapy among women with prior SD NVP exposure in Trial 1, the revised sample size and duration of follow-up gives high power (close to 90%) in each Trial to answer the question of interest (superiority or non-inferiority). The revised sample size also provides reasonable power (approximately 77%) to demonstrate equivalence of the two regimens.**

**The designs of the two trials are linked. Specifically, Trial 1 aims to establish whether there might be a difference in the rates of virologic failure between NNRTI-based and PI-based therapies among women who have been exposed to SD NVP that is sufficiently large (e.g., a hazard ratio of 2.25) that, if it were due to resistance acquired following use of SD NVP, might affect the desirability of using SD NVP to prevent MTCT of HIV. However, the implication of any difference that is found in Trial 1 needs to be evaluated in the context of the magnitude of difference, if any, between NNRTI- and PI-based regimens in women with no prior exposure to SD NVP. Thus, Trial 2 provides the “control” comparison for the same two regimens that are being compared in Trial 1. As such, Trial 2 has been designed to rule out reasonably large differences in the rate of virologic failure between NNRTI- and PI-based therapies in women with no prior exposure to SD NVP (e.g., a hazard ratio larger than 2.0). The non-inferiority bound and “range of equivalence” in Trial 2 is therefore quite large because of the intrinsic link between the two trials.**

**By designing the study as two parallel RCTs, it is also possible to evaluate whether any difference in the effect of NNRTI-based and PI-based therapy on virologic outcome varies between women with versus without prior SD NVP exposure. Statistically, this involves the evaluation of the interaction of the randomized treatment and prior SD NVP exposure on the rate of virologic failure. The evaluation of this interaction is also well-powered (approximately 70% power). However, it should be noted that this is not a randomized comparison because women participating in this study are not randomized according to use of SD NVP or not. Thus, evaluation of this interaction will also need careful consideration of potential confounding factors in the statistical analysis.**

**The primary endpoint for each of the two trials is time from randomization to virologic failure or death irrespective of the treatment being received at the time of failure. This choice provides for a valid intent-to-treat analysis of virologic outcome comparing the strategies of initiating antiretroviral therapy with either an NNRTI-based regimen or a PI-based regimen. Treatment crossovers (NNRTI to PI, or vice versa) will mean that the difference between strategies will be a little smaller than would have been observed in the artificial situation that women could be maintained on initial therapy despite toxicity. The choice was, however, considered preferable to a primary endpoint of treatment failure (the composite of virologic failure and discontinuation of initial treatment for any other reason) because recent trials have found that the majority of treatment failures, particularly earlier ones, are not virologic and would unlikely be impacted by viral resistance arising from prior SD NVP exposure. It was also considered preferable to an endpoint in which follow-up is censored at the time of initial treatment discontinuation, because this does not provide for a valid intent-to-treat analysis. Because of these difficulties, secondary analyses will be undertaken to evaluate the sensitivity of the conclusions to the choice of endpoint, and these will also be considered in interim analyses in the event that modification or termination of one or both RCTs is considered.**

## 9.2 Endpoints

9.2.1 Primary Endpoint **(Steps 1 and 2)**

Time from randomization to virologic failure or death. For participants whose baseline plasma HIV-1 RNA level is ≤750,000 copies/mL, virologic failure is defined as a plasma HIV-1 RNA level that is <1 log_10_ below baseline 12 weeks after treatment is initiated OR as a plasma HIV-1 RNA level that is ≥400 copies/mL at or after 24 weeks of treatment. For participants whose baseline HIV-1 RNA level is >750,000 copies/mL, virologic failure is defined as a plasma level that is >75,000 copies/mL 12 weeks after treatment is initiated, OR as a plasma HIV-1 RNA level that is ≥400 copies/mL at or after 24 weeks of treatment. These definitions of virologic failure will be applied regardless of whether or not randomized treatment is being taken at the time of failure.

The date of virologic failure will be the date on which the first of two successive failing HIV-1 RNA measurements was obtained irrespective of the time interval between these measurements (though the intent is that the confirmation measurement will be obtained within 2 to 4 weeks after the initial measurement). The baseline value will be the value obtained at the study entry visit. If this value is not available, then the baseline value will be the screening value.

Participants who are lost to follow-up will be censored at the date of the last available HIV-1 RNA measurement, except that participants for whom the last measurement was failing but no confirmatory value was obtained will be considered to be virologic failures.

For the purposes of defining the primary endpoint for Steps 1 and 2, only HIV-1 RNA measurements obtained prior to June 3, 2009 would be considered as initial failing measurements, although a confirmatory measurement may be obtained after June 3, 2009. Similarly, only deaths occurring on or before June 3, 2009 would be considered.

9.2.2 Secondary Endpoints

9.2.2.1 Time from randomization to treatment failure defined as virologic failure (see Section 9.2.1), death, or permanent discontinuation of the NNRTI or PI components of the randomized treatment, whichever occurs first. Changes in the NRTI drugs will not constitute treatment failure.

- - - 1. Drug-resistant plasma virus as determined at virologic failure by bulk sequencing.

9.2.2.3 Tolerability and safety of drug regimens as measured by (a) time from randomization to the first adverse event requiring discontinuation of any of the drugs that formed the initial regimen; (b) time from randomization to the first adverse event requiring discontinuation of the NNRTI or PI component of the initial regimen; and (c) time from randomization to the first Grade 3 or higher adverse event.

9.2.2.4 Genetic relatedness of NNRTI and/or PI failure to baseline minor species variants.

9.2.2.5 Change over time in CD4+ cell count from study entry.

9.2.2.6 Time from randomization to HIV-related disease progression (defined as progression to WHO Clinical Stage 3 or 4) or death.

9.2.2.7 NVP-associated rash or hepatitis (for the NVP PK studies)

9.2.2.8 Adherence to study drug regimens as assessed by participant self-report and pill count.

9.2.2.9 Measures of resource utilization including use of health care facilities and days of work lost.

## 9.3 Randomization and Stratification **(Steps 1 and 2)**

Within each RCT, a dynamic permuted block system to provide balancing within clinical centers will be used to randomize women in approximately equal numbers to receive either the PI-based or NNRTI-based regimen. Within each trial, participants will be stratified using their screening CD4+ cell counts as follows: <50 or ≥50 cells/mm^3^.

## 9.4 Sample Size and Accrual **(Steps 1 and 2)**

**For Trial 1, the original sample size (described in protocol versions 1.0 to 3.0, section 9.4) was derived to provide reasonable power to detect a decrease in the true underlying primary endpoint rate from 40% among women randomized to the NNRTI-based regimen to 20% among women randomized to the PI-based regimen.**

**For Trial 2, the sample size was derived to provide reasonable power to show equivalence assuming that the true underlying primary endpoint rate would be 20% among women participating in either randomized arm of Trial 2 (women without prior SD NVP exposure). During reviews of the study by the DSMB, it was noted that the primary endpoint rate might be lower than that assumed in the sample size calculations. Hence, towards the end of the originally planned accrual, the primary endpoint rate was reviewed by the Study Statisticians. With the agreement of the Chair of the DSMB, the Study Team was then notified that there was reasonable evidence that the primary endpoint rate would likely be lower than anticipated, and the observed rate among women participating in Trial 2 across the two randomized arms pooled was released to the Core Study Team for them to consider the implications of a possible lower rate. The Core Study Team was also informed that the loss to follow-up rate was low (approximately 2% prior to the occurrence of a primary endpoint, with a median follow-up of about a year).**

**The Core Study Team convened a teleconference call to discuss the issue and involved an independent statistician (rather than the Study Statisticians who were aware of interim results by randomized arm). The Core Study Team determined that the primary endpoint rate would be lower than that anticipated (likely in the range of 10-15% among Trial 2 participants) and made the following two recommendations concerning how to respond to this:**

1. **Trial 1: There was general agreement that the study be able to detect an absolute 15% reduction in the proportion of women experiencing a primary endpoint in the NNRTI-based arm (e.g. from 27% to 12%), given that 10-15% (rather than 20%) of the PI-treated arm might experience a primary endpoint. This relative difference is greater, but the absolute difference is lower than outlined in version 3.0 of the protocol (from 40% to 20%). This approach was felt to be clinically relevant and reasonable to all of those present on the call.**

**2. Trial 2: There was a preference to not appreciably widen the range of equivalence for the hazard ratio for the primary endpoint in comparing randomized treatments from what is in version 3.0 of the protocol (i.e., a hazard ratio between 0.5 and 2.0). Based upon a preliminary evaluation of sample size and power, the Core Study Team proposed to increase the sample size in Trial 2 by 100 and anticipated that this could be achieved in 6 months from the end of accrual to the study as currently designed. It was also proposed that follow-up of women enrolled in both Trial 1 and Trial 2 would continue, as in the original study design, for 48 weeks after the last woman is randomized in the study.**

9.4.1 Sample size (Trial 1): RCT enrolling women with prior SD NVP prophylaxis

**A sample size of 240 women (120 on each of the NNRTI-based and PI-based regimens) will provide approximately 90% power to detect a true underlying hazard ratio of 2.25 for the difference between randomized arms in the primary endpoint rate. To aid interpretation of a hazard ratio of 2.25, the following table shows the assumed true underlying cumulative proportion of women in each treatment arm experiencing a primary endpoint at selected follow-up times (see below for further details of the assumptions made):**

|  | **48 weeks** | **72**  **weeks** | **96 weeks** | **120 weeks** | **144 weeks** |
| --- | --- | --- | --- | --- | --- |
| **NVP-based**  **Regimen** | **23%** | **29%** | **35%** | **40%** | **45%** |
| **PI-based Regimen** | **11%** | **14%** | **17%** | **20%** | **23%** |

**The power was estimated using a computer simulation study under the following conditions:**

1. **All 240 women are enrolled as of February 2008 and are followed for a further 72 weeks (representing the 24 weeks for completion of the expanded enrollment to Trial 2 plus the subsequent 48 weeks of follow-up for all participants). The distribution of enrollment times used was that achieved already in the study. This gives a median follow-up time of approximately 120 weeks.**
2. **Among women randomized to the PI-based regimen, the underlying true proportion experiencing the primary endpoint will be 5.6% at week 12, and thereafter primary endpoints will be exponentially distributed with 7.3% (of those still at risk) meeting the primary endpoint in each successive 48 weeks of follow-up. These numbers were based on the observed rate as of December 2007 in Trial 2 pooled across the two randomized arms.**
3. **The underlying true hazard ratio comparing the NNRTI-based regimen to the PI-based regimen is 2.25. This was chosen to give absolute rates at 72 weeks of follow-up of approximately 29% versus 14%, and so an absolute difference of 15% which was considered to be clinically relevant by the Core Study Team. This compares with rates of 40% and 20%, giving an absolute difference of approximately 20% at 72 weeks as used in the original design. Note that a similar sized absolute difference is still assumed but occurring later, at about 120 weeks (see table above).**
4. **Allowance for losses to follow-up (approximately 2% per year) and the effect on power due to interim analyses (which suggests an increase in sample size of no more than 2.5% for the stopping guideline being used) was made by reducing the total number of person-visits achieved by 7.5%.**
5. **The primary analysis used a proportional hazards model to derive a two-sided 95% confidence interval for the hazard ratio describing the difference between randomized treatments. Statistical significance of a difference between treatments would then be claimed if the confidence interval excluded a hazard ratio of 1.0.**

**9.4.2 Sample Size (Trial 2): RCT enrolling women with no prior NVP exposure**

**The NNRTI-based regimen will be considered non-inferior (equivalent) to the PI-based regimen if the two-sided 95% confidence interval for the hazard ratio for virologic failure is entirely below 2.0; equivalence will be established if the same confidence interval is entirely within the range 0.5 to 2.0. Although a hazard ratio of 2.0 for the comparison of NNRTI-based to PI-based therapy might be considered large, it needs to be considered in the context of the hazard ratio of 2.25 that Trial 1 is designed to have high power to detect and which is considered to be clinically relevant. Using this definition, a sample size of 500 women (250 on each of the NNRTI-based and PI-based regimens) will provide approximately 88% power to show non-inferiority of the NNRTI-based regimen (and approximately 77% power to show equivalence within the range 0.5 to 2.0) assuming that there is no true difference between the two regimens.**

**To aid interpretation of a hazard ratio of 2.0, the following table shows the assumed true underlying cumulative proportion of women in each treatment arm experiencing a primary endpoint at selected follow-up times (based on similar assumptions to above for the true rate in the PI-based arm):**

|  | **48 weeks** | **72 weeks** | **96 weeks** | **120 weeks** | **144 weeks** |
| --- | --- | --- | --- | --- | --- |
| **NVP-based**  **Regimen** | **21%** | **26%** | **32%** | **37%** | **41%** |
| **PI-based Regimen** | **11%** | **14%** | **17%** | **20%** | **23%** |

**The power was estimated using a computer simulation study under the following conditions:**

1. **The original target sample size of 400 women is enrolled as of February 2008 and a further 100 women are enrolled during the subsequent six months. Then all 500 women are followed for a further 48 weeks. The distribution of enrollment times used was that achieved already in the study for the initial 400 women and was assumed to be uniform over time for the next 100 women. This gives a median follow-up time of approximately 108 weeks.**
2. **Among women randomized to both the PI-based regimen and the NNRTI-based regimen, the underlying true proportion experiencing the primary endpoint is the same, and will be 5.6% at week 12, and thereafter primary endpoints will be exponentially distributed with 7.3% (of those still at risk) meeting the primary endpoint in each successive 48 weeks of follow-up. These numbers were based on the observed rate as of December 2007 in Trial 2 pooled across the two randomized arms.**
3. **Allowance for losses to follow-up (approximately 2% per year) and the effect on power due to interim analyses (which suggests an increase in sample size of no more than 2.5% for the stopping guideline being used) was made by reducing the total number of person-visits achieved by 7.5%.**

**d. The primary analysis uses a proportional hazards model to derive a two-sided 95% confidence interval for the hazard ratio describing the difference between randomized treatments. Non-inferiority of NNRTI-based versus PI-based therapy will be claimed if the upper bound of this confidence interval is less than 2.0, and equivalence of the two regimens will be claimed if the confidence interval is entirely within the range 0.5 to 2.0.**

9.4.3 Sample size: Evaluation of the treatment by prior SD NVP exposure interaction

**The power of the study to evaluate whether the difference between the NNRTI-based and PI-based regimens differs between women with versus without prior SD NVP exposure was evaluated in the same computer simulation study as described above for Trials 1 and 2. Specifically, using the simulated data across the two trials combined, a proportional hazards model was fitted which included randomized treatment, prior SD NVP exposure (yes or no), and a treatment by prior exposure interaction term. The simulation study showed that the power to detect an interaction is approximately 70% when the true hazard ratio comparing NNRTI-based and PI-based therapy among women with prior exposure (Trial 1) is 2.25 and the among women with no prior exposure (Trial 2) is 1.0, and a two-sided 95% confidence interval is used to evaluate the interaction.**

9.4.4 Sample size: Accounting for the possibility that up to 20% of women in Trial 1 (RCT enrolling women with prior NVP exposure) have not in fact been previously exposed to SD NVP.

Data from Stringer et al. [Stringer, 2003; Stringer, personal communication, 2003] showed that in a NVP program for prevention of MTCT in Zambia, 18 (23%) of 77 women who knew their HIV status and stated that they took SD NVP did not have detectable cord blood NVP levels. This type of study has not yet been replicated elsewhere, although it is currently underway in the HIVNET012 cohort and will also occur soon in an MTCT prevention trial in Botswana. SD NVP was given to a subset of mothers in each of these latter two trials in a non-observed fashion, and mothers were asked whether or not they ingested the NVP.

Potential participants who are asked whether or not they took SD NVP prior to enrollment in A5208/OCTANE (more than 6 months postpartum, by non-maternity staff) are likely to feel much more comfortable being truthful than they would be in the immediate peripartum setting, where they may well be judged negatively for not taking NVP to protect their child. There is little incentive for a potential participant to be untruthful about her NVP exposure in A5208/OCTANE, as she would have access to study enrollment and treatment regardless of her NVP exposure.

To account for the possibility that up to 20% of women in Trial 1 did not in fact take SD NVP, let us hypothesize for a moment that there will be virologic failure rates of 45% (rather than 40%) among women who are truly NVP-exposed and who are assigned to the NVP treatment arm of the study, and 20% among women who are truly NVP-exposed and who are assigned to the LPV/RTV arm of the study, as well as among women who are truly non-exposed whether they are assigned to NVP- or LPV/RTV-containing ART.

If we further assume that 20% of women who enter Trial 1 (and are therefore supposedly NVP-exposed) in fact do not have prior NVP exposure, then among these 20%, when randomized to NVP-containing treatment, the virologic failure rate will be 20% (not 45% because they do not have the NVP exposure).

Thus the true virologic failure rate in Trial 1 among women randomized to NVP-containing treatment in an intent-to-treat analysis would be comprised of 20% of women having a 20% failure rate, and 80% having a 45% failure rate. Therefore
the intent to treat failure rate for the NVP treatment arm would be (.2x20%)+(.8x45%)=40%.

Thus the sample size of 240 in Trial 1, which has been chosen to give high power to detect a difference of 40% for NVP-based therapy vs. 20% for LPV/RTV-based therapy in an intent to treat analysis, allows us to detect a difference of 45% versus 20% among women who are truly NVP-exposed, but allowing for the fact that in the trial some women (i.e. 20%) are not NVP-exposed.

- - 1. Accrual and Replacement of Participants

The anticipated enrollment of women is 50-60 per month so that accrual will be completed in approximately 12 months (after IRB approval). As evaluation of whether the difference in effect of NNRTI-based and PI-based therapy varies between women with versus without prior SD NVP exposure would be confounded if there were substantial differences in enrollment to the two RCTs within individual clinical sites (e.g. due to differences in HIV-1 subtype, co-morbidity, etc.), the sites will be asked to enroll women to the two RCTs at rates that are consistent with the overall accrual goals of the two studies (i.e., in a ratio of approximately 5 women with no prior NVP exposure for every 3 women with prior exposure). This will be monitored by the study team. If at any particular site the number of women enrolled in one RCT versus the other RCT differs by more than 6 women from the ideal ratio of 5:3, then the study team will advise the site to refocus its accrual endeavors to reduce the difference.

If a participant does not start study treatment within 7 days after randomization, then she will be declared off-study and no further follow-up is required. All participants who go off-study without starting study treatment will be replaced for the purposes of accrual.

## 9.5 Monitoring **(Steps 1 and 2)**

This study will be reviewed by a NIAID Division of AIDS sponsored Data and Safety Monitoring Board (DSMB) about every 6 months with a first review of interim data in January 2007, or on a frequency determined by the DSMB, but at least annually until completion of the study. The DSMB will review information concerning accrual, characteristics of participants (including baseline NVP resistance among women in Trial 1), quality and completeness of data collection, retention, adverse events (including deaths) and the primary efficacy data.

The first interim analysis is timed to occur while enrollment is ongoing. An important issue to be considered at this interim analysis concerns an evaluation of the available information about SD NVP exposure among women in Trial 1 and the lack of exposure among women in Trial 2. This is to ensure that there is not excessive cross-contamination of the two trial populations according to SD NVP exposure. Note that it is anticipated that some documentation of exposure may not be available at the time of randomization but may be accessible subsequently. Furthermore, data from other MTCT prevention studies in which maternal adherence to SD NVP is being verified will be monitored. A non-exposure rate above 20% in Trial 1 (or data from other studies suggesting very high non-adherence rates to SD NVP) would be cause for concern, and might suggest the need for increasing the sample size and/or being more rigorous about requiring documentation of NVP exposure prior to randomization.

At all interim analyses, unless there are serious concerns about mortality or treatment toxicities, decisions about whether to continue, modify or terminate either or both of the trials will be based primarily on results for the study’s primary endpoint. In evaluating primary endpoint data, a repeated confidence interval (RCI) approach will be used. Because it is unlikely that this trial will be replicated and because the public health policy implications of this trial may be wide-ranging, it is recommended that a very high level of evidence be obtained in support of the study’s conclusions before early termination of one or both of the trials based on the primary endpoint data be considered. For this reason, the RCI will be calculated using a Peto-Haybittle type rule, so that decisions at interim analyses will be guided by the use of a two-sided CI calculated with nominal 99.9% coverage.

In reviewing results of interim analyses, particular consideration should be given to the possibility of differences in outcome in Trial 1 (women with prior SD NVP exposure) according to the level of documentation for the use of NVP and the duration of time since NVP exposure. In addition, particular consideration should be given to the consistency of results for the primary endpoint of virologic failure and the secondary endpoint of treatment failure (section 9.2.2.1), and to the possibility that any difference between regimens is transient. Furthermore, in evaluating equivalence, consideration will be given to the consistency of the intent-to-treat and as-treated analyses.

The following guidelines for various scenarios that might be encountered at interim analyses are provided to assist the DSMB in their review of the results of interim analyses of the primary endpoint data based upon the RCI for the hazard ratio for virologic failure in comparing NNRTI-based to PI-based therapy in each of the two trials. Notwithstanding these guidelines, the DSMB might make alternative recommendations based on their assessment of the totality of evidence from the two trials and any relevant data from other studies. There are two principles underlying the guidelines for monitoring:

(i) The first is that if, at any interim analysis of either trial, the RCI excludes a hazard ratio of one, indicating superiority of one regimen over the other regimen in the trial concerned, then the DSMB might recommend termination of that trial (but note the potential exception in (Scenario C) below). If superiority of either regimen has not been established in the other trial, then the DSMB might recommend continuation of the other trial.

1. The range of equivalence used in the design of Trial 2 is wide (a hazard ratio from 0.5 to 2.0) reflecting the primary role of that trial in providing a “control” comparison of NNRTI- and PI-based regimens in Trial 1, and the interest in detecting quite large effects in Trial 1. Because of this, it is considered reasonable for the DSMB to recommend continuation of either trial to establish equivalence to a much narrower tolerance (i.e. requiring the RCI to be entirely between 0.75 and 1.333; akin to the definition used in ACTG 5095, a trial that is comparing antiretroviral regimens in antiretroviral-naïve adults in the U.S.) except as laid out in Scenario A below.

Based upon these principles, the following scenarios provide guidelines which might be used as a basis for decision-making by the DSMB:

### Scenario A: If Trial 1 (women with prior SD NVP exposure) is or has been previously terminated showing that PI-based therapy is superior to NNRTI-based therapy, then the DSMB might recommend continuation of Trial 2 until equivalence is established (using the requirement that the RCI is entirely within the range 0.5 to 2.0) AND it is demonstrated that the difference in effect of NNRTI- and PI-based regimens varies significantly between Trials 1 and 2. This would provide strong evidence that the effect of the NNRTI-based therapy versus the PI-based therapy is reduced among women with prior exposure to SD NVP as hypothesized for this study and the primary objectives of the study would have been met.

Scenario B: If Trial 2 (women without prior NVP exposure) is or has been previously terminated showing that NNRTI-based therapy is superior to PI-based therapy, then the DSMB might recommend continuation of Trial 1 until it is demonstrated that the NNRTI-based therapy is not inferior to PI-based therapy, where demonstration of non-inferiority is defined as requiring that the upper bound of the RCI for the hazard ratio of virologic failure be less than 1.333. This would establish that NNRTI-based therapy is a reasonable choice for treatment for ART-naïve women regardless of prior exposure to SD NVP.

Scenario C: If Trial 1 shows that NNRTI-based therapy is superior to PI-based therapy, or if Trial 2 shows that PI-based therapy is superior to NNRTI-based therapy, then careful consideration will need to be given to the totality and consistency of evidence from both trials before making a decision about modifying or terminating either or both trials (despite the finding of superiority in one trial). In both of these situations, there may not be a qualitative difference about which regimen should be preferred between women with and without prior exposure to SD NVP based upon the hypothesized effect of resistance related to prior exposure, and so other considerations may be important.

Scenario D: If neither trial has shown superiority of a regimen, then the DSMB might recommend continuation of both trials unless there is very strong and consistent evidence of equivalence of the two regimens in the two trials. Such evidence might be that the RCI for the hazard ratio in each trial is entirely within the range 0.5 to 2, and the RCI for the hazard ratio in the two trials combined is entirely within the range 0.75 to 1.333. The requirement for consistency of evidence from the two trials might also involve the need for the estimated differences between NNRTI-based and PI-based therapy being in the same direction in both trials so that there is no concern that there may be a qualitative difference in which regimen might be preferred according to whether or not a woman had previously received SD NVP.

## 9.6 Analyses **(Steps 1 and 2)**

For the primary endpoint of time from randomization to virologic failure or death, the Kaplan-Meier method will be used to describe the cumulative proportion of participants experiencing failure by time. Cox proportional hazards models will be used to compare the hazard of failure between the NNRTI-based and PI-based regimens, and to construct the associated repeated confidence intervals. Sensitivity analyses will be undertaken to evaluate whether the handling of deaths and the censoring of follow-up for women who are lost to follow-up might affect the strength of the conclusion that can be drawn from the intent-to-treat analysis of the primary endpoint defined in section 9.2.1. As-treated analyses will also be undertaken whereby follow-up is censored at the time that a woman discontinues the randomized NNRTI or PI therapy. In addition, if there is strong documentation that a woman was misclassified at the time of randomization to Trial 1 or Trial 2 according to prior SD NVP exposure, then additional as-treated analyses will be undertaken with (a) these women removed from the respective trial population, and (b) these women reassigned to the trial which corresponds to their documented exposure. Consistency of the results of the intent-to-treat and as-treated analyses will be important in evaluating evidence for superiority or equivalence of the two regimens.

Similar methods will be used for the secondary endpoints concerning time to treatment failure (section 9.2.2.1) and to the safety and tolerability events listed in section 9.2.2.3.

Full details of the proposed analyses will be described in a statistical analysis plan that will be developed once enrollment to the study begins and prior to the commencement of analyses for the first review of these analyses by the DSMB.

## **9.7 Statistical Considerations for Step 3**

- - 1. **General Design Issues (Step 3)**

**Step 3 was introduced in Version 4.0 of the protocol. It is an observational study of outcomes among study participants as they transition from treatment and care provided by the study to locally provided treatment and care. As such, the study is primarily descriptive and hypothesis generating.**

- - 1. **Outcome Measures (Step 3)**

**A subset of efficacy and safety outcome measures obtained during Steps 1 and 2 will be evaluated during Step 3. The following two outcomes measures are considered as primary measures for describing the effects of transitioning to treatment and care provided locally:**

- **Whether or not HIV-1 RNA is <400 copies/mL at Step 3 week 12**
- **Whether or not HIV-1 RNA is <400 copies/mL at Step 3 week 72**
- **Time from the start of locally provided antiretroviral therapy in Step 3 until the change in one or more drugs in that regimen.**
  - 1. **Randomization and Stratification (Step 3)**

**There is no randomization or stratification in Step 3.**

- - 1. **Sample Size and Accrual**

**The sample size for Step 3 will be determined by the number of participants in Steps 1 and 2 who are still in follow-up as of June 4, 2009 who elect to enroll in Step 3. As the rate of death and loss to follow-up in Trial 1 following the DSMB’s recommendation to release results from that part of the study was low, approximately 5%, it is expected that in excess of 90% of participants, i.e., more than about 665 participants, enrolled in Steps 1 and 2 would be eligible for Step 3. Based on discussions with site investigators, it is anticipated that most of these eligible participants would in fact enroll.**

**If the number who enrolled in Step 3 was 600, then the width of a 95% confidence interval around the observed proportion of participants with an HIV-1 RNA <400 copies/mL at week 12 (or at week 72) or the cumulative proportion of particpants with treatment changes at these times would be approximately ±2.4% if the observed rate was 10%, ±3.5% if the rate was 25%, and maximally ±4.0% if the rate was 50%. Even with modest rates of loss to follow-up (e.g. 10%), there would still be good precision (maximally ±4.2%) to describe rates of the primary outcomes measures following the transition to locally provided treatment and care.**

**9.7.5 Monitoring (Step 3)**

**As Step 3 is an observational study among participants receiving locally provided treatment and care, Step 3 follow-up will not be monitored by the DSMB. Instead, the study team will monitor the conduct of Step 3, specifically retention and completeness of data collection at Step 3 weeks 12 and 48. In addition, it is planned to undertake analyses and disseminate the results of the Step 3 week 12 follow-up shortly after Step 3 week 12 data are available for all Step 3 participants.**

**9.7.6 Analyses (Step 3)**

**The analyses of Step 3 will primarily be descriptive, using simple summary statistics to describe cross-sectionally short-term outcomes at Step 3 week 12 and longer-term outcomes at Step 3 week 72 (e.g., proportion with HIV-1 RNA <400 copies/mL and mean CD4 count) as well as distributions of time to events (e.g., time from Step 3 entry to change in one or more antiretroviral drugs). Exploratory hypothesis-generating analyses will be undertaken to evaluate factors that might be associated with these outcomes, such as whether or not one or more drugs had to be changed in transitioning from study-provided to locally provided treatment, or the availability or not of HIV-1 RNA testing as part of local care. These comparisons will use linear, logistic and proportional hazards models as appropriate. A more detailed analysis plan will be developed after enrollment to Step 3 is completed and when the characteristics of the locally provided treatment and care programs are obtained but prior to the start of analyses of follow-up data. It is intended that analyses of Step 3 week 12 follow-up will be undertaken when Step 3 week 12 follow-up is completed for all Step 3 participants (hence, while follow-up to Step 3 week 72 is ongoing).**

# 10.0 DATA COLLECTION AND MONITORING AND ADVERSE EXPERIENCE REPORTING

## 10.1 Records to Be Kept

Case report forms (CRFs) will be provided for each participant. Participants must not be identified by name on any CRFs. Participants will be identified by the patient identification number (PID) and study identification number (SID) provided by the ACTG Data Management Center upon registration and randomization.

## 10.2 Role of Data Management

10.2.1 Instructions concerning the recording of study data on CRFs will be provided by the ACTG Data Management Center. Each A5208/OCTANE site is responsible for keying the data in a timely fashion.

10.2.2 It is the responsibility of the ACTG Data Management Center to assure the quality of computerized data for each ACTG study. This role extends from protocol development to generation of the final study databases.

## 10.3 Clinical Site Monitoring and Record Availability

10.3.1 Site monitors under contract to the National Institute of Allergy and Infectious Diseases (NIAID) will visit participating clinical research sites to review the individual participant records, including consent forms, CRFs, supporting data, laboratory specimen records, and medical records (doctors’ progress notes, nurses’ notes, participants’ hospital charts), to ensure protection of study participants, compliance with the protocol, and accuracy and completeness of records. The monitors also will inspect sites’ regulatory files to ensure that regulatory requirements are being followed and sites’ pharmacies to review product storage and management.

10.3.2 The investigator will make study documents (e.g., consent forms, drug distribution forms, CRFs) and pertinent hospital or clinic records readily available for inspection by the local IRB, the site monitors, the NIAID, local ministry of health, the Office for Human Research Protections (OHRP), the pharmaceutical supporters or their designees for confirmation of the study data.

## 10.4 Expedited Serious Adverse Event (EAE) Reporting

The Expedited Adverse Events (EAE) reporting guidelines and definitions for A5208/OCTANE and the methods for reporting AEs to the DAIDS Safety Office through the Regulatory Compliance Center (RCC) are contained in the Manual for Expedited Reporting of Adverse Events to DAIDS, dated May 6, 2004. The DAIDS EAE Manual is available on the RCC website: <http://rcc.tech-res-intl.com/eae.htm>.

**Sites using the DAIDS Adverse Events Reporting System (DAERS) internet-based reporting system for submission of EAEs to DAIDS will follow the DAERS processes as outlined in the DAERS training information. For questions about DAERS, please contact DAIDS-ES at** [**DAIDS-ESSupport@niaid.nih.gov**](mailto:DAIDS-ESSupport@niaid.nih.gov) **or from within the DAERS application itself. Questions about EAE reporting should be directed to the RCC.**

**If the site cannot use DAERS to report an AE on an expedited basis,** the AE must be documented on the DAIDS EAE Form and submitted to the DAIDS Safety Office through the RCC ([RCCSafetyOffice@tech-res.com](mailto:RCCSafetyOffice@tech-res.com) or Fax (301) 897-1710).

In addition to submitting EAE information to the DAIDS Safety Office through the RCC, the site investigator is required to submit AE information as required by local regulatory or other local authority.

This protocol follows targeted reporting requirements. The study agents that must be considered in determining the relationships of AEs requiring expedited reporting to DAIDS are: NVP, LPV/RTV, FTC, TDF, FTC/TDF, ddI EC, EFV, and ZDV. AEs must be reported on an expedited basis at the targeted reporting level during the protocol-defined EAE reporting period which is the entire **Step 1 and/or Step 2** study duration for an individual participant (from study enrollment until study completion or discontinuation **of Step 1 or 2**).

The Division of AIDS Table for Grading the Severity of Adult and Pediatric Adverse Events, Version 1.0, December 2004 can be found on the RCC Web site: <http://rcc.tech-res-intl.com/eae.htm>.

After the end of the protocol-defined EAE Reporting Period stated above, sites must report serious, unexpected, clinical suspected adverse drug reactions **to drugs administered in Step 1 or 2** if the study site staff becomes aware of the event on a passive basis, i.e., from publicly available information. **EAE reporting will not be required in Step 3, other than as related to study drug provided in Step 1 or 2, as above.**

# 11.0 HUMAN PARTICIPANTS

## 11.1 Institutional Review Board (IRB) Review and Informed Consent

This protocol and the informed consent documents (Appendices I and II) and any subsequent modifications will be reviewed and approved by the IRB or ethics committee responsible for oversight of the study. A signed consent form will be obtained from the participant (or parent, legal guardian, or person with power of attorney for participants who cannot consent for themselves, such as those below the legal age). The consent form will describe the purpose of the study, the procedures to be followed, and the risks and benefits of participation. A copy of the consent form will be given to the participant, parent, or legal guardian, and this fact will be documented in the participant’s record.

## 11.2 Participant Confidentiality

All laboratory specimens, evaluation forms, reports, and other records that leave the site will be identified by coded number only to maintain participant confidentiality. All records will be kept locked. All computer entry and networking programs will be done with coded numbers only. Clinical information will not be released without written permission of the participant, except as necessary for monitoring by IRB, the NIAID, local ministry of health, the OHRP, the pharmaceutical supporters, or their designees.

## 11.3 Study Discontinuation

The study may be discontinued at any time by the pharmaceutical supporters, local ministry of health, the IRB or EC, the OHRP, the NIAID, or other government agencies as part of their duties to ensure that research participants are protected.

# PUBLICATION OF RESEARCH FINDINGS

Publication of the results of this trial will be governed by ACTG policies. Any presentation, abstract, or manuscript will be made available for review by the pharmaceutical supporters prior to submission.

# BIOHAZARD CONTAINMENT

As the transmission of HIV and other blood-borne pathogens can occur through contact with contaminated needles, blood, and blood products, appropriate blood and secretion precautions will be employed by all personnel in the drawing of blood and shipping and handling of all specimens for this study, as currently recommended by the Centers for Disease Control and Prevention and the National Institutes of Health.

All dangerous goods materials, including diagnostic specimens and infectious substances, must be transported according to the instructions detailed in the International Air Transport Association (IATA) Dangerous Goods Regulations.

# 14.0 REFERENCES

Antinori A, Zaccarelli M, Cingolani A, et al., Cross-resistance among nonnucleoside reverse transcriptase inhibitors limits recycling efavirenz after nevirapine failure. AIDS Res Hum Retroviruses 2002; 18: 835-8.

Apetrei C, Descamps D, Collin G, et al. Human immunodeficiency virus type 1 subtype F reverse transcriptase sequence and drug susceptibility. J Virol. 1998 May;72(5):3534-8.

Autran B, Carcelain G, Li TS, et al. Positive effects of combined antiretroviral therapy on CD4+ T cell hoeostatis and function in advanced HIV disease. Science. 1997 Jul 4;277(5322):112-6.

Barreiro P, Soriano V, Casas E, et al. Prevention of nevirapine-associated exanthema using slow dose escalation and/or corticosteroids. AIDS. 2000 Sep29;14(14):2153-7.

Beckerman KP. Long term findings of HIVNET 012: The Next Steps. Lancet 2003; 362;842-3.

Blum MR, Begley J, Zong J, et al. Lack of pharmacokinetic interaction between emtricitabine and tenofovir DF when co-administered to steady state in healthy volunteers [Abstract]. Presented at the 43^rd^ Interscience Conference on Antimicrobial Agents and Chemotherapy; 2003 September 14-17; Chicago, IL. Abstract A-1621.

Cahn P, Raffi F, Saag M et al. Virologic efficacy and patterns of resistance mutations in ART-naïve patients receiving combination therapy with daily (QD) emtricitabine compared to twice-daily (BID) stavudine in a randomized, double-blind, multicenter clinical trial. In Program and Abstracts of the 10^th^ Conference on Retroviruses and Opportunistic Infections. 2003; Boston, MA [Abstract 606].

Cheng A, Wulfsohn M, Cheng SS, Toole JJ. Two year long-term safety profile of tenofovir DF in treatment-experienced patients from randomized, double-blind, placebo-controlled clinical trials. 9^th^ European AIDS Conf; 2003 Oct 25-29; Warsaw, Poland. Abstract 156.

Collins KL. How HIV evades CTL recognition. Curr HIV Res. 2003, Jan;1(1):31-40. Review.

Cozzi-Lepri A, Phillips A, d'Arminio Monforte A, et al. Italian Cohort Naive Antiretrovirals (I.Co.N.A.) Study Group. Virologic and immunologic response to regimens containing nevirapine or efavirenz in combination with 2 nucleoside analogues in the Italian Cohort Naive Antiretrovirals (I.Co.N.A.) study. J Infect Dis. 2002;185:1062-9.

CPMP Working Party on Efficacy of Medicinal Products for Guidance III/3630/92-EN. Biostatistical methodology in clinical trials in applications for marketing authorizations for medicinal products. Stat Med. 1995;14:1659-82.

D’Aquila RT, Hughes MD, Jonson VA, et al. Nevirapine, zidovudine, and didanosine compared with zidovudine and didanosine in patients with HIV-1 infection. A randomized, double-blind, placebo-controlled trial. AIDS Clinical Trials Group Protocol 241 Investigators. Ann Intern Med. 1996 Jun 15;124(12):1019-30.

Descamps D, Apetrei C, Collin G, et al. Naturally occurring decreased susceptibility of HIV-1 subtype G to protease inhibitors. AIDS. 1998 Jun 18;12(9):1109-11.

Erickson DA, Mather G, Trager WF, et al. Characterization of the in vitro biotransformation of the HIV-1 reverses transcriptase inhibitor nevirapine by human hepatic cytochromes P-450. Drug Metab Dipos. 1999 Dec;27(12):1488-95.

Eshleman SH. Analysis of nevirapine resistance seven days after single-dose nevirapine prophylaxis: HIVNET 012. 10th Conference on Retroviruses and Opportunistic Infections. February 10-12, 2003, Boston, MA. Abstract 856.

Eshleman SH, Becker-Pergola G, Deseyve M, et al. Impact of human immunodeficiency virus type (hiv-1) subtype on women receiving single-dose nevirapine prophylaxis to prevent hiv-1 vertical transmission (HIV Network for Prevention Trials 012 study). J Infect Dis. 2001;184(7):914-7.

Eshleman SH, Guay LA, et al. Characterization of nevirapine (NVP) resistance mutations in women with subtype A vs. D HIV-1 6-8 weeks after single dose NVP (HIVNET 012). J Acquir Immune Defic Syndr 2003; 35:126-30.

Eshleman SH, Jones D, et al. HIV-1 variants with diverse nevirapine resistance mutations emerge rapidly after single-dose nevirapine: HIVNET 012. Antiviral Ther 2003;8: S86.

Eshleman SH, Mracna M, Guay LA, et al., Selection and fading of resistance mutations in women and infants receiving nevirapine to prevent HIV-1 vertical transmission (HIVNET 012). AIDS. 2001;15:1951-7.

Feng JY, Anderson KS. Mechanistic studies comparing the incorporation of (+) and (-) isomers of 3TCTP by HIV-1 reverse transcriptase. Biochemistry 1998; 38:55-63.

Feng JY, Shi J, Schinazi RF, Anderson KS. Mechanistic studies show that (-)-FTC-TP is a better inhibitor of HIV-1 reverse transcriptase than 3TC-TP. The FASEB J 1999; 13:1511-1517.

Feng JY, 14^th^ International Conference on Antiviral Research 2001, Abstract 65.

Frater AJ, Beardall A, Ariyoshi K, et al. Impact of baseline polymorphisms in RT and protease on outcome of highly active antiretroviral therapy in HIV-1-infected African patients. AIDS. 2001 Aug 17;15(12):1493-502.

Gallant JE, Staszewski S, Pozniak AL, et al. Long-term efficacy and safety of tenofovir DF (TDF): A 144 week comparison versus stavudine (d4T) in antiretroviral-naïve patients. XV International AIDS Conference; 2004 July 11-16; Bangkok, Thailand.

Gathe J, Podzamczer D, Johnson M, et al. Once-daily vs. twice-daily lopinavir/ritonavir in antiretroviral-naïve patients: 48-week results [Poster Number 570]. 11^th^ Conference on Retrovirus and Opportunistic Infections (CROI); 2004 February 8-11; San Francisco, CA.

Gazzard B, DeJesus E, Campo R, et al. The combination of tenofovir DF (TDF), emtricitabine (FTC) and efavirenz (EFV) has significantly greater response vs fixed dose zidovudine/lamivudine (CBV) and EFV in antiretroviral naive patients: a 24 week preliminary análisis (abstract H1137c). 44th Interscience Conference on Antimicrobial Agents and Chemotherapy. October 30-November 2, 2004, Washington, DC.

Gerondelis P, Archer RH, Palaniappan C, et al. The P236L delavirdine-resistant human immunodeficiency virus type 1 mutant is replication defective and demonstrates alterations in both RNA 5’-end- and DNA 3’-end-directed RNase H activities. J Virol. 1999 Jul;73(7):5803-13.

Gomes P, Abecasis A, Almeida M, et al. Transmission of HIV-2. Lancet Infect Dis. 2003; 3(11):683-4.

Guay LA, Musoke P, Fleming T, et al. Intrapartum and neonatal single-dose nevirapine compared with zidovudine for prevention of mother-to-infant transmission of HIV-1 in Kampala, Uganda: HIVNET-012 randomised trial. Lancet 1999; 354:795
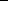
802.

Gulick RM, Mellor JW, Havlir D, et al. Treatment with indinavir, zidovudine, and lamiduvine in adults with human immunodeficiency virus infection and prior antiretroviral therapy. N Engl J Med. 1997 Sep 11;337(11):734-9.

Hammer SM, Squires KE, Hughes MD, et al. A controlled trial of two nucleoside analogues plus indinavir in persons with human immunodeficiency virus infection and CD4 cell counts of 200 per cubic millimeter of less. AIDS Clinical Trials Group 320 Study Team. N Engl J Med. 1997 Sep 11;337(11):725-33.

Hammer SM, Vaida FL, Bennett KS, et al.  Dual versus single protease inhibitor therapy following antiviral treatment failure: A randomized trial.  JAMA 2002; 288:169-180.

Harris M, Yip B, Zalunardo N, et al. 2^nd^ IAS Conference on HIV Pathogenesis and Treatment; July 13-16, 2003; Paris. Abstract 55.

Havlir D. Nevirapine-resistant human immunodeficiency virus: kinetics of replication and estimated prevalence in untreated patients. J Virol. 1996 Nov;70(11):7894-9.

Imamichi H, Crandall KA, Natarajan V, et al. Human immunodeficiency virus type 1 quasi species that rebound after discontinuation of highly active antiretroviral therapy are similar to the viral quasi species present before initiation of therapy. J Infect Dis. 2001;183(1):36-50.

Imperiale SM, Lanes SZ, Stern JO, et al. The Viramune (nevirapine) hepatic safety project: analysis of symptomatic hepatic events. Antiviral Ther. 2002;7:L57.

Jackson JB, Musoke P, Fleming T, et. al., Intrapartum and neonatal single dose nevirapine compared with zidovudine for prevention of mother-to-child transmission of HIV-1 in Kampala, Uganda: 18 month follow-up of the HIVNET 012 randomised trial. Lancet 2003; 362: 859-68.

Jourdain G, Ngo-Giang-Huong N, Le Coeur S, et al. Intrapartum exposure to nevirapine and subsequent maternal responses to nevirapine-based antiretroviral therapy. N Engl J Med. 2004;351(3):229-40.

Jourdain G, Ngo-Giang-Huong N, Tungyai P, et al. Exposure to intrapartum single-dose nevirapine and subsequent maternal 6-month response to NNRTI-based regimens. 11th Conference on Retroviruses and Opportunistic Infections. 2004; San Francisco, CA. Abstract.

Kantor R, Lee E, et al. Rapid flux in nonnucleoside reverse transcriptase mutations among subtype C HIV-1-infected women after single dose nevirapine. Antiviral Ther 2003; 8: S85.

Kantor R, Zijenah LS, Shafer RW, et al. HIV-1 subtype C reverse transcriptase and protease genotypes in Zimbabwean patients failing antiretroviral therapy. AIDS Res Hum Retroviruses. 2002 Dec 10;18(18):1407-13.

Kearney et al., 12th Antiviral Drug Resistance Workshop, June 2003; Cabo San Lucas, Mexico. Abstract 86.

Kearney BP, Zong J, Begley J, et al. Bioequivalence of combination tenofovir DF/emtricitabine tablets for one-pill once daily administration [Poster No. 7.3]. 5^th^ International Workshop on Clinical Pharmacology of HIV Therapy; 2004April 1-3; Rome, Italy.

King M, Bernstein B, Cernohous P, et al. Impact of baseline CD4 cell count and viral load on durability of virologic response through 96 weeks for lopinavir/ ritonavir and nelfinavir in a phase III clinical trial. 9th Conference on Retroviruses and Opportunistic Infections. 2002; Seattle, WA. Abstract 470-M.

Lallemant M, Jourdain G, et al., and the Perinatal HIV Prevention Trial (Thailand). Randomized, double-blind trial assessing the efficacy of single-dose perinatal nevirapine added to a standard zidovudine regimen for the prevention of mother-to-child transmission of HIV-1 in Thailand. 11th Conference on Retroviruses and Opportunistic Infections. 2004; San Francisco, CA. Abstract

Lamson MJ, Sabo JP, MacGregor TR, et al. Single dose pharmacokinetics and bioavailability of nevirapine in healthy volunteers. Biopharm Drug Dispos. 1999 Sep;20(6):285-91.

Lecossier D, Bouchonnet F, Clavel F, Hance AJ. Hypermutation of HIV-1 DNA in the absence of the Vif protein. Science. 2003 May 16;300(5622)1112.

Little SJ, Holte S, Routy JP, et al. Antiretroviral-drug resistance among patients recently infected with HIV. N Engl J Med. 2002 Aug 8;347(6):385-94.

**Lockman S, A5208/OCTANE Study Team. Lopinaivr/ritonavir + tenofovir/emtricitabine is superior to nevirapine + tenofovir/emtricitabine for women with prior exposure to single-dose nevirapine: A5208 (“OCTANE”). 16^th^ Conference on Retroviruses and Opportunistic Infections, February 2009. Montreal, Canada. Abstract 94LB.**

Louie M, Hurley A, Flaherty J, et al. Determining the relative efficacy of tenofovir DF using frequent measurements of HIV-1 RNA during a short course of monotherapy in antiretroviral drug naive individuals. In: Program and abstracts of the 9th Conference on Retroviruses and Opportunistic Infections; 2002 February 24-28; Seattle, WA. Abstract 3.

Lyons F, Hopkins S, McGeary A, et al. 2^nd^ IAS Conference on HIV Pathogenesis and Treatment; July 13-16, 2003; Paris. Abstract LB27.

Margot NA, Miller MD. In vitro anti-HIV combination studies of ribavirin with tenofovir and other nucleoside analogues. 2^nd^ IAS Conference on HIV Pathogenesis and Treatment, 2003; Paris, France. Abstract 980.

Martinez E, Blanco JL, Arnaiz JA, et al. Hepatotoxicity in HIV-1 infected patients receiving nevirapine-containing antiretroviral therapy. AIDS. 2001 Jul 6;15(10):1261-8.

Mellors JW, Palmer S, Nissley D, et al.  Low frequency NNRTI-resistant variants contribute to failure of efavirenz-containing regimens. 11th Conference on Retroviruses and Opportunistic Infections, February 2004, San Francisco, CA. Abstract No. 39.

Mirochnick M, Fenton T, Gagnier P, et al. Pharmacokinetics of nevirapine in human immunodeficiency virus type 1-infected pregnant women and their neonates. Pediatric AIDS Clinical Trials Group Protocol 250 Team. J Infect Dis. 1998 Aug;178(2):368-74.

Molina JM, Gathe J, Lim PL, et al. Comprehensive resistance testing in antiretroviral-naïve patients treated with once-daily lopinavir/ritonavir plus tenofovir DF and emtricitabine: 48-week results from Study 418 [WePeB5701]. XV International AIDS Conference; 2004 July 11-16; Bangkok, Thailand.

Musoke P, Guay LA, Bagenda D, et al. A phase I/II study of the safety and pharmacokinetics of nevirapine in HIV 1-infected pregnant Ugandan women and their neonates (HIVNET 006). AIDS. 1999 Mar 11;13(4):479-86.

Nissley D et al. 12th Antiviral Drug Resistance Workshop, June 2003; Cabo San Lucas, Mexico. Poster 93

Nunez M, Gonzalez-Requena D, Gonzalez-Lahoz J, Soriano V. Short communication: interactions between nevirapine plasma levels, chronic hepatitis C, and the development of liver toxicity in HIV-infected patients. AIDS Res Hum Retroviruses. 2003 Mar;19(3):187-8.

Nunez M, Perez-Olmeda M, Diaz B, et al. Reduction of HBV DNA plasma levels alter addition of tenofovir in HBV/HIV-infected patients mailing or partially responding to lamivudine. XIV International AIDS Conference; 2002; Barcelona Spain. Abstract LbPeB9015.

Palella FJ Jr, Delaney KM, Moorman AC, et al. Declining morbidity and mortality among patients with advanced human immunodeficiency virus infection. HIV Outpatient Study Investigators. N Engl J Med. 1998 Mar 26;338(13):853-60.

Pao D, Andrady U, Clarke J, et al. Long-term persistence of primary genotypic resistance after HIV-1 seroconversion. J Acquir Immune Defic Snydr. 2004;37:1570-73.

The PETRA Study Team. The PETRA Trial: the efficacy of three short-course regimens of zidovudine and lamivudine in preventing early and late transmission of HIV-1 from mother to child in an African setting; a randomized, placebo-controlled trial performed in South Africa, Tanzania and Uganda. Lancet 2002: 359; 1178-86.

Phillips AN, Pradier C, Lazzarin A, et al. Viral load outcome of non-nucleoside reverse transcriptase inhibitor regimens for 2203 mainly antiretroviral-experienced patients. AIDS. 2001 Dec 7;15(18):2385-95.

Pieniazek D, Rayfield m, Hu DJ, et al. Protease sequences from HIV-1 group M subtypes A-H reveal distinct mutation patterns associated with protease resistance in protease inhibitor-naïve individuals worldwide. HIV Variant Working Group. AIDS. 2000 Jul 28;14(11):1489-95.

Podzamczer D, Ferrer E, Consiglio E, et al. A randomized clinical trial comparing nelfinavir or nevirapine associated to zidovudine/lamivudine in HIV-infected naïve patients (the Combine Study) Antiviral Ther. 2002 Jun;7(2):81-90.

Podzamczer D, Ferrer E, Gatell JM, et al. Early virologic failure with a combination of tenofovir, didanosine and efavirenz. *Antiviral Therapy* 2004 9:S172, Poster 156 presented at the 13th International HIV Drug Resistance Workshop; June 2004; Tenerife Sur, Spain.

Podzamczer D, Gathe J, Johnson M, et al. Study 418: Once-daily vs. twice-daily lopinavir/r in antiretroviral-naïve patients: 24-week results [Abstract]. 9^th^ European AIDS Conference; 2003 October 26-29; Warsaw, Poland.

Pollard RB, Robinson P, Dransfield K. Safety profile of nevirapine, a nonnucleoside reverse transcriptase inhibitor for the treatment of human immunodeficiency virus infection. Clin Ther. 1998 Nov-Dec;20(6):071-92.

Reynes J, Peyriere H, Merle de Boever C, Le Moing V. 10th Conference on Retroviruses and Opportunistic Infections; February 10-14, 2003; Boston, MA. Abstract 717.

Richman DD, Havlir D, et al. Nevirapine resistance mutations of human immunodeficiency virus type 1 selected during therapy. J Virol 1994;68:1660-6.

Riska PS, Lamson M, MacGregor T, et al. Disposition and biotransformation of the antiretroviral drug nevirapine in humans. Drug Metab Dispos. 1999 Aug;27(8):895-901.

Robbins BL, Srinivas RV, Kim C, Bischofberger N, Fridland A. Anti-human immunodeficiency virus activity and cellular metabolism of a potential prodrug of the acyclic nucleoside phosphonate 9-*R*-(2-phosphonomethoxypropyl)adenine (PMPA), Bis(isopropyloxymethylcarbonyl) PMPA. Antimicrob Agents Chemother 1998 Mar;42 (3):612-7

Robbins G, de Gruttola V, Shafer RW, et al. Comparison of sequential three-drug regimens as initial therapy for HIV-1 infection. N Engl J Med. 2003;349(24): 2293-2303.

Robbins G, Shafer R, Smeaton L, et al. Antiretroviral strategies in naïve HIV+ participants: comparison of sequential 3-drug regimens (ACTG 384). XIV International AIDS Conference; 2002; Barcelona Spain. Abstract LBOr20a.

Rousseau FS, Kahn JO, Thompson M, et al. Prototype trial design for rapid dose selection of antiretroviral drugs: an example using emtricitabine (Coviracil). J Antimicrob Chemother 2001; 48:507-513

Rousseau FS, Wakeford C, Mommeja-Marin H, et al. FTC-102 Clinical Trial Group. Prospective randomized trial of emtricitabine versus lamivudine short-term monotherapy in human immunodeficiency virus-infected patients. J Infect Dis. 2003 Dec 1;188(11):1652-8.

Saag M, Cahn P, Raffi F, et al. Efficacy and safety of emtricitabine vs stavudine in combination therapy in antiretroviral-naive patients: a randomized trial. JAMA 2004; 292:180-189.

Sanne I, Mommeja-Marin H, Hinkle J, et al. Severe hepatotoxicity associated with nevirapine use in HIV-infected subjects. J Infect Dis. 2005 Mar 15;191(6):825-9.

Schinazi RF, McMillian A, Cannon D et al. Selective inhibition of human immunodeficiency viruses by racemates and enantiomers of cis-5-fluoro-1[2-(hydroxymethyl)-1,3-oxathiolane-5-yl}cytosine. Antimicrob Agents Chemother 1992; 36:24232431

Schinazi RF. Assessment of the relative potency of emtricitabine and lamivudine. J Acquir Immun Defic Syndr 2003; 34:243-245

Schinazi RF, Lloyd RM, NguyenM-H, et al. Characterization of human immunodeficiency viruses resistant to oxathiolane-cytosine nucleosides. Antimicrob Agents Chemother. 1993; 37:875-881

Schooley RT, Ruane P, Myers RA et al Tenofovir DF in antirtroviral-experienced patients: results from a 48-week, randomized, double-blind study. AIDS. 2002; 16:1257-63.

Shafer RW, Smeaton LM, Robbins GK, et al. Comparison of four-drug regimens and pairs of sequential three-drug regimens as initial therapy for HIV-1 infection. New Eng J Med. 2003;349(24):2304-15.

Shaffer N, Chuachoowong R, Mock PA, et. al., Short-course zidovudine for perinatal HIV-1 transmission in Bangkok, Thailand: a randomized controlled trial. Lancet 1999; 353: 773-80.

Sperling RS, Shapiro DE, Coombs RW, et al. Maternal viral load, zidovudine treatment, and the risk of transmission of human immunodeficiency virus type 1 from mother to infant. Pediatric AIDS Clinical Trials Group Protocol 076 Study Group. N Engl J Med 1996; 335: 1621-9.

Squires K, Pozniak AL, Pierone G Jr, et al. Tenofovir disoproxil fumarate in nucleoside-resistant HIV-1 infection: a randomized trial. Ann Intern Med 2003; 139:I22.

Staszewski S, Gallant J, Pozniak AL, et al. Efficacy and safety of tenofovir disoproxil fumarate (TDF) versus stavudine (d4T) when used in combination with lamidvudine (3TC) and efarvirenz (EFV) in HIV-1 infected patients naïve to antiretroviral therapy (ART): 48-week results. XIV International AIDS Conference, July 71-12, 2002; Barcelona Spain. Abstract LBOr17.

Staszewski S, Morales-Ramirez J, Tashima KT et al. Efavirenz plus zidovudine and lamivudine in the treatment of HIV-1 infection in adults. Study 006 team. N Engl J Med 1999; 341:1865-73.

Stringer JS, Sinkala M, Stout JP, et al. Comparison of two strategies for administering nevirapine to prevent perinatal HIV transmission in high-prevalence, resource-poor settings. J Acquir Immune Defic Syndr. 2003;32(5):506-13.

Sullivan J. South African Intrapartum Nevirapine Trial: selection of resistance mutations. XIV International AIDS Conference, July 71-12, 2002; Barcelona, Spain. Abstract LbPeB9024.

Szczech GM, Wang LH, Walsh JP, Rousseau FS. Reproductive toxicology profile of emtricitabine in mice and rabbits. Reprod Toxicol*.* 2003;17(1):95-108.

Tarantal AF, Castillo A, Ekert JE, Bischofberger N, Martin RB. Fetal and maternal outcome after administration of tenofovir to gravid rhesus monkeys (Macaca mulatta). J Acquir Immune Defic Syndr 2002;29:207-20.

Turner D, Brenner B, Wainberg MA. Relationships among various nucleoside resistance-conferring mutations in the reverse transcriptase of HIV-1. J Antimicrob Chemother. 2004 Jan;53(1):53-7.

Van Leeuwen R, Katlama C, Murphy RL, et al. A randomized trial to study first-line combination therapy with or without a protease inhibitor I HIV-1-infected patients. AIDS. 2003 may 2;17(7):987-99.

van Leth F, et al. Results of the 2NN study: a randomized comparative trial of first-line antiretroviral therapy with regimens containing either nevirapine alone, efavirenz alone or both drugs combined, together with stavudine and lamivudine. 10th Conference on Retroviruses and Opportunistic Infections. February 10-12, 2003; Boston, MA. Abstract 176.

Wakeford C, Shen G, Hulett L, Quinn JB, Rousseau F. Long-term efficacy and safety of emtricitabine in HIV+ adults switching from a lamivudine containing HAART regimen. Paper presented at: 10th Conference on Retroviruses and Opportunistic Infections; February 10-14, 2003; Boston, MA.

Walmsley S, Bernstein B, King M, et al. Lopinavir-ritonavir versus nelfinavir for the initial treatment of HIV infection. N Engl J Med. 2002; 346(26):2039-46.

Wang LH, Blum MR, Hui J et al. Lack of significant pharmacokinetic interactions between emtricitabine (Coviracil) and other nucleoside antivirals in healthy volunteers. In Program and Abstracts of the 41^st^ Interscience Conference on Antimicrobial Agents and Chemotherapy. 2001; Chicago, IL. Abstract A505.

Wang LH, Begley J, Feng JY, Quinn J, and Rousseau FS. Pharmacokinetic and pharmacodynamic characteristics of emtricitabine support its once daily dosing. In Program and Abstracts of the XIV International AIDS Conference, July 71-12, 2002; Barcelona, Spain, abstract 4546.

World Health Organization. Scaling up antiretroviral therapy in resource-limited settings: Treatment guidelines for a public health approach*.* 2003 Revision, October 2003. World Health Organization, Geneva, Switzerland.

Zhou XJ, Sheiner LB, D’Aquila, et al. Population pharmacokinetics of nevirapine, zidovudine, and didanosine in human immunodeficiency virus-infected patients. The National Institute of Allergy and Infectious Diseases AIDS Clinical Trials Group Protocol 241 Investigators. Antimicrob Agents Chemother. 1999 Jan;42(1):121-8.

APPENDIX I

DIVISION OF AIDS

AIDS CLINICAL TRIALS GROUP (ACTG)

### SAMPLE INFORMED CONSENT

A5208/OCTANE

Optimal Combination Therapy After Nevirapine Exposure

FINAL Version 3.0, dated 12/11/06

ALTERNATE TITLE FOR THE STUDY: NNRTI and PI-based ARV Treatment in Women With/Without Single-Dose NVP MTCT Prophylaxis, FINAL Version 3.0, dated 12/11/06

INTRODUCTION

This is a consent form for a research study that is paid for by the National Institutes of Health (NIH) of the United States of America (U.S.). The doctor in charge of this study at this site is: *(name of site’s Principal Investigator)*. Before you decide if you want to be a part of this study, we want you to know all about it. You are being asked if you want to participate in this study because you are infected with HIV (the virus that causes AIDS) and you have not taken anti-HIV drugs on a regular basis.

You are free to ask questions about this study at any time. If you agree to take part in this study, you will be asked to sign this consent form and you can keep a copy.

Please note that:

- It is entirely your choice whether or not you participate in this study.
- You may stop taking part in the study at any time.
- You will still receive your standard health care if you do not participate in this study.

WHY IS THIS STUDY BEING DONE?

We think that some drugs used to treat HIV may not work as well for women who took nevirapine (NVP) as they do for women who did not, but we do not know this for sure. Many pregnant women take NVP to help their babies stay healthy.

The purposes of this study are:

1) to see how well two anti-HIV drug treatments work for HIV-infected women who took NVP before giving birth,

2) to see how well two anti-HIV drug treatments work for HIV-infected women who have never received NVP,

3) to learn more about the safety and side effects of the 2 treatments.

There are many anti-HIV drugs. Taking a combination of three of these drugs allows people infected with HIV to live longer and to feel healthier, although the drugs do not cure HIV/AIDS.

The main study drugs are:

- nevirapine (which is also known as NVP or Viramune)
- lopinavir/ritonavir (which is also known as LPV/RTV, Kaletra, or Aluvia)
- emtricitabine (which is also known as FTC or Emtriva)
- tenofovir (which is also known as TDF or Viread)
- FTC/TDF (which is also known as Truvada)

All of these drugs are already approved for use in the U.S., and are commonly used to treat HIV/AIDS in the U.S. and in many places in the world. FTC and TDF may be taken as a combination pill (Truvada) or may be taken individually.

All of the study drugs named above will be provided through the study. Three other approved drugs that are commonly used to treat HIV/AIDS, zidovudine (which is also known as ZDV, AZT, or Retrovir), didanosine EC (which is also known as ddI EC or Videx EC), and efavirenz (which is also known as EFV or Stocrin), will also be provided through the study in case you need to change some of your drugs later in the study.

WHAT DO I HAVE TO DO IF I AM IN THIS STUDY?

- Come to the clinic for 9 visits during the first year.
- Come to the clinic every 3 months after the first year.
- Come to the clinic if side effects occur.

Screening Visit

If you give your consent on this form, you will undergo screening tests to make sure that you can take part in the study. These tests will take about 1.5 hours.

Entry Visit

If all of your screening tests show that you can join this study, you will return to the clinic to enter the study. The tests and procedures at this visit will take about 1 hour.

At this visit, you will be placed into one of two treatment groups, Group A or Group B. Your chances of being placed in either Group A or Group B are equal because you will be assigned by chance, as if by the toss of a coin. We do not know whether the treatment in one group is any more effective than the other; the purpose of the study is to answer this question. You, the study nurse, and your doctor will all know which treatment group you are in.

The treatment groups are:

Group A Group B

NVP once a day in the morning for 14-17 days, LPV/RTV twice a day PLUS

then twice a day PLUS FTC once a day PLUS

FTC once a day PLUS TDF once a day

TDF once a day

If the combination capsule FTC/TDF is available in your area, you may take it instead of taking FTC and TDF separately.

Visit schedule and what will be done during those visits

- Screening Visit - You will undergo screening blood tests and a physical examination to make sure that you can take part in the study. About 2 tablespoons (about 30 mL) of your blood will be collected for these screening tests. This visit will take about 1.5 hours.
- Entry Visit - If all of your screening tests show that you can join this study, you will return to the clinic to enter the study. You will have about 4 tablespoons (about 60 mL) of blood drawn for tests, a physical examination, and will be asked to answer some questions about your health and medications. Some blood will be drawn for storage for later study-related testing. At this visit you will be assigned to your treatment group. This visit will take about 1 hour.
- Study Visits - 2 weeks after entry, then 1, 2, 3, 4, and 6 months after starting treatment. After your 6 month visit, you will come to the clinic every 3 months for up to 1.5 years. At these visits, you will have between 1.5 and 3 tablespoons (between 23 and 45 mL) of blood drawn for tests, a physical examination, and answer some questions about your health and medications. Some blood will be drawn for storage for later study-related testing. These visits will take about 1.5 hours.

* If you are in Group A, you will have an extra teaspoon (about 5 mL) of blood drawn at the 2 week and 1 month visits, and will be asked to keep track of when you take your NVP for 3 days before each of these visits and on the morning of the visit.

Any time that results of exams and laboratory tests such as routine safety tests, HIV viral level, tests of immune system, and pregnancy tests are known, they will be given to you if you like. Knowing the results may assist in your medical care.

If you decide to quit the study early, you will be asked to come to the clinic for one last visit. At this visit you will have about 3 tablespoons (about 45 mL) of blood drawn for the same lab tests, and a small amount that will be stored. You will also have most of the same procedures that you had at other visits, but you will not be asked to complete any questionnaires.

WHAT IF I HAVE TO STOP THE STUDY DRUGS?

You may need to stop taking the study drugs that you were assigned. This may be because the drugs are not helping your HIV infection or because you have severe side effects. If it seems that the drugs are not helping your HIV infection, you will be asked to come to the clinic to have about 3 tablespoons (about 45 mL) of blood collected. Some of the blood will be used to check your HIV virus level. The rest of the blood will be stored for tests, including a resistance test, which may be run at a later date. New drugs will be chosen for you.

The figure below shows how your new study drugs will be chosen if the first ones were not helping your HIV infection:

Group A Group B

NVP + TDF + FTC LPV/RTV + TDF + FTC

LPV/RTV + at least two drugs NVP + at least two drugs

You may continue on FTC and TDF if you and your doctor feel that it is appropriate. However, you may use drugs other than these if you and your doctor feel it is necessary. The choice of other drugs is up to you and your doctor and may include a combination of study-provided drugs and drugs not available through the study (see the table at the end of this form for details). You should avoid the combination of TDF, didanosine enteric coated capsules (ddI EC, Videx EC), and either NVP or EFV. Recent studies suggest that these combinations may not be effective in people who are just starting anti-HIV treatment and who have a high HIV level.

HOW OFTEN WILL I NEED TO RETURN IF MY STUDY DRUGS ARE CHANGED?

If you change NVP to LPV/RTV or LPV/RTV to NVP, the clinic visit schedule and evaluations will start over just as they were in the beginning of the study (please see the visit schedule table above). If you only change drugs other than NVP or LPV/RTV, the schedule will continue without starting over at the beginning.

WHAT IF I BECOME PREGNANT DURING THE STUDY?

First you will need to decide if you want to continue in the study. If you decide to continue you will be asked to give your consent on a Pregnancy Consent Form. You will continue on the same schedule of study visits. Your doctor may recommend changing one or more of your HIV drugs while you are pregnant for the health of your infant.

WHAT IF I DEVELOP TUBERCULOSIS (TB) DURING THE STUDY?

Your general health will be checked at each of the scheduled visits that you attend. If you develop TB while you are participating in this study and need to be treated with rifampin, you may be switched to efavirenz (which is also known as EFV, Sustiva, or Stocrin) in place of NVP or LPV/RTV. If you are not able to take EFV for any reason, you may either stay on NVP or be switched to NVP in place of LPV/RTV.

ARE THERE OTHER TESTS I SHOULD KNOW ABOUT?

Some of your stored blood will be used for viral testing that is required for this study.

Some of your blood that is left over after all necessary study testing is done may be stored. Your blood will not be identified by your name. It will be stored with usual protectors of identity. This blood will be used for DAIDS-approved HIV-related research. Please decide if you agree to have some of your blood stored for these reasons and indicate your decision by marking the appropriate space below. You may stay in the study no matter what you decide.

I agree: _______ OR I do NOT agree: ____________

HOW MANY PEOPLE WILL TAKE PART IN THIS STUDY?

About 640 women will take part in this study. There will be about 320 participants in each group.

HOW LONG WILL I BE IN THIS STUDY?

You will be in this study between 48 weeks (about 1 year) and 96 weeks (about 2 years). The exact length will depend on when you join.

WHY WOULD THE DOCTOR TAKE ME OFF THIS STUDY EARLY?

The study doctor may need to take you off the study early without your permission if:

- the study is cancelled by the U.S. National Institutes of Health (NIH), the U.S. Office of Human Research Protections (OHRP), local ministry of health, ethics committee (EC), the drug companies supporting this study, or your site’s Institutional Review Board (IRB). (An IRB is a committee that watches over the safety and rights of research participants.)
- a Data Safety Monitoring Board (DSMB) recommends that the study be stopped early. (A DSMB is an outside group of experts who monitor the study.)
- you are imprisoned or are confined against your will in a medical facility
- you are not able to attend the study visits as required by the study

The study doctor may also need to take you off the study drug(s) without your permission if:

- continuing the study drug(s) may be harmful to you
- you need a treatment that you may not take while on the study
- you are not able to take the study drug(s)

If you must stop taking the study drug(s) before the study is over, the study doctor may ask you to continue to be part of the study and return for some study visits and procedures.

WHAT ARE THE RISKS OF THE STUDY?

Risks of Blood Drawing

Taking blood may cause some discomfort, bleeding, or bruising where the needle enters the body, lightheadedness, and in rare cases, fainting or infection.

Risks of Study Drugs

It is possible that the study drugs will make you feel sick or will affect your blood tests, in which case the study doctor may either switch you to different drugs, or stop them all together. It is very important for you to return to the clinic whenever you feel sick. Feeling sick may be due to the study drugs or it may be due to a sickness caused by your HIV infection. Either way, we want to see you when you feel sick so we can take care of you.

All anti-HIV drugs can cause side effects, which can be more serious or severe with long‑term use. Some of these side effects are mild and may go away after you have taken the drugs for a few weeks. Examples of these types of side effects include upset stomach, vomiting, headache, and changes in your mood, sleep, or concentration.

The use of potent antiretroviral drug combinations may be associated with an abnormal placement of body fat and wasting. Some of the body changes include:

- Increase in fat around the waist and stomach area
- Increase in fat on the back of the neck
- Thinning of the face, legs and arms
- Breast enlargement

Other side effects are severe and may require treatment or hospitalization. Examples of these types of side effects include rash or liver problems. Severe liver damage that can cause death may occur, particularly with NVP. You may notice the following if you have severe liver damage:

- Tiredness
- General feeling of illness
- Loss of appetite
- Nausea
- Pale stools
- Dark urine
- Yellowing of the skin or whites of your eyes
- Liver tenderness or abnormal liver function tests

There is an important side effect called a severe hypersensitivity reaction (HSR) that may also occur with NVP, one of the study drugs. HSR is rarely fatal. This reaction may be associated with the following:

- rash
- fever
- fatigue
- muscle or joint aches
- blisters
- mouth sores
- facial swelling
- red eyes and irritation of the eyes
- general feeling of discomfort
- hepatitis
- kidney problems
- changes in white blood cell levels.

The risk of people developing any of the serious side effects listed above is greatest during the first few months of treatment, but these side effects also can occur later. If you develop any of the side effects listed above, no matter how long you have been receiving NVP, you must contact your health care provider right away and try to be seen by the medical staff at your site before your next dose. If you and your doctor then decide to stop your treatment because of symptomatic hepatitis, hypersensitivity or severe skin reactions, you should never take NVP again.

Rarely, some people taking HIV drugs can develop a condition called “lactic acidosis.” Some symptoms that might be caused by lactic acidosis include:

- unexplained weight loss
- stomach upset
- nausea
- vomiting
- fatigue
- weakness
- shortness of breath
- muscle pain
- cramps
- dizziness.

Lactic acidosis, along with an enlarged and fatty liver, may result in problems such as liver failure. In some cases, the condition results in death. The liver problems and death have been seen more in women on these drug regimens.

It is very important that you know side effects might occur when you take new medications. At the end of this consent form, there is a table that describes the side effects for anti‑HIV drugs that you may receive during this study. When you get your study drugs, you will be told the possible side effects. Throughout the study, these side effects will be told to you, particularly if you receive a new anti-HIV drug. If you receive an anti-HIV drug that is not listed in the table, the study doctor will make sure that you understand the side effects of the drug. If you have questions concerning study drug side effects, please ask us at any time.

Other Risks of Study Drugs

After you begin taking the anti-HIV drugs, do not stop taking any of them unless you discuss it with the study doctor. Suddenly stopping your treatment can cause an increase in the amount of HIV in your blood, and the virus can become resistant to anti-HIV drugs, which means that the drugs will no longer work.

There is sometimes a risk of serious and life-threatening side effects when non-study medications or traditional treatments are taken with study drugs. For your safety, you must tell the study doctor about all medications you are taking before you start the study and before taking any non-study medications while you are on the study.

Risks of Treatment Failure

There is a possibility that the HIV treatment that you are assigned may not work for you. If this happens, you and your doctor may choose to start a second treatment.

Additionally, there is a possibility that if you took NVP in the past to prevent your baby from getting HIV, a future treatment for your HIV infection may not work as well if the treatment includes NVP instead of another drug such as LPV/RTV. This is the question that we are trying to answer with this study. It is also not known if the number of times you took NVP or the amount of time since your last NVP dose changes this possibility.

ARE THERE RISKS RELATED TO PREGNANCY?

It is not known if the drugs in this study harm unborn babies. If you are having sex that could lead to pregnancy, you must agree not to become pregnant.

Some of the drugs, including LPV/RTV and NVP, in this study make some birth control drugs that are estrogen-based less effective. This type of birth control is given by pills, shots, or placed on or under the skin. This means that you cannot depend on this method of birth control alone when taking these drugs. You must use a different method or an additional method of birth control that you discuss with the site staff.

As applicable, at least one of the following methods MUST be used correctly:

- Condoms (male or female) with or without a spermicidal agent
- Diaphragm or cervical cap with spermicide
- Intrauterine device (IUD)
- Birth control drugs that prevent pregnancy given by pills, shots or placed on or under the skin.

You and your partner must use reliable birth control. This must be discussed with the study staff. You must continue to use birth control until 6 weeks after stopping study drugs.

If you can become pregnant, you must take a pregnancy test before you enter this study. The test must show that you are not pregnant. If you think you may be pregnant at any time during the study, tell us right away. We will talk to you about your choices.

We know that tests done in pregnant animals taking EFV show some sign of harm to unborn babies (please see the risk table at the end of this consent form). Because of this, you and your partner must use TWO methods of birth control if you are receiving EFV (which will be recommended if you develop tuberculosis during the study). You will also have a pregnancy test once a month while taking EFV and again 1 month after you stop EFV. You must continue to use both methods of birth control until 6 weeks after stopping EFV. You may choose two of the birth control methods listed below:

- Birth control drugs that prevent pregnancy given by pills, shots or placed on or under the skin
- Male or female condoms with or without a cream or gel that kills sperm
- Diaphragm or cervical cap with a cream or gel that kills sperm
- IUD

ARE THERE BENEFITS TO TAKING PART IN THIS STUDY?

Treatment with the study drugs or with other combinations of HIV drugs is known to prolong the life and improve the health of persons with HIV. The study drugs (and other combinations of HIV drugs) are recommended and used throughout the world for HIV-infected people with low levels of immune cells (CD4 less than 200). If you take part in this study, there may be a direct benefit to you, but no guarantee can be made. It is also possible that you may receive no benefit from being in this study. Information learned from this study may help others who have HIV.

WHAT OTHER CHOICES DO I HAVE BESIDES THIS STUDY?

Instead of being in this study you have the choice of:

- treatment with prescription drugs available to you
- treatment with experimental drugs, if you qualify
- no treatment

Please talk to your doctor about the choices available to you. Your doctor will explain the risks and benefits of these choices.

WHAT ABOUT CONFIDENTIALITY?

The study team will provide you with an identification number. This will be used for laboratory tests or blood work stored for later testing. Efforts will be made to keep your personal information confidential. We cannot guarantee absolute confidentiality. Your medical records, name, address, and identification number will be kept in a locked room. Only the study staff will have the keys. No publication of this study will use your name or identify you personally.

Your personal information may be revealed if required by law. Your records may also be reviewed by *(insert name of site IRB*), ethics committee, U.S. National Institutes of Health (NIH), your country’s national health agency, study staff, study monitors, and drug companies supporting this study.

WHAT ARE THE COSTS TO ME?

You will not pay for study-related visits, study drugs, physical examinations, laboratory tests, or other procedures. Taking part in this study may lead to added costs to you and if you have insurance *(delete reference to insurance company if not applicable at site)* to your insurance company. In some cases it is possible that your insurance company or health care system will not pay for these costs. Your insurance company or health care system may need to assume the cost of obtaining ART drugs that are not provided by the study. It is possible that you will be responsible for the cost of ART drugs that are not provided through this study.

WILL I RECEIVE ANY PAYMENT?

*(Insert site-specific information on compensation to study participants, e.g. reimbursement for travel.)*

WHAT HAPPENS IF I AM INJURED?

If you are injured because you took part in this study, you will be treated right away for your injuries. However, you may/may not *(per site/country policy)* have to pay for this care. There is no program for payment either through this institution or the U.S. National Institutes of Health (NIH). You will not be giving up any of your legal rights by signing this consent form.

WHAT ARE MY RIGHTS AS A RESEARCH PARTICIPANT?

Taking part in this study is completely up to you. You may choose not to take part in this study. You may leave this study at any time. You will be treated the same no matter what you decide.

We will tell you about new information from this or other studies that may affect your health, welfare, or willingness to stay in this study. If you want the results of the study, let us know.

WHAT DO I DO IF I HAVE QUESTIONS OR PROBLEMS?

For questions about this study or a research-related injury, contact:

- name of the investigator or other study staff
- telephone number of above

For questions about your rights as a research participant, contact:

- name or title of person on the Institutional Review Board (IRB) or other organization appropriate for the site
- telephone number of above

SIGNATURE PAGE

If you have read this consent form (or had it explained to you), all your questions have been answered, and you agree to take part in this study, please sign your name below.

_____________________ __________________________________________

Participant’s Name (print) Participant’s Signature and Date

____________________________ __________________________________________

Participant’s Legal Guardian (print) Legal Guardian’s Signature and Date

(As appropriate)

________________________ _________________________________________

Study Staff Conducting Study Staff Signature and Date

Consent Discussion (print)

________________________ _________________________________________

Witness’s Name (print) Witness’s Signature and Date

(As appropriate)

| Potential Side Effects of Antiretroviral Drugs Provided by A5208 / OCTANE Study | |
| --- | --- |
| Anti-HIV Drug | Side Effects |
| Nevirapine (NVP, Viramune®) | Severe liver damage that can result in death may occur and is often associated with a rash. People, especially women, with higher CD4 cell counts are at increased risk for developing liver damage.  Women with CD4 cell counts greater than 250, including pregnant women receiving chronic nevirapine therapy, are at greatest risk for developing liver damage. Men with CD4 cell counts greater than 400 are also at increased risk. These reactions can happen at any CD4 count. People who have abnormal liver function tests before starting nevirapine and people with active Hepatitis B or C infection are also at higher risk for liver damage.  If you are developing liver damage, you may have one or more of the following:   - Tiredness - General feeling of illness - Loss of appetite - Nausea - Pale stools - Dark urine - Yellowing of the skin or whites of your eyes - Liver tenderness or abnormal liver function tests   Hypersensitivity reactions (“allergic reaction”), which can rarely be fatal, may occur. The symptoms that you may notice are: rash, fever, fatigue, muscle or joint aches, blisters, mouth sores, facial swelling, red eyes and irritation of the eyes, general feeling of discomfort, hepatitis, kidney problems, and/or changes in white blood cell levels.  Rash is the most common side effect associated with Nevirapine. Rash occurs more often in women. Most rashes occur early during treatment. The rash may be severe and rarely may cause death. One of the risk factors for developing serious skin reactions includes failure to take nevirapine properly during the first 14 days of treatment.  The risk of people developing any of the serious side effects listed above is greatest during the first few months of treatment, but these side effects also can occur later. If you develop any of the side effects listed above, no matter how long you have been receiving nevirapine, you must contact your health care provider right away and try to be seen by the medical staff at your site before your next dose. If you and your doctor then decide to stop your treatment because of liver damage, hypersensitivity or severe skin reactions, you should never take Nevirapine again.  Other than the serious side effects listed above, additional side effects include:   - Fever - Headache - Upset stomach |
| Lopinavir/Ritonavir (LPV/RTV, KaletrA^TM^, or ALUVIA®) | - Pancreatitis (inflammation of the pancreas), which may cause death. If you develop pancreatitis, you may have one or more of the following: stomach pain, nausea, vomiting or abnormal pancreatic function blood tests - Abnormal bowel movements (stools), including loose or watery stools, upset stomach and stomach pain - Large increases in triglycerides and cholesterol in the blood - Liver problems and worsening liver disease, which may result in death. People with these conditions may have abnormal liver function blood tests - Feeling weak and tired - Headache - Rash (seen in children) |
| Emtricitabine (FTC, Emtriva™) | - Headache - Dizziness - Tiredness - Inability to sleep, unusual dreams - Loose or watery stools - Upset stomach (nausea) or vomiting - Abdominal pain - Rash, itching, which sometimes can be a sign of an allergic reaction - Skin darkening of the palms and/or soles - Increased cough - Runny nose - Abnormal liver function tests, which could mean liver damage - Increases in pancreatic enzyme (substances in the blood), which could mean a problem with the pancreas - Increased triglycerides - Increased creatine phosphokinase (CPK), which could mean muscle damage   NOTE: If you are infected with both Hepatitis B and HIV, you should be aware that your liver function tests may increase, and symptoms associated with hepatitis (an acute inflammation of the liver) may worsen if emtricitabine is stopped. |
| Tenofovir Disoproxil Fumarate (Tenofovir DF, TDF, VIREAD®) | - Upset stomach, vomiting, gas, loose or watery stools - Dizziness - Abdominal pain - Lack of energy - Kidney damage or failure - Inflammation or swelling and possible damage to the pancreas - Shortness of breath - Rash - Low phosphate, a chemical in the blood - Increase of liver functions tests in children - Allergic reaction, which may include fever, rash, upset stomach, vomiting, loose or watery stools, abdominal pain, achiness, shortness of breath or a general feeling of illness - Changes in bone growth and strength were seen in study animals given tenofovir. Bone thinning has been seen in adults. In children, some decrease in bone thickness (density) has been seen.   NOTE: If you are infected with both Hepatitis B and HIV, you should be aware that your liver function tests may increase, and symptoms associated with hepatitis (an acute inflammation of the liver) may worsen if tenofovir is stopped.    NOTE: Because there is only a small amount of information on tenofovir in pregnant women, tenofovir should be used during pregnancy only if clearly needed. |
| Emtricitabine, FTC/Tenofovir Disoproxil Fumarate, TDF (TRUVADA™) | No new or unexpected side effects are observed with the FTC 200 mg/TDF 300 mg combination tablet than those observed when each drug is given separately. |
| Zidovudine (ZDV, Retrovir®) | - Decrease in the number of white blood cells that help fight infection - Decrease in the number of red blood cells that may cause weakness, dizziness, and fatigue - Muscle aches, weakness, and wasting - Headache - Upset stomach - Vomiting - Decrease in appetite - Vague overall feeling of discomfort - Lack of energy - Feeling tired - Sleeplessness - Hearthburn |
| Efavirenz (EFV, SUSTIVA) | Effects on mental function include:   - Dizziness - Trouble sleeping such as inability to sleep, abnormal dreams, and drowsiness - Confusion - Difficulty concentrating - Hallucinations - A feeling of strangeness and losing touch with reality - An exaggerated feeling of well-being - Agitation or anxiety   If alcohol or mind- or mood-altering drugs are used with efavirenz, it is possible that the above symptoms could become worse.  Serious psychiatric problems include:   - Depression, which may be severe - Suicidal thoughts or attempts (rarely) - Aggressive behavior - Psychosis-like symptoms, such as abnormal thinking, paranoia, and delusions   People with a history of psychiatric problems may be at greater risk for these serious psychiatric problems.  Other risks include:   - Rash - Upset stomach - Loose or watery stools - Headache   Increases in substance in the blood which can mean problems with the pancreas, such as inflammation or swelling of the pancreas with abdominal pain   - Increase in cholesterol - Increase in triglycerides - Abnormal liver function tests and inflammation of the liver (hepatitis) - Abnormal vision - Fever - An abnormal or unusual distribution of body fat   Efavirenz and Pregnancy:  The use of this drug during pregnancy and especially early pregnancy should be avoided. Efavirenz may cause fetal harm when taken during the first three months of pregnancy. Serious birth defects, including those of the central nervous system, have been seen in the offspring of animals and women on Efavirenz.  A false-positive urine screening test for marijuana has been seen with one particular test brand and has not been seen when using other screening tests or with tests used to confirm results for marijuana. |
| Didanosine (ddI, VIDEX®) | - Pancreatitis (inflammation of the pancreas), which may cause death. If you develop pancreatitis, you may have one or more of the following: stomach pain, nausea, and vomiting. - Deaths from liver failure have been reported in pregnant women receiving the combination of didanosine and stavudine with other anti-HIV drugs. - Numbness, tingling, and pain in the hands or feet - Abnormal vision changes - Upset stomach, vomiting and loose or watery stools - Headache - Abnormal pancreatic function blood tests or abnormal liver function blood tests - Increase in uric acid in the bloodstream   When didanosine is used with other medicines with similar side effects, these side effects may be seen more often and may be more severe than when didanosine is used alone.   - People who take didanosine together with stavudine, with or without hydroxyurea, may be at greater risk for pancreatitis or liver problems or both. These conditions may result in death. |

APPENDIX II

DIVISION OF AIDS SAMPLE INFORMED CONSENT

For

WOMEN WHO BECOME PREGNANT WHILE ON STUDY

For protocol:

A5208/OCTANE

Optimal Combination Therapy After Nevirapine Exposure

FINAL Version 3.0, dated 12/11/06

ALTERNATE TITLE FOR THE STUDY: NNRTI and PI-based ARV Treatment in Women With/Without Single-Dose NVP MTCT Prophylaxis, FINAL Version 3.0, dated 12/11/06

INTRODUCTION

Because you are now pregnant, you are being asked if you want to continue taking part in this research study. This study was designed so that women who were pregnant could not join the study. However, because you were already in the study when you became pregnant, you will be allowed to stay in the study whether or not you continue study drugs during your pregnancy.

This is a consent form. It gives you more information about this study and how it may affect your pregnancy and your baby. The study staff will talk with you about this information. You may also talk with your own doctor about what is best for you and your baby. If you agree to stay in this study, you will be asked to sign this consent form. You will get a copy to keep. You are free to ask questions of the study staff at any time.

WHAT DO I HAVE TO DO IF I STAY IN THIS STUDY?

If you choose to stay in this study, you will continue to have study visits and tests as stated in the main study A5208/OCTANE consent form.

While you are pregnant, you may substitute zidovudine (also known as ZDV or Retrovir) for TDF. ZDV will be provided to you through the study during your pregnancy. If you cannot tolerate ZDV, you may take another related drug such as didanosine (also known as ddI or Videx), stavudine (also know as d4T or Zerit), or TDF, with your doctor’s approval.

While you are pregnant, you may continue to receive FTC and either NVP or LPV/RTV, based on the study drug you were taking at the time of pregnancy. If you are taking EFV at the time of your pregnancy, you will substitute either NVP or LPV/RTV or an alternate drug (at your doctor’s discretion) for EFV.

If you are taking FTC/TDF and choose to substitute another drug for TDF, you may discontinue the combination drug and take FTC by itself.

Care related to your pregnancy, the delivery of your baby, or the care of your baby will not be provided by this study. You must arrange for your care and your baby's care outside of this study.

Long-term follow-up is recommended for a baby whose mother takes anti-HIV drugs during pregnancy. The study staff will talk with you about long-term follow-up and the possibility of enrolling your baby in a long-term follow-up study.

WHAT ARE THE RISKS RELATED TO STAYING IN THE STUDY?

Now that you are pregnant, there are some possible risks you should know. These possible risks to you and your baby are in addition to the risks that are described in the A5208/OCTANE study consent you already signed.

Risks to You if Staying on Study Drug(s):

1. Different side effects or more severe side effects may occur in pregnant women taking drugs. This may make it more difficult for you to take your study drug(s). Not taking anti-HIV study drug(s) as directed may cause the drug(s) not to work on the HIV in your blood.
2. The amount of drug in the blood may change during pregnancy. This possibly means that your level of anti-HIV drugs may decrease and not work as well or cause the HIV to become resistant to drugs.
3. It is not known if some risks of pregnancy might be made worse by study drug(s) and may result in death.

Risks to Your Baby if Staying on Study Drug(s):

1. It is not known if some study drug(s) may cause you to have a baby that is born early or dead.
2. It is not known if some study drug(s) may cause your baby to be sick or have birth defects. Not all birth defects are seen at birth. Some birth defects are seen later as the baby grows.
3. In the U.S., only Zidovudine (ZDV, Retrovir) is approved by the FDA to decrease the risk of passing HIV from mother to baby. The U.S. Public Health Service recommends that women discuss with their doctor the use of ZDV alone and with other anti-HIV drugs to decrease the risk of passing HIV to your baby.

BREAST-FEEDING

If you are still on study after delivery and you decide to breast-feed your baby, you may take ZDV instead of TDF during the time that you breast-feed, but will continue the other study drugs that you were taking during pregnancy. If you are still on study after you have stopped breastfeeding, you may go back to taking TDF instead of ZDV. If you cannot tolerate ZDV, you may take another related drug such as ddI, d4T, or TDF, with your doctor’s approval.

Researchers know that HIV can pass through breast-milk. Taking anti-HIV drugs has not been proven to decrease the chance of passing HIV through your breast milk to your baby. It is also not known whether anti-HIV drugs pass through breast milk and whether they may cause harm to your baby.

ARE THERE BENEFITS TO STAYING IN THIS STUDY?

If you continue to take part in this study, there may be a benefit to you and your baby, but no guarantee can be made. It is also possible that you and your baby will receive no benefit from continuing in this study. Information learned from this study may help others who have HIV.

Anti-HIV drugs, whether taken as part of this study or through the government antiretroviral treatment (ART) program, can help decrease the chance of your baby becoming infected with HIV during pregnancy and delivery. These medications are used throughout the world for this purpose.

WHAT OTHER CHOICES DO I HAVE SINCE I CANNOT STAY ON STUDY DRUGS?

Instead of staying on the study drugs you have the choice of:

- treatment with prescription drugs available to you
- treatment with experimental drugs being studied for use during pregnancy, if you qualify
- no treatment

Please talk to your doctor about these and other choices available to you. Your doctor will explain the risks and benefits of these choices.

WHAT ABOUT CONFIDENTIALITY?

The study team will provide you with an identification number. The identification number (not your name or other information that could be used to identify you) will be used for laboratory tests or blood work stored for later testing. Efforts will be made to keep your personal information confidential. We cannot guarantee absolute confidentiality. Your medical records, name, address, and identification number will be kept in a locked room. Only the study staff will have the keys. No publication of this study will use your name or identify you personally.

Your personal information may be disclosed if required by law. Your records may also be reviewed by (insert name of site IRB), ethics committee, U.S. National Institutes of Health (NIH), your country’s national health agency, study staff, study monitors, and drug companies supporting this study.

WHAT ARE THE COSTS TO ME?

In addition to any costs that are described in the study consent you already signed; this study will not cover any cost related to your pregnancy, delivery of your baby, or care of your baby.

WILL I RECEIVE ANY PAYMENT?

*[Sites will insert information if there is to be any payment.]*

WHAT HAPPENS IF MY BABY OR I AM INJURED?

If your baby or you are injured as a result of being in this study, you will both be given immediate treatment for your injuries. However, you may have to pay for this care *(per site policy)*. There is no program for compensation either through this institution or the National Institutes of Health (NIH). You will not be giving up any of your legal rights by signing this consent form.

WHAT ARE MY RIGHTS AS A RESEARCH PARTICIPANT?

Continuing to take part in this study is completely voluntary. You may choose not to continue in this study or leave this study at any time. You will be treated the same no matter what you decide.

We will tell you about new information from this or other studies that may affect your health, welfare or willingness to stay in this study. If you want the results of the study, let the study staff know.

WHAT DO I DO IF I HAVE QUESTIONS OR PROBLEMS?

For questions about this study or a research-related injury, contact:

- site insert name of the investigator or other study staff
- site insert telephone number of above

For questions about your rights as a research participant, contact:

- site insert name or title of person on the Institutional Review Board (IRB) or other organization appropriate for the site
- site insert telephone number of above

SIGNATURE PAGE

If you have read this consent form (or had it explained to you), all your questions have been answered and you agree to take part in this study, please sign your name below.

_____________________ __________________________________________

Participant’s Name (print) Participant’s Signature and Date

____________________________ __________________________________________

Participant’s Legal Guardian (print) Legal Guardian’s Signature and Date

(As appropriate)

________________________ _________________________________________

Study Staff Conducting Study Staff Signature and Date

Consent Discussion (print)

________________________ _________________________________________

Witness’ Name (print) Witness’s Signature and Date

(As appropriate)

**Appendix III: ADDENDUM TO**

**SAMPLE INFORMED CONSENT**

**DIVISION OF AIDS**

**AIDS CLINICAL TRIALS GROUP (ACTG)**

**A5208/OCTANE, Step 3**

**Optimal Combination Therapy After Nevirapine Exposure**

**FINAL Version 4.0, dated 04/13/09**

**INTRODUCTION**

**You are being asked to take part in Step 3, an extension of this research study, because you are currently enrolled in A5208/OCTANE. Your participation in the extension is completely voluntary. About 600 women now in A5208/OCTANE might enroll in this extension.**

**WHY IS THIS STUDY EXTENSION BEING DONE?**

**This extension is being added so that we can follow your health after you leave a research study and are no longer receiving regular HIV treatment through that study.**

**HOW WILL I RECEIVE HIV TREATMENT?**

**In this extension, you will not receive your regular HIV treatment (in other words, your anti-HIV drugs and your HIV-related clinical care) through the A5208/OCTANE study. It is expected that you will receive your treatment through a local clinic. The next section gives you more information about what will happen during this extension.**

**WHAT DO I HAVE TO DO IF I AM IN THIS EXTENSION?**

**You will enter the extension at the same time that you have your last visit in the current study. At the last visit in the current study, you will be expected to return all unused study-provided anti-HIV drugs that you have.**

**You will be asked to come back to the clinic just two times for A5208/OCTANE study visits: 12 weeks (or about 3 months) after you enter the extension and then again 72 weeks (or about a year and a half) after you enter the extension.**

**Because there will be a long time between these study visits, you will be contacted by phone or through some other means that you and the study staff agree on about 48 weeks (or about 1 year) after you enter the extension.**

**At each of these visits, we will ask you questions about your health and medications, including the anti-HIV drugs that you have been taking. We will also ask you questions about the HIV care that you are receiving outside of the study. As part of this study, we may also need to review your non-study medical records and speak with your non-study care providers, to find out more about the HIV care that you are receiving and your medical problems, and to check results of lab tests that were done at your local clinic.**

**At the extension entry visit, we will do a pregnancy test and store blood for possible future testing of your HIV virus for drug resistance.**

**At the 12- and 72-week visits, we will draw about 30mL (two tablespoons) of blood for safety tests, HIV virus level, and CD4 cell count (this is a test of your immune system, the system that your body uses to fight infections). We will also store some blood for possible future testing of your HIV virus.**

**Any time that results of exams and laboratory tests are known, they will be given to you if you would like to have them. Knowing the results may assist in your medical care.**

**If you decide you cannot complete all the study visits, you will be asked to come in for a final visit and will have the evaluations described above for the 12-week or 72-week visit.**

**ARE THERE OTHER TESTS I SHOULD KNOW ABOUT?**

**Some of your blood that is left over after all necessary study testing is done may be stored. This blood may be shipped out of the country for testing. Your blood will not be identified by your name. It will be stored with usual protectors of identity. This blood may be used for DAIDS-approved health research. Please decide if you agree to have some of your blood stored for these reasons and indicate your decision by marking the appropriate space below. You may stay in the study no matter what you decide.**

**I agree: _______ OR I do NOT agree: ____________**

**WHY WOULD THE DOCTOR TAKE ME OFF THIS STUDY EARLY?**

**Your doctor might have to take you off this study early without your permission if the study is stopped or cancelled.**

**WHAT ARE THE RISKS OF PARTICIPATING IN THIS EXTENSION?**

**The only risks of participating in the extension are those associated with having your blood drawn.**

**WHAT ARE THE POSSIBLE BENEFITS OF PARTICIPATING IN THIS EXTENSION?**

**By taking part in this part of the study, you may have more tests than you would normally have outside of the study; these tests may help you and your health care provider in making treatment decisions for you. It is also possible that there will be no benefit to participating in this part of the study. Your participation may help other HIV-infected study participants when they return to local care.**

**WHAT OTHER CHOICES DO I HAVE BESIDES THIS EXTENSION?**

**Taking part in this extension is completely up to you. If you do not wish to take part, you can still receive routine medical care at your local clinic.**

**WHAT ABOUT CONFIDENTIALITY?**

**Efforts will be made to keep your personal information confidential. We cannot guarantee absolute confidentiality. Your personal information may be disclosed if required by law. Any publication of this study will not use your name or identify you personally.**

**Your records may be reviewed by the (insert name of site) institutional review board or ethics committee, the U.S. National Institutes of Health (NIH), study staff, and study monitors.**

**WHAT ARE MY RIGHTS?**

**All information that was contained in the sections entitled ‘WHAT IF I AM INJURED?’ and ‘WHAT ARE MY RIGHTS AS A RESEARCH PARTICIPANT?’ in the consent form that you previously signed for this study applies during this extension.**

**WHAT ARE THE COSTS TO ME?**

**There will be no cost to you for study-related visits, physical examinations, laboratory tests or other procedures. *(Delete references to insurance company or health care system in the following if not applicable*.)You, your insurance company, or your health care system may need to assume the cost of anti-HIV drugs since they are not provided by the study. In some cases, it is possible that your insurance company or health care system will not pay for these costs because you are participating in a research study.**

**Questions or problems**

**For questions about this study or a research-related injury, contact:**

- ***insert name of the investigator or other study staff***
- ***insert telephone number of above***

**For questions about your rights as a research participant, contact:**

- ***insert name or title of person on the IRB/EC or other organization appropriate for the site***
- ***insert telephone number of above***

**SIGNATURE PAGE – Extension of A5208/OCTANE**

**If you have read this consent form (or had it explained to you), all your questions have been answered and you agree to take part in this study, please sign your name below.**

**____________________________ __________________________________________**

**Participant’s Name (printed) Participant’s Signature and Date**

**____________________________ __________________________________________**

**Name of Participant’s Legal Legal Guardian’s Signature and Date**

**Guardian (printed)**

**(As appropriate)**

**____________________________ __________________________________________**

**Name of Study Staff Conducting Study Staff Signature and Date**

**Consent Discussion (printed)**

**________________________ _________________________________________**

**Witness’s Name (printed) Witness’s Signature and Date**

**(As appropriate)**
